# Catalyst Design for Rh-Catalyzed Arene and Alkane C–H Borylation: The NHC Affects the Induction Period, and Indenyl is Superior to Cp

**DOI:** 10.1021/acs.organomet.4c00025

**Published:** 2024-03-28

**Authors:** Paul A. Morton, Abigayle L. Boyce, Anamarija Pišpek, Lennox W. Stewart, Daniel J. Ward, Bengt E. Tegner, Stuart A. Macgregor, Stephen M. Mansell

**Affiliations:** Institute of Chemical Sciences, Heriot-Watt University, Edinburgh EH14 4AS, U.K.

## Abstract

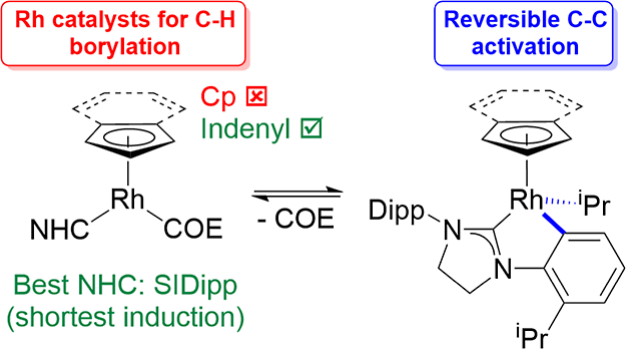

In order to establish design criteria for Rh C–H borylation catalysts, analogues of the successful catalyst [Rh(Ind)(SIDipp)(COE)] (Ind = η^5^-indenyl, SIDipp = 1,3-bis(2,6-diisopropylphenyl)-4,5-dihydroimidazol-2-ylidene, and COE = *cis*-cyclooctene) were synthesized by changing the indenyl and carbene ligands. [RhCp(SIDipp)(COE)] (**1**) formed alongside the C–C activated, cyclometalated byproduct [RhCp(κ^2^C_Ar_,C_carbene_-SIDipp′)(^*i*^Pr)] (*rac***-2**; SIDipp′ = 1-(6-isopropylphenyl)-3-(2,6-diisopropylphenyl)-4,5-dihydroimidazol-2-ylidene). Computational modeling of COE dissociation showed that both C–C and C–H activation of the SIDipp aryl group is thermally attainable and reversible under experimental conditions, with the C–C activation products being the more thermodynamically stable species. Oxidative addition of **1** with SiH(OEt)_3_ gave the Rh silyl hydride [RhCp(H){Si(OEt)_3_}(SIDipp)] (*rac***-3**). [Rh(Ind)(IDipp)(COE)] (**4**; IDipp = 1,3-bis(2,6-diisopropylphenyl)-imidazole-2-ylidene), the carbonyl analogue [Rh(Ind)(IDipp)(CO)] (**5**; ν_CO_ = 1940 cm^–1^, cf. 1944 cm^–1^ for [Rh(Ind)(SIDipp)(COE)]), and [Rh(Ind)(IMe_4_)(COE)] (**6**; IMe_4_ = 1,3,4,5-tetramethylimidazol-2-ylidene) were also characterized, but attempts to synthesize Rh carbene complexes with fluorenyl or 1,2,3,4-tetrahydrofluorenyl ligands were not successful. For the catalytic C–H borylation of benzene using B_2_pin_2_, **1** was inactive at 80 °C, and [Rh(Ind)(SIDipp)(COE)] was superior to all other complexes tested due to the shortest induction period. However, the addition of HBpin to precatalyst **4** eliminated the induction period. Catalytic *n*-alkane C–H borylation using [Rh(Ind)(NHC)(COE)] gave yields of up to 21% alkylBpin, but [RhCp*(C_2_H_4_)_2_] was the better catalyst.

## 1 Introduction

C–H activation and functionalization has been an important goal over recent decades because C–H bonds are ubiquitous in chemistry and biochemistry but are often very difficult to react directly and selectively.^1−12^ Although recent progress in the field of first row transition metal-catalyzed and metal-free C–H functionalization has been impressive,^13−20^ many catalysts for C–H activation and functionalization rely on precious metals, such as rhodium and iridium.^21−25^ Two different classes of C–H activation mediated by metals are widely recognized; directed C–H activation,^11,26^ where a donor group on the substate helps direct the reaction of a C–H bond, and undirected C–H activation,^1^ where there is no assistance from other functional groups on the substrate. Furthermore, the nature of the C–H bond is very important with arene sp^2^ C–H functionalization proving to be an easier reaction than sp^3^ functionalization, with alkanes the pinnacle of difficulty.^27,28^ This is due to the facile precoordination of the arene substrate via its π-system, among other factors.^4^

**Scheme 1:**
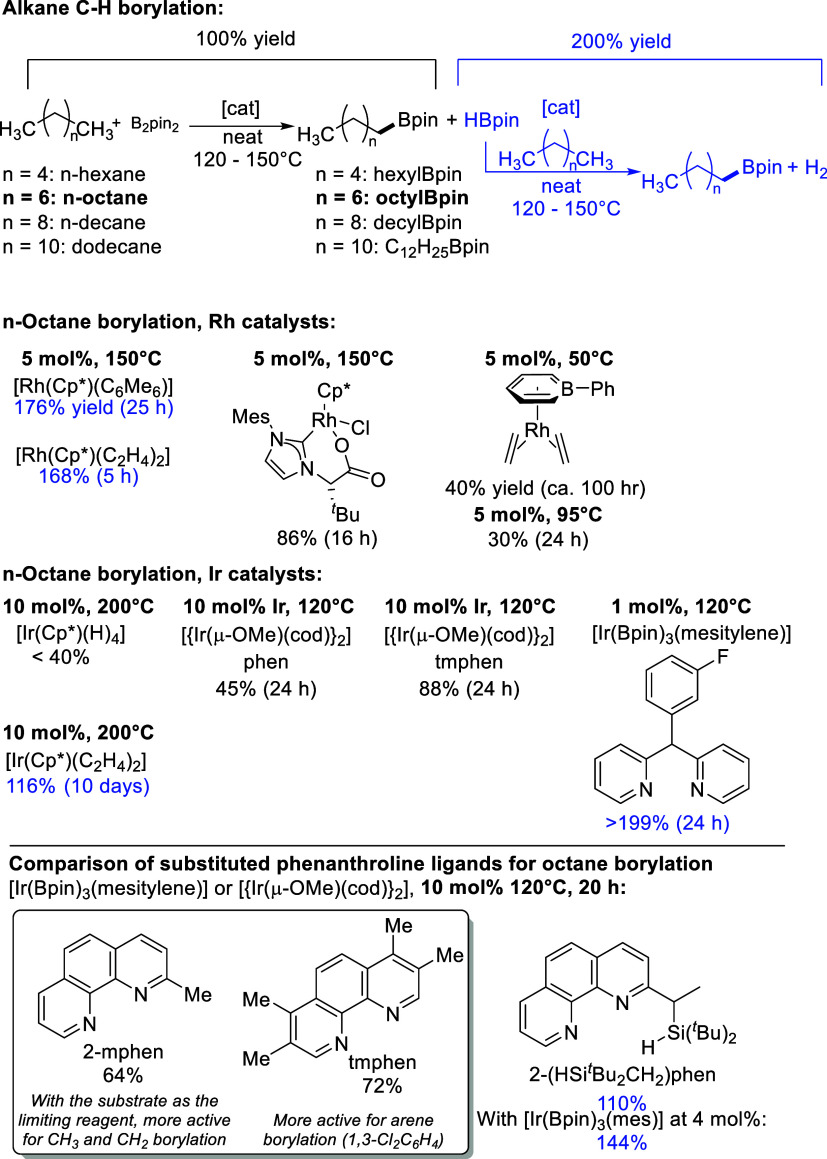
Catalytic C–H Borylation Using Precious Metal Catalysts

Seminal work in the field of catalytic C–H functionalization was published in 2000 by Hartwig and co-workers who described the first thermally driven catalytic alkane C–H borylation reaction.^29−34^ Despite this advance, C–H borylation of arenes has been more widely studied since then.^30,35,36^ Energetically, the reaction of a diboron(4) reagent,^37^ typically bis(pinacolato)diboron(4) (B_2_pin_2_), with an alkane to produce an alkylborane and HBpin has a large negative Gibbs free energy change (−13 kcal mol^–1^ for CH_4_),^32^ and the byproduct of the reaction, HBpin, can also react with an alkane to give a second equivalent of an alkylborane and dihydrogen gas in an approximately thermoneutral reaction (Scheme 1).^38,39^ This equilibrium can be driven by the loss of dihydrogen. The choice of catalysts for these reactions is very important, and two catalysts based on Rh were initially described; [RhCp*(C_6_Me_6_)] and [RhCp*(C_2_H_4_)_2_].^29^ Of the two, [RhCp*(C_2_H_4_)_2_] was found to be faster converting all B_2_pin_2_ to octylBpin and HBpin in 1 h at 150 °C (5 mol % catalyst), but heating for an additional 4 h allowed conversion of HBpin as well giving a yield of 168% octylBpin (yield based on B_2_pin_2_).^29^ Catalysis with [RhCp*(C_6_Me_6_)] was slower taking 25 h to achieve a 176% yield of octylBpin but produced fewer side products as C_6_Me_6_ was not borylated.^29^ Further work showed that these precatalysts react to give Rh boryl species, which are the actual catalysts, including [RhCp*(H)(Bpin)_3_] and [RhCp*(H)_2_(Bpin)_2_].^40,41^ The success of the RhCp*-based system was demonstrated in the C–H borylation of methane,^42^ with RuCp* and Ir catalysts the only other successful catalysts for this challenging reaction.^39,42−44^ The identification and design of improved Rh catalysts have proven to be difficult because the ability to modify the RhCp* fragment is limited. For example, RhCp* with a carboxylate-tethered NHC was successful,^45,46^ but a Rh boratabenzene catalyst was shown to deactivate quickly.^47^ Very recently, highly active Ir catalysts for alkane borylation have been identified including [Ir(Bpin)_3_(mesitylene)] or [{Ir(μ-OMe)(cod)}_2_] with tetramethylphenanthroline,^48−50^ 2-methylphenanthroline,^51,52^ 2-(hydro-ditertbutylsilylmethyl)phenanthroline,^53^ and 2,2′-dipyridylarylmethane ligands.^54^

We recently identified that [Rh(indenyl)(NHC)] fragments, which could be generated from [Rh(Ind)(NHC)(alkene)] but not [Rh(Ind)(NHC)(CO)] precursors, were efficient catalysts for the C–H borylation of arenes. [Rh(Ind)(SIDipp)(COE)] was identified as the best catalyst for benzene borylation at 80 °C, superior to IMes, SIMes, and fluorenyl-tethered NHC analogues.^22,55^ The C–H borylation of alkanes was also observed, although isolated yields of octylBpin and decylBpin from the direct C–H borylation of octane and decane were low (7 and 18%, respectively). We had reasoned that the [Rh(η^3^-Ind)(NHC)] fragment is isoelectronic to the very successful [RhCp*] fragment,^56^ but the full principles underpinning the design of a Rh catalyst for C–H borylation are not clear. In this work, we have synthesized the analogous [RhCp(NHC)] fragment to explore whether ring-slippage/the indenyl effect is an essential part of this catalyst system, as well as describing our progress toward fluorenyl and tetrahydrofluorenyl analogues. The role of the NHC has also been explored with reaction time courses for arene borylation showing dramatic differences between complexes containing different NHC ligands.

## 2 Results and Discussion

### 2.1 Synthesis of Cp Rh Complexes

**Scheme 2:**
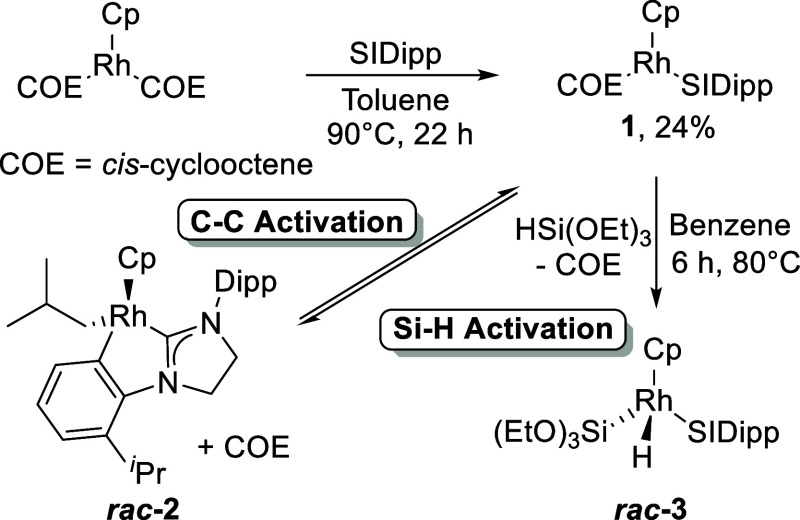
Synthesis, C–C Activation, and Oxidative Addition Reactivity of **1** SIDipp = 1,3-bis(2,6-diisopropylphenyl)-4,5-dihydroimidazol-2-ylidene.

[RhCp(COE)_2_] was synthesized as described in the literature.^57^ Although we observed that [RhCp*(C_2_H_4_)_2_] did not react with SIDipp to produce the desired complex (C–H activation of ethylene was observed instead),^55^ [RhCp(COE)_2_] did react with SIDipp at 90 °C under elimination of COE to produce the desired complex [RhCp(SIDipp)(COE)] (**1**, Scheme 2). Monitoring of the reaction by ^1^H NMR spectroscopy showed that a balance of factors was required; time and high temperature to facilitate the ligand substitution reaction with loss of COE but not excessive temperatures or reaction times as a side reaction was observed leading to an additional Rh complex (*rac***-2**, see below). These conditions are broadly similar to the synthesis of [Rh(Ind)(SIDipp)(COE)] which required 80 °C and 16 h reaction time,^55^ casting doubt that there is a significant acceleration of ligand substitution through ring-slippage in the indenyl complex. Purification by recrystallization resulted in the isolation of pure **1** in moderate yield (24%), evidenced by elemental analysis that showed all the expected resonances by ^1^H and ^13^C NMR spectroscopy. The Cp ^1^H and ^13^C{^1^H} resonances are doublets (0.6 and 3.4 Hz, respectively) due to coupling to ^103^Rh. The carbenic C resonance is also a doublet (215.4 ppm, 67.3 Hz) and similar to that observed in [Rh(Ind)(SIDipp)(COE)] (214.9 ppm, 70.1 Hz).^55^ Other resonances for the bound COE and SIDipp ligands are as expected.

**Figure 1:**
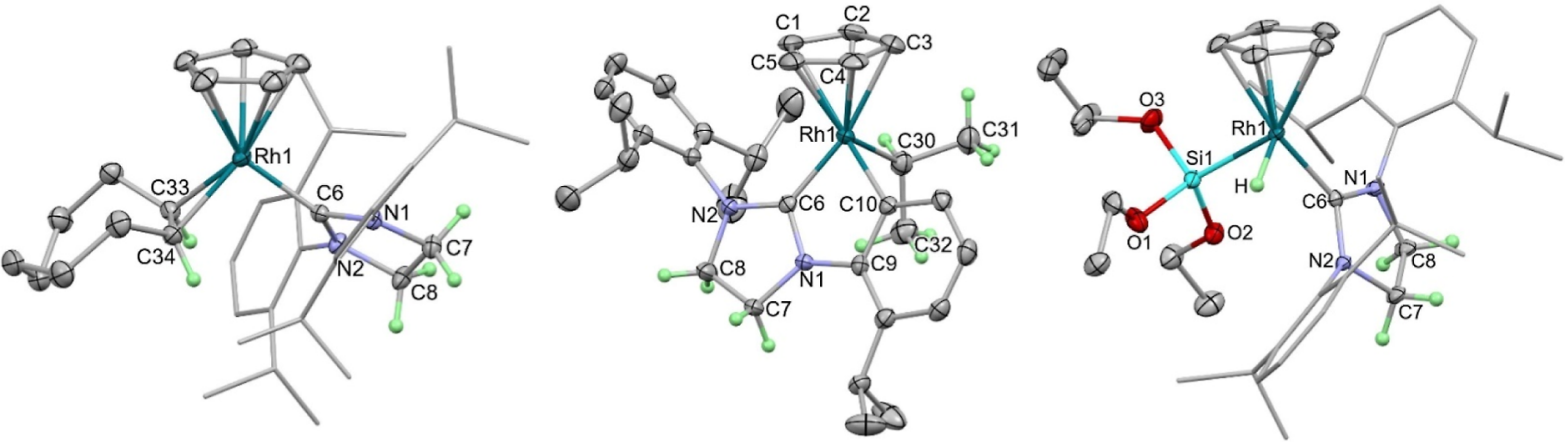
Molecular structures of **1** (left), *rac***-2** (middle), and *rac***-3** (right). Thermal ellipsoids are at 50% probability (Dipp substituents in **1** and *rac***-3** are displayed as capped sticks for clarity), and most H atoms are not displayed to enhance clarity. See the Supporting Information for additional data.

The molecular structure of **1** was determined from a single crystal grown from *n*-hexane and revealed a “two-legged” piano stool geometry (Figure 1), with the η^5^-Cp bound to Rh in a slightly uneven fashion [Rh–C_Cp_: 2.238(2)–2.333(2) Å]. The carbene Rh–C bond length is 1.994(2) Å, the Rh–C_COE_ bond lengths are 2.123(2), and the COE C=C bond length is 1.419(3) Å, which are similar to those in [Rh(Ind)(SIDipp)(COE)].^55^

From the filtrate, left over after the recrystallization and stored at −25 °C, several colorless crystals were observed indicative of Rh(III) with this ligand set, and the molecular structure of C–C activated *rac***-2** was determined by single crystal X-ray diffraction greatly facilitating the identification of the byproduct formed in the synthesis of **1**. Rh is bound to an η^5^-Cp ligand [Rh–C_Cp_: 2.267(2) - 2.320(2) Å] with a shorter Rh–C_carbene_ bond length of 1.939(2) Å compared to **1**. The NHC has been cyclometalated through oxidative addition of an isopropyl group, and a Rh–C_arene_ bond [2.004(2) Å] and Rh-^*i*^Pr group [Rh–C: 2.150(2) Å] are now present.

The C–C activation of a transition metal NHC complex was first described by Whittlesey, Macgregor, and co-workers in the thermolysis of [Ru(H)_2_(IMes)_2_(CO)(PPh_3_)] at 110 °C, which resulted in C–C bond cleavage, cyclometalation of the IMes ligand, and loss of methane.^58,59^ Subsequently, an osmium-amido IDipp complex was also found to undergo C–C activation and loss of propane.^60^*rac***-2** is unique in that both parts of the NHC are retained in the coordination sphere of the metal in the product. The activation of C–C bonds is perhaps even more challenging than C–H bonds and remains a major challenge in organometallic chemistry.^61−65^ C–N bond cleavage of NHC ligands has also been observed through loss of ^*t*^Bu^66^ or ^*i*^Pr^67^ N-substituents pointing toward different ligand activation routes depending on the presence of *N*-aryl or *N*-alkyl substituents.^66−69^ A catalytic arylation of quinoline has been developed that combines these routes utilizing both C–C and C–N bond cleavage of the imidazolium salt [IMesH]Cl or IMes, along with other imidazolium salts with different aryl substituents.^69^

The thermal C–C activation of **1** could not be driven to completion and gave an equilibrium ratio of 2.67:1 **1**:*rac***-2**, as demonstrated by heating C_6_D_6_ solutions of **1** at 80 °C (see Figure S4 in the Supporting Information). The ^1^H NMR spectrum of *rac***-2** is complex, with multiple doublets from the isopropyl groups and overlapping multiplets from isopropyl methines and the inequivalent methylenes on the carbene backbone. However, unique doublet-of-doublet (0.756 ppm) and doublet-of-triplet (7.804 ppm) resonances are now evident. Removal of the displaced COE under vacuum and continued heating in C_6_D_6_ in an attempt to force the equilibrium to favor the products still did not lead to the complete consumption of **1**, but additional Rh hydride complexes were now observed in small quantities as three doublets of different integrals: −14.14 ppm (35.8 Hz), −14.76 ppm (33.5 Hz), and −14.78 ppm (33.5 Hz; see Figure S7). These values are very similar to the hydride resonance in *rac***-3** (−14.35 ppm, 33.3 Hz) and are tentatively assigned to C–H cyclometalation of a Dipp group in analogy to the indenyl Rh analogue.^55^ These observations establish the high reactivity of compound **1** after dissociation of the COE ligand. This was confirmed by the oxidative addition of HSi(OEt)_3_^70^ to give the Rh silyl hydride *rac***-3**. A higher temperature (80 °C) was required compared to the analogous reaction with [Rh(Ind)(SIDipp)(COE)] (40 °C).^55^ Single crystals of *rac***-3** were analyzed by X-ray diffraction experiments to reveal the expected piano stool complex. Rh is bound to an η^5^-Cp ligand [Rh–C_Cp_: 2.226(2)–2.339(2) Å] with a Rh–C_carbene_ bond length of 1.985(2) Å, in an almost identical fashion to **1**. The Rh–Si bond length [2.2758(4) Å] is very similar to the indenyl analogue [2.2691(8) Å].^55^ The Rh–H was located, its position freely refined, and its presence was also confirmed by IR spectroscopy (ν_Rh–H_: 2100 cm^–1^).^70^

### 2.2 Synthesis of Indenyl, Fluorenyl, and Tetrahydrofluorenyl Rh NHC Complexes

**Scheme 3:**
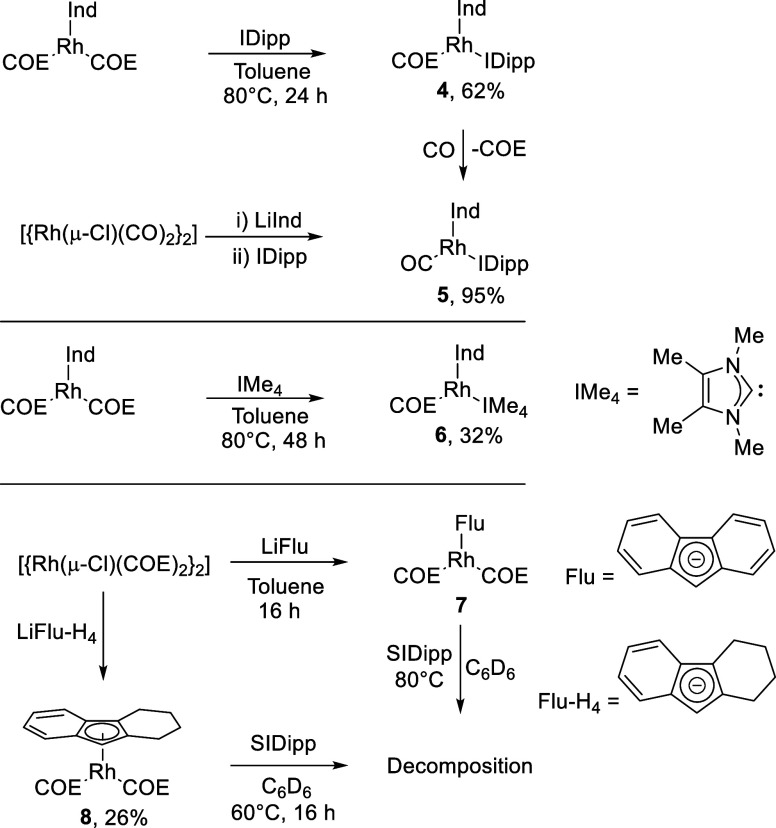
Synthesis of Indenyl (Ind), Fluorenyl (Flu), and 1,2,3,4-Tetrahydrofluorenyl (Flu-H_4_) Rh Complexes

**Figure 2:**
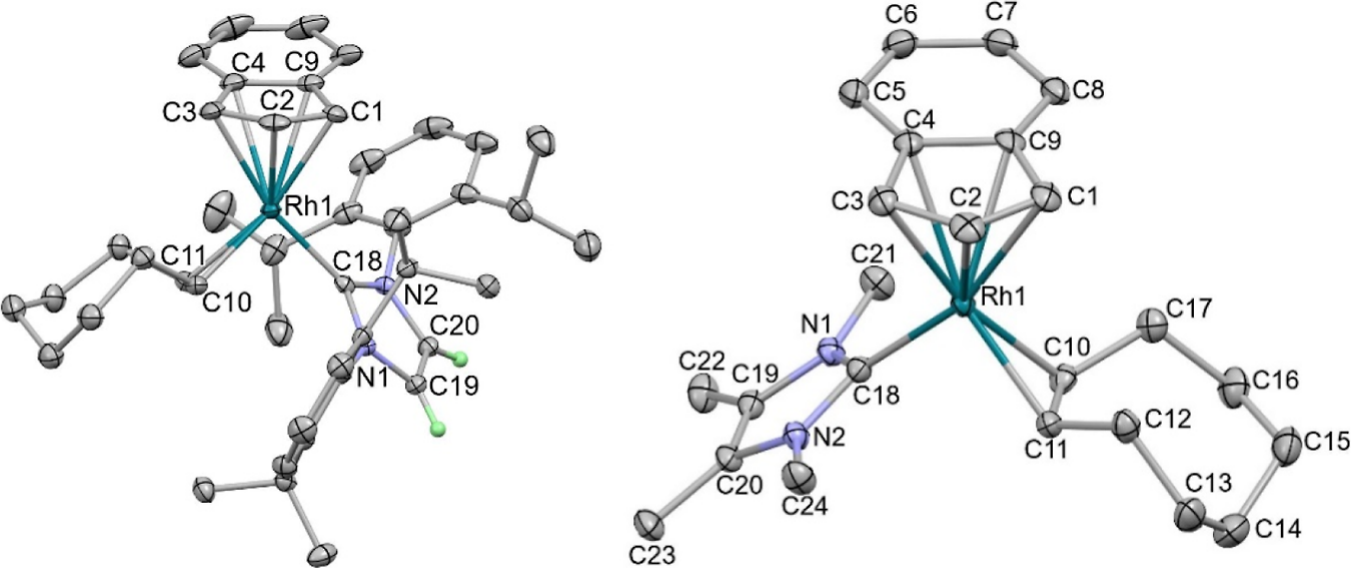
Molecular structure of **4** (left) and **6** (right). All H atoms except those on the NHC backbone have been removed for clarity; thermal ellipsoids at 50% probability.

Additional indenyl rhodium NHC complexes were synthesized to compare saturated and unsaturated Dipp-substituted NHCs, as well as smaller steric profiles (IMe_4_) and exchanging the indenyl ligand for fluorenyl derivatives. [Rh(Ind)(IDipp)(COE)] (**4**) was synthesized from the reaction of IDipp with [Rh(Ind)(COE)_2_] at 80 °C for 24 h, in an analogous manner to [Rh(Ind)(SIDipp)(COE)] (Scheme 3), and recrystallized from pentane in 62% yield; purity was confirmed by elemental analysis. ^1^H and ^13^C{^1^H} NMR spectroscopy revealed the expected resonances including the carbenic carbon as a doublet at 186.1 ppm with ^1^*J*_Rh–C_ coupling of 73.7 Hz. Similarly, a resonance was observed for the alkene of COE at 60.2 ppm with a ^1^*J*_Rh–C_ coupling of 15.5 Hz. The unsaturated NHC backbone appears as a singlet at 6.33 ppm. Single crystals of **4** were grown from a 1:5 mixture of toluene/pentane, and X-ray diffraction studies revealed a “two-legged” piano stool geometry (Figure 2). The indenyl is bound to rhodium in a distorted η^5^-interaction with three shorter bond lengths to C1, C2, and C3 and two longer bond lengths to C4 and C9 observed in both molecules in the asymmetric unit and similar to [Rh(Ind)(SIDipp)(COE)]. The rhodium-NHC bond lengths were 2.018(2) and 2.015(2) Å, which is slightly longer than in [Rh(Ind)(SIDipp)(COE)] [1.988(2) and 1.995(2) Å; two molecules in the asymmetric unit],^71^ but the alkene bond length was indistinguishable at 1.410(2) Å.

The carbonyl analogue of this complex was synthesized by the addition of carbon monoxide to **4** leading to the clean conversion to [Rh(Ind)(IDipp)(CO)] (**5**) in quantitative yields (Scheme 3). **5** could also be synthesized in a second synthetic procedure on small scales from the addition of LiInd to [{Rh(μ-Cl)(CO)_2_}_2_], then by the subsequent addition of IDipp to the reaction mixture to yield **5**. The carbonyl ^13^C NMR signal was observed at 195.1 ppm with a ^1^*J*_Rh–C_ coupling of 92.3 Hz. The carbenic carbon was visible at 186.9 ppm with a ^1^*J*_Rh–C_ coupling of 72.5 Hz. Similar resonances were present for the SIDipp analogue [Rh(Ind)(SIDipp)(CO)].^55^ High-resolution mass spectrometry revealed the expected mass for [**5** + H]^**+**^ and SCXRD showed the expected “two-legged” piano stool geometry with a rhodium carbene bond length of 2.019(2) Å (see Supporting Information for further details). IR spectroscopy revealed a C≡O stretching frequency for **5** of 1940 cm^–1^, which is lower than that for the saturated SIDipp analogue (1944 cm^–1^)^55^ suggesting that the carbonyl in **5** is bound to a slightly more electron rich metal center.

[Rh(Ind)(IMe_4_)(COE)] (**6**) was synthesized to probe the effect of the smaller size of IMe_4_ because it should be more resistant to C–H cyclometalation due to the unfavored formation of a 4-membered ring that would result. **6** was synthesized by the addition of IMe_4_ to [Rh(Ind)(COE)_2_] at 80 °C for 48 h (Scheme 3). Purification through Celite gave **6** in a modest yield of 32%. The expected resonances for this complex were observed in the ^1^H and ^13^C{^1^H} NMR spectra. In the ^13^C{^1^H} NMR spectrum, the carbene resonance was observed at 181.1 ppm with a ^1^*J*_Rh–C_ coupling of 66.9 Hz, and the Rh-COE signal was apparent at 56.9 ppm with a ^1^*J*_Rh–C_ coupling of 16.6 Hz. Two signals of the bound indenyl ligand couple to rhodium at 96.1 and 70.9 ppm with ^1^*J*_Rh–C_ couplings of 5.4 and 4.1 Hz, respectively. HRMS of **6** revealed the [M + H]^+^ ion, which was unusual as all other [Rh(Cp/Ind)(NHC)(COE)] complexes showed loss of COE. Single crystals of **6** were grown from a concentrated toluene solution at −20 °C. SCXRD studies revealed the “two-legged” piano stool geometry of this species (Figure 2) with a rhodium–carbene bond length of 1.999(2) Å; this is similar within error to [Rh(Ind)(SIDipp)(COE)]. The indenyl is bound to the metal center through an η^5^-interaction [Rh–C: 2.233(2)–2.389(2) Å] with less distortion than in **4**. The COE ligand was bound to rhodium through an η^2^-interaction, and the C=C bond length was identical within error to both **4** and [Rh(Ind)(SIDipp)(COE)]; however, the Rh–C_alkene_ bond lengths for **6** [2.090(2) and 2.114(2) Å] were shorter than those for both **4** [2.117(2) and 2.152(2) Å] and [Rh(Ind)(SIDipp)(COE)] [2.116(2)/2.138(2) and 2.140(2)/2.137(2) Å]; therefore, COE is bound closer but in a similar “slipped” fashion.

With the success of synthesizing [Rh(Ind)(NHC)(COE)] complexes, fluorenyl analogues were seen as viable targets. This would also allow for a direct comparison with the previously synthesized [Rh(Flu-NHC)(COE)]-tethered systems,^55^ helping to elucidate whether the fluorenyl donor or presence of a linker hindered arene C–H borylation catalysis. Working on small scales (up to 20 mg performed in J. Young NMR tubes) suggested that the precursor complex [Rh(Flu)(COE)_2_] (**7**) could be synthesized from [{Rh(μ-Cl)(COE)_2_}_2_] and LiFlu with the reaction going to completion. The expected signals were observed by using both ^1^H NMR and ^13^C{^1^H} NMR spectroscopy. However, when reactions were performed on 100 mg scale, they did not go to completion and instead led to a product-to-fluorene ratio of 56:44; Rh(0) precipitate was also observed which could be removed by Celite filtration. However, samples could not be purified further and addition of SIDipp did not produce the desired product.

The work of Kharitonov et al. drew our attention to the 1,2,3,4-tetrahydrofluorenyl ligand (Flu-H_4_), an indenyl-type ligand with a steric profile similar to fluorenyl, and the synthesis of [Rh(Flu-H_4_)(COD)] and [Rh(Flu-H_4_)(C_2_H_4_)_2_] was reported.^72^ We synthesized the COE analogue (**8**) by deprotonation of tetrahydrofluorene using *n*-BuLi in THF followed by reaction with half an equivalent of [{Rh(μ-Cl)(COE)_2_}_2_] yielding **8** as a viscous oil in 26% yield after Celite filtration. Single crystals of **8** suitable for SCXRD grew from the concentrated oil over time but revealed a highly disordered structure with the Flu-H_4_ ligand disordered across three positions, with additional disorder also present in the COE ligands as well (see Supporting Information). The geometry is a “two legged” piano stool complex with the tetrahydrofluorenyl ligand bound to the metal center through an η^5^-interaction (the central C atoms bound to the metal center are not disordered), and the two COE ligands are bound with η^2^-interactions. The [Rh(Flu-H_4_)(COD)] complex was similarly disordered over two positions.^72^ Unfortunately, the addition of SIDipp to **8** at 60 °C led to the near complete decomposition of **8;** however, a small carbene signal was observed by ^13^C{^1^H} NMR spectroscopy at 211.2 ppm with a ^1^*J*_Rh–C_ coupling of 75.1 Hz in agreement with the expected product [Rh(Flu-H_4_)(SIDipp)(COE)]. However, the complex could not be isolated, which leads to the tentative conclusion that it is the steric profile of fluorenyl and Flu-H_4_ ligands that led to difficulties when synthesizing NHC complexes.

### 2.3 Arene C–H Borylation

**Scheme 4:**
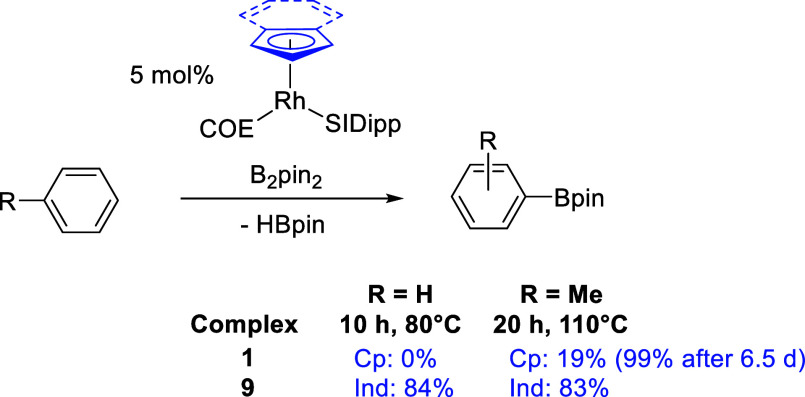
Arene Borylation Comparing Cp and Indenyl Complexes

The borylation of benzene was carried out at 80 °C using 5 mol % of both the indenyl ([Rh(Ind)(SIDipp)(COE)], **9**) and cyclopentadienyl ([RhCp(SIDipp)(COE)], **1**) precatalysts (Scheme 4). Strikingly, no reaction occurred using the Cp complex, whereas an 84% yield of PhBpin was observed after 10 h for the indenyl complex, increasing to 97% after 24 h.^55^ For the Cp complex, ^1^H NMR spectroscopy showed resonances that matched those of the C–C bond activation product *rac***-2** appearing over the course of 10 h. For comparison, [RhCp*(C_2_H_4_)_2_] was tested as a catalyst, but it was poor giving only a 10% yield of PhBpin and 35% consumption of B_2_pin_2_ after 24 h at 80 °C. This is in-line with literature precedent as when [RhCp*(C_2_H_4_)_2_] was tested as a catalyst for benzene borylation, a temperature of 150 °C was used.^29,36^

A similar result was observed for the borylation of toluene at 110 °C. The Cp complex **1** gave a yield of 19% tolylBpin (combined yield of ortho, meta, and para isomers) after 20 h whereas the indenyl catalyst **9** was much faster yielding 83% tolylBpin. It was noteworthy that at this higher temperature, there is now an accessible pathway for C–H borylation mediated by the Cp complex, albeit one that is more difficult than for the indenyl complex. Monitoring the reaction further for the Cp complex showed that catalysis continued reaching a 99% yield of tolylBpin after 6.5 d, and even further conversion of HBpin into tolylBpin was also observed at longer time periods (see Supporting Information). This indicates that although the catalyst derived from **1** is slow, it is relatively stable with its long lifetime in solution at high temperatures, allowing good conversions to be reached after long reaction times. Interestingly, the C–C activation product *rac***-2** was again observed from reaction times of 15 min to 3 h, but a different and more symmetrical Rh complex featuring a hydride ligand was observed from 20 h (−14.87 ppm, 33.2 Hz; see Figure S31), indicating that other Rh complexes are accessible from *rac***-2** or that *rac***-2** is in equilibrium with the COE complex **1** and that the C–C activation product is not merely a “dead-end”.

**Scheme 5:**
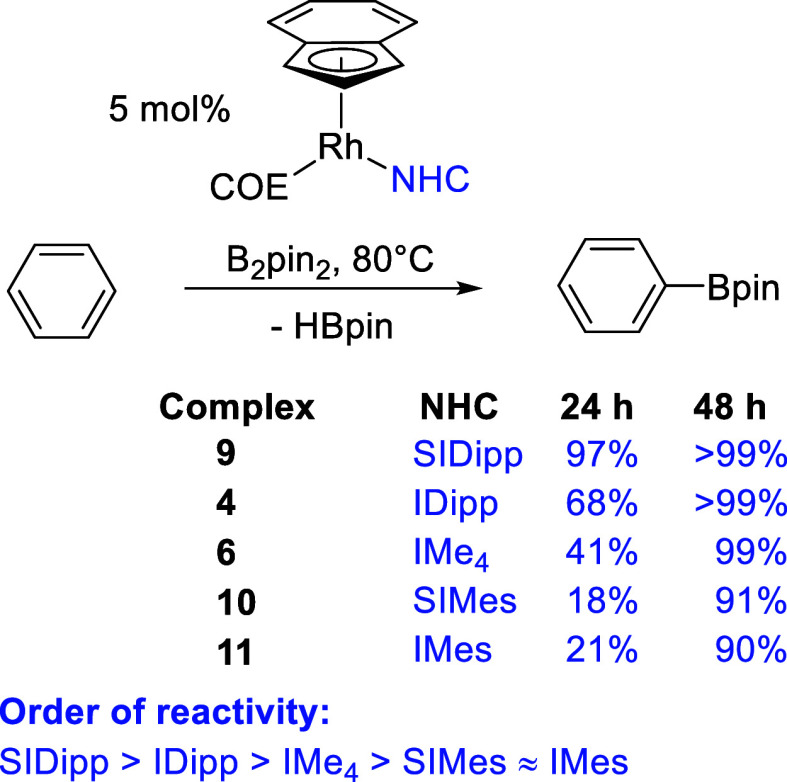
Catalytic Benzene C–H Borylation Comparing Different NHC Ligands

[Rh(Ind)(IDipp)(COE)] (**4**) and [Rh(Ind)(IMe_4_)(COE)] (**6**) were also tested as catalysts for benzene C–H borylation, but both were less active than [Rh(Ind)(SIDipp)(COE)] (**9**) (Scheme 5). After 24 h at 80 °C, the IDipp complex **4** gave 68% PhBpin, compared to 97% for the saturated SIDipp analogue; previously, SIMes (**10**) and IMes (**11**) complexes were found to be very similar to each other giving ca. 18 and 21% PhBpin after 24 h, respectively, increasing to 91 and 90% after 48 h.^55^ The IMe_4_ complex **6** was intermediate, producing 41% PhBpin after 24 h. However, both **4** and **6** gave quantitative yields after 48 h.

**Figure 3:**
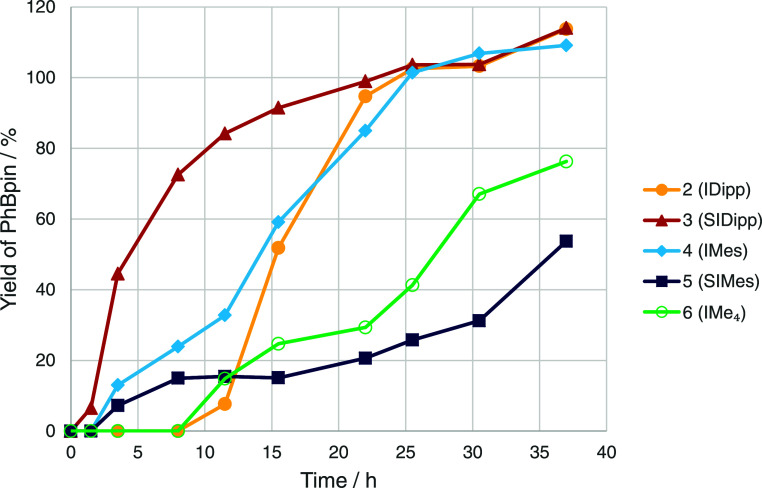
Reaction time courses for [Rh(Ind)(NHC)(COE)] catalysts in the C–H borylation of benzene at 80 °C, monitoring the formation of PhBpin using ^1^H NMR spectroscopy. Lines are added to guide the eye.

We sought more detailed information about the course of these reactions, particularly as induction periods are likely to play an important role, given the yields observed after 24 and 48 h for several of these catalysts, most notably with SIMes and IMes ligands. Using ^1^H NMR spectroscopy and ferrocene as an internal standard, reaction time courses for **4**, **6**, and **9** – **11** were constructed for the C–H borylation of benzene monitoring loss of B_2_pin_2_ and production of PhBpin and HBpin (see Supporting Information for individual profiles). Looking at the production of PhBpin (Figure 3) for **9**, an induction period of about 1 h was observed; however, for the IDipp analogue **4**, a much longer induction period of about 10 h was observed. The IMe_4_ complex **6** showed a similar induction period, followed by a rate of reaction that varied with time in a complicated fashion. SIMes and IMes complexes **10** and **11** showed shorter induction periods of ca. 2 h, but again complicated reaction kinetics were observed, with IMes now recording a higher yield after 24 h than that seen previously. Clearly, the NHC ligand has a significant impact on the formation and nature of the catalyst in C–H borylation. Monitoring the loss of B_2_pin_2_, an induction period was most obvious only for **4**. For the production of HBpin that accompanies this reaction, we would expect to see the same concentration as PhBpin if these catalysts do not consume HBpin to produce an extra equivalent of PhBpin or other byproducts. What was observed was that the concentrations of PhBpin and HBpin were very similar until yields of between 40 and 60%, and then the concentration of HBpin began to decrease, eventually reaching zero. However, the extra equivalent of PhBpin that would be expected was not observed, and, instead, only a small amount of HBpin was converted into additional PhBpin. **6** is the exception to this generalization with HBpin matching PhBpin production up to almost 80% and eventually yielding 140% PhBpin after 100 h with 30% HBpin remaining. It therefore appears that this catalyst—although slower—gives rise to fewer side reactions.

HBpin (76 mol %) was added to the catalytic borylation of benzene with B_2_pin_2_ (100 mol %) using **4** (5 mol %) to see if immediate, irreversible hydroboration of the COE ligand would eliminate the induction period. This was indeed successful with PhBpin now observed from 15 min, and the reaction reached high conversions within 4 h (see Figure S39).

### 2.4 Alkane C–H Borylation

**Scheme 6:**
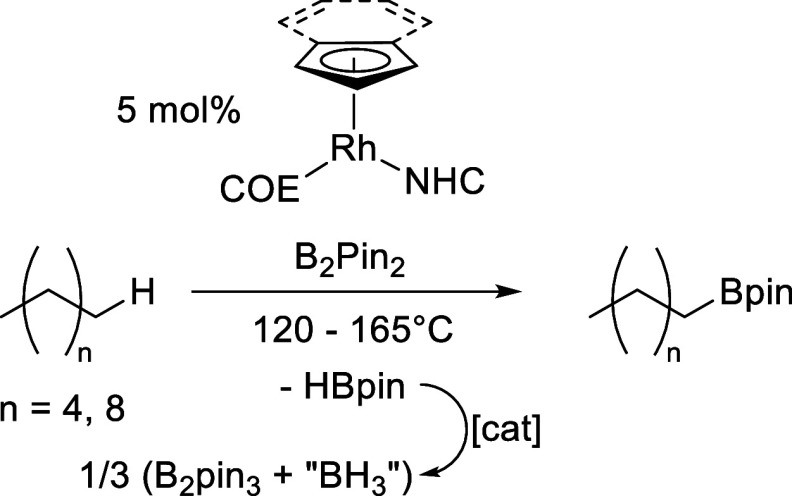
Catalytic Alkane C–H Borylation

The more challenging alkane C–H borylation reaction was then targeted for these catalysts. *n*-Decane (bp = 174 °C) was selected so that reactions could be run at temperatures up to 150 °C without superheating the solvent so that conventional glassware and regular sampling could be carried out, whereas *n*-hexane (bp = 69 °C) was selected to test catalysis in an autoclave suitable for more volatile substrates (Scheme 6).

Literature results had demonstrated that monitoring the reaction in sealed NMR tubes using ^11^B{^1^H} NMR spectroscopy is possible,^54^ but, for us, the considerable overlap between broad resonances as well as the formation of an additional product at 21.65 ppm (tentatively assigned as B_2_pin_3_ from HBpin decomposition)^51,73^ and the associated problem of not being able to identify the fate of all BH fragments meant that only qualitative results could be achieved. *para*-Carborane was used as an internal standard to verify that both B_2_pin_2_ and HBpin are thermally stable at 140 °C; however, *para*-carborane is not inert to borylation in the presence of either [RhCp(SIDipp)(COE)] or [Rh(Ind)(SIDipp)(COE)] producing *para*-B_10_C_2_H_11_Bpin, as shown by mass spectrometry and ^11^B NMR spectroscopy. This led to the use of GC-FID analysis to evaluate reaction progress using calibration curves derived from authentic samples of hexylBpin and decylBpin synthesized from alkene hydroboration and purified by column chromatography followed by Kugelrohr distillation (see Supporting Information).

For the borylation of *n*-decane (Table 1), it is clear that rapid catalyst deactivation is a problem with which we have to contend for this catalyst design. At 140 °C, 5 mol % **9** gave 18% yield of decylBpin in 1 h, rising only slightly to 21% after 24 h. This is broadly in line with the isolated yields reported previously for *n*-octane and *n*-decane (5 mol % **9** with *n*-octane at 130 °C: 7% octylBpin; 10 mol % **9** with *n*-decane at 150 °C: 18% decylBpin), demonstrating relatively efficient workup and isolation of the alkylBpin products.^55^ At 120 °C, conversion of B_2_pin_2_ was slower and yields of decylBpin were no better, reaching 10% after 1 h and 14% after 24 h. **4** proved to be very similar in performance to **9**. **2**, featuring a Cp ligand, was much poorer, giving only 1% yield of decylBpin after 1 h and 6% after 24 h, mimicking the results for arene borylation. Comparisons to [RhCp*(C_2_H_4_)_2_] revealed the superiority of this precatalyst, which gave 82% decylBpin after 24 h.

**Table 1:** Collated Catalytic Run Data for the Borylation of *n*-Decane by Various Catalysts Using B_2_pin_2_^a^

entry	catalyst	temp (°C)	time (h)	B_2_pin_2_ conversion (%)	decylBpin yield (%)
1	[Rh(Ind)(SIDipp)(COE)] (**9**)	120	1	59	10
2			2	72	12
3			3	85	13
4			4	96	13
5			24	100	**14**
6	[Rh(Ind)(SIDipp)(COE)] (**9**)	140	1	79	18
7			2	92	18
8			3	99	18
9			4	100	19
10			24	100	**21**
11	[Rh(Ind)(IDipp)(COE)] (**4**)	140	1	54	11
12			2	98	17
13			3	100	18
14			4	100	18
15			24	100	**19**
16	[Rh(Cp)(SIDipp)(COE)] (**2**)	140	1	16	1
17			2	22	1
18			3	33	1
19			4	44	1
20			24	100	**6**
21	[RhCp*(C_2_H_4_)_2_]	140	1	80	47
22			2	96	73
23			3	99	76
24			4	99	76
25			5	100	**82**

a 3 mL of *n*-decane, 40 mg of B_2_pin_2_, catalyst loading: 5 mol %. Yields and conversions for the formation of one equivalent of decylBpin from B_2_pin_2_ determined by GC-FID using a calibration curve based on authentic samples are an average of two runs except for [RhCp*(C_2_H_4_)_2_] (one run).

The borylation of *n*-hexane in an autoclave was carried out, but yields were decreased compared with *n*-decane (Table 2). **9** and **4** were similar, giving 10% yield of hexylBpin after 2 h at 165 °C, and heating for longer time periods did not lead to yields better than 12%, again pointing to catalyst deactivation. Mesityl-substituted **11** was a poorer precatalyst (5% yield of hexylBpin). Comparing Cp*, indenyl, and Cp ligands bound to [Rh(alkene)_2_] fragments revealed the superiority of Cp* (31% yield of hexylBpin after 2 h) followed by indenyl (10%) then Cp (3%). For reactions with these and the NHC-ligated catalysts, the conversion of B_2_pin_2_ was high and often quantitative, but the fate of all the Bpin fragments could not be determined. Conversely, using [{Rh(μ-Cl)(COE)_2_}_2_], or adding no Rh catalyst, gave no yield of hexylBpin and ≤12% conversion of B_2_pin_2_.

**Table 2:** Collated Catalytic Run Data for the Borylation of *n*-Hexane by Various Catalysts and B_2_pin_2_ (Yields Are Based on the Consumption of B_2_pin_2_ Only)^a^

entry	catalyst	time (h)	B_2_pin_2_ conversion (%)	hexylBpin yield (%)
1	[Rh(Ind)(SIDipp)(COE)] (**9**)	2	76	10
2	[Rh(Ind)(IDipp)(COE)] (**4**)	2	99	9
3	[Rh(Ind)(IMes)(COE)] (**11**)	2	88	5
4	[RhCp*(C_2_H_4_)_2_]	2	99	31
5	[Rh(Ind)(COE)_2_]	2	90	10
6^b^	[RhCp(COE)_2_]	2	100	3
7	[RhCl(COE)_2_]_2_	2	12	<1
8	No catalyst	2	10	<1
9	[Rh(Ind)(SIDipp)(COE)] (**9**)	4	100	12
10	[Rh(Ind)(IDipp)(COE)] (**4**)	4	100	11
11	[Rh(Ind)(SIDipp)(COE)] (**9**)	6	98	11
12	[Rh(Ind)(SIDipp)(COE)] (**9**)	20	98	9

a B_2_pin_2_ (121 mg, 0.476 mmol), Rh catalyst (5 mol %, 0.024 mmol), and *n*-hexane (15 mL); temperature across all reactions averaged 165 °C. Yield was based on the consumption of B_2_pin_2_ to give one equiv of hexylBpin with conversion/yield determined by GC-FID using a calibration curve.

b Reduced volume of hexane (6 mL) used.

### 2.5 Mechanistic Insights

**Scheme 7:**
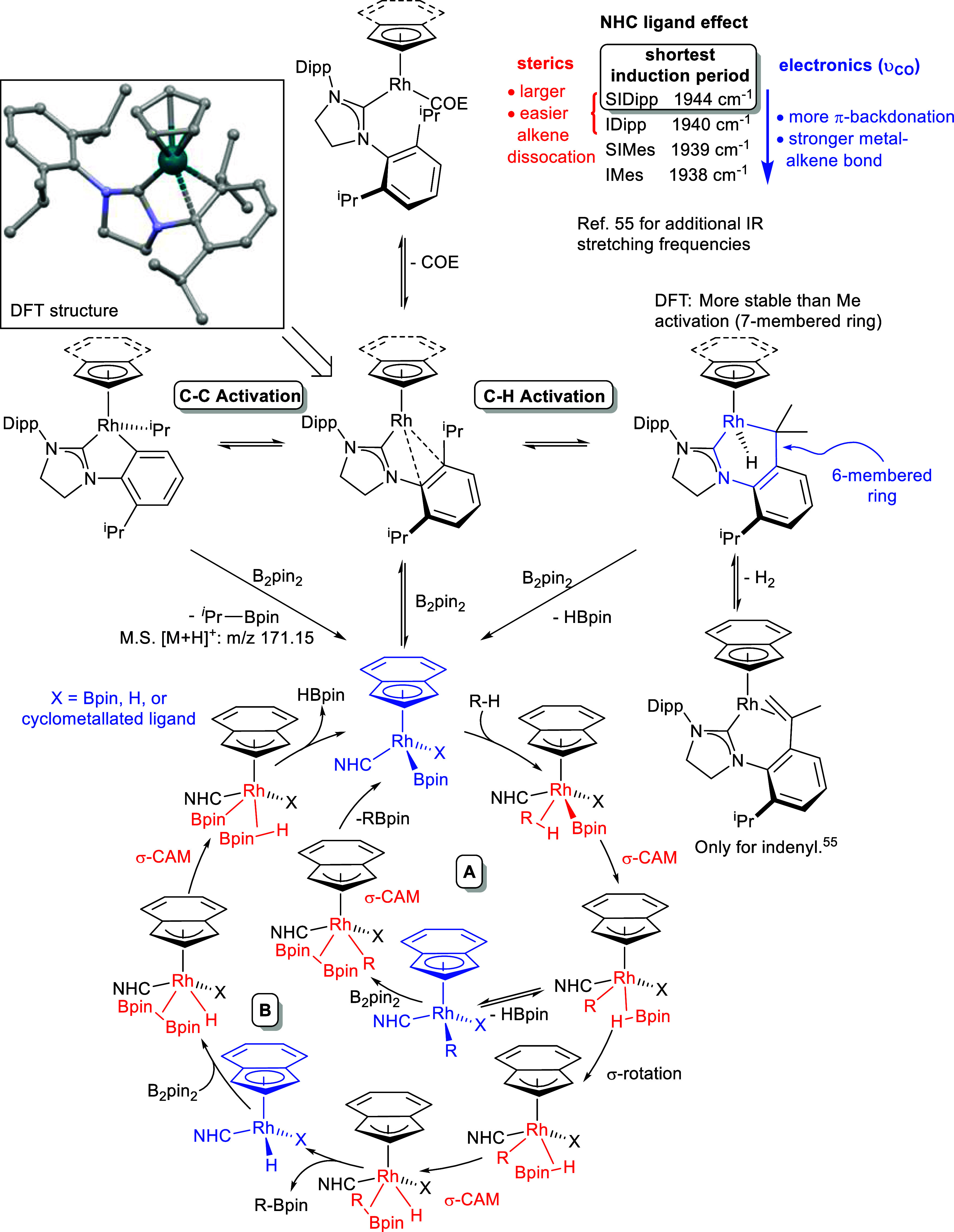
Proposed Catalytic Cycle with Loss of HBpin Followed by RBpin (Pathway **A**) or Vice Versa (Pathway **B**)

With these data in hand, a representation of the catalytic cycle can be postulated (Scheme 7). There is extensive evidence of the importance of the NHC to the catalysis, impacting both induction time and rate of the reaction, implying that the NHC remains bound in the catalytic cycle. The importance of the indenyl ligand to the catalysis has also been demonstrated as dramatic differences between Cp, indenyl, and Cp* were seen. As previous work in the literature established that [Ir(η^5^-Ind)(COD)] reacts with HBcat (COD = 1,4-cyclooctadiene; cat = 1,2-O_2_C_6_H_4_) to generate [Ir(Bcat)_3_(η^6^-arene)],^74^ the potential for loss of indenyl in B_2_pin_2_-mediated C–H borylation reactions needed to be assessed. GC–MS (in selected ion monitoring mode) was used to analyze an *n*-decane C–H borylation reaction with **9** as the catalyst, and indene, indane, and Bpin-substituted analogues (C_15_H_19_BO_2_, C_15_H_21_BO_2_, and C_21_H_32_B_2_O_4_) were observed as seen analogously in the Ir reaction above.^74^ However, these species could be generated either in pathways leading to the catalytically active species or from catalyst decomposition. COE dissociates in the initiation step, as was observed by ^1^H NMR spectroscopic investigations of reactions with these precatalysts. Interestingly, SIDipp, along with IDipp, was the most sterically bulky ligand and gave the best catalyst with the shortest induction period potentially by favoring the dissociation of COE. It was also the least electron donating ligand in the [Rh(Ind)(NHC)(CO)] derivatives, indicating that the Rh center is less electron rich and therefore that COE will more readily dissociate in the COE derivatives due to less metallacyclopropane character, according to the Dewar–Chatt–Duncanson model.^75^ Mass spectrometry of reaction mixtures revealed cyclooctylBpin to be present, not cyclooctenylBpin or diborylated cyclooctane, leading to the conclusion that cyclooctene is hydroborated by HBpin that is produced in C–H borylation in a different process.

**Figure 4:**
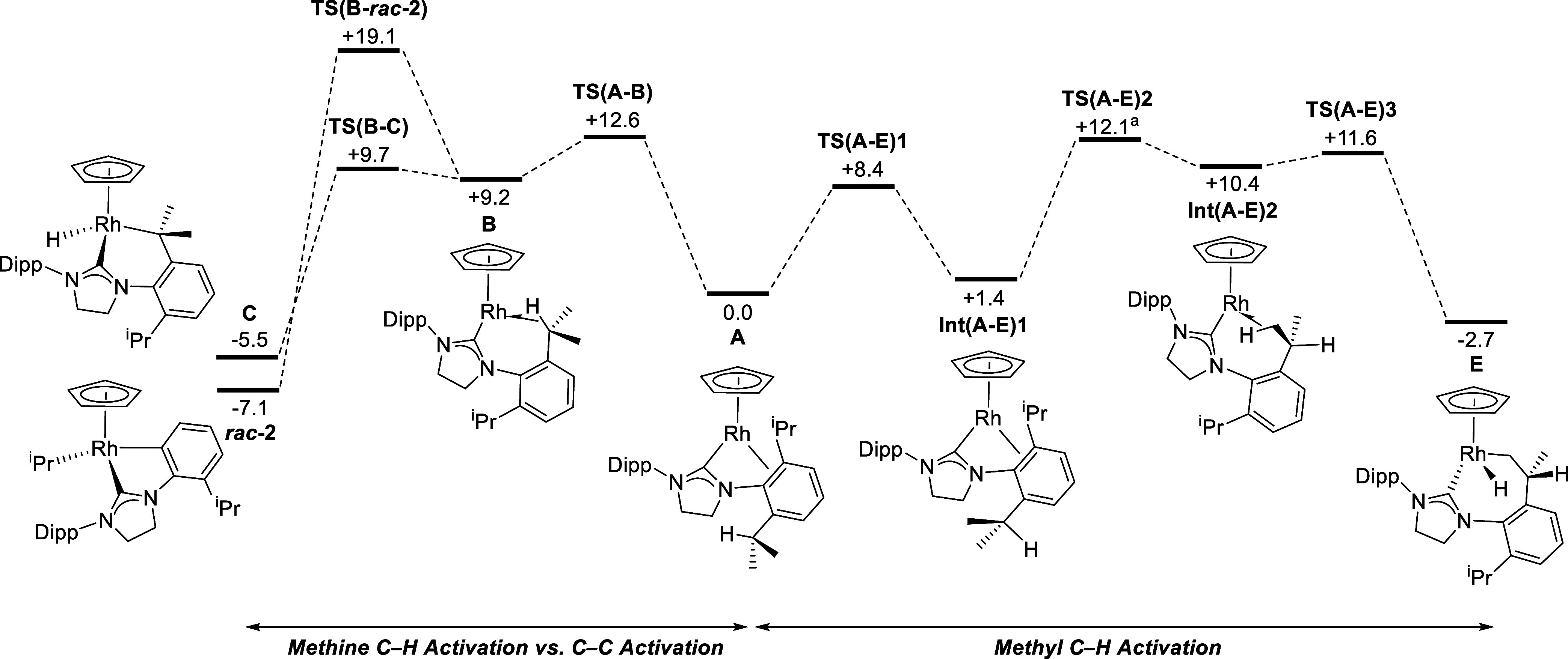
Computed free energy reaction profiles (kcal/mol) for competing C–H and C–C bond activation in [Rh(Cp)(SIDipp)], A. Method: BP86(def2-tzvp, BJD3, C_6_H_6_)//BP86(Rh: SDD; other atoms: 6-31G**). ^a^**TS(A-E)2** leads initially to a different C–H agostic isomer of **Int(A-E)2**, but the two readily interconvert (see Supporting Information for full details).

DFT calculations were employed in order to shed some light on the behavior of the 16 electron [Rh(Cp)(SIDipp)] and [Rh(Ind)(SIDipp)] intermediates formed via COE dissociation from **1** and **9,** respectively. Figure 4 shows the computed reaction profiles for C–H and C–C activation in the Cp system that forms the well-defined C–C activated product *rac***-2** experimentally. Similar results were obtained for the indenyl system (see Supporting Information for full details).

Figure 4 shows that the most stable form of [Rh(Cp)(SIDipp)], **A**, involves an η^2^-interaction with one C_ipso_–C_ortho_ bond in a Dipp substituent (molecular structure shown in Scheme 7). A similar motif has been reported in the [W(Cp)(CO)_2_(IMes)]^+^ cation by Bullock and co-workers,^76^ and both this and the computed structure of **A** exhibit significant “yawing”^77^ of the NHC ligand; for **A,** the relevant Rh–C_NHC_–N angles are 148.5°/103.4° and the interacting C_ipso_–C_ortho_ bond lengthens to 1.47 Å (cf. 1.42 Å for the noninteracting Dipp C_ipso_–C_ortho_ bonds). Similar asymmetries have also been reported in otherwise formally unsaturated related group 9 NHC derivatives.^78,79^ Despite the distortion, this η^2^-bound form is considerably more stable than alternative C–H agostic intermediates (see below).

Both C–C and methine C–H activation of a Dipp substituent can be accessed through a common methine C–H agostic intermediate, **B** (+9.2 kcal/mol), formed via **TS(A-B)** at +12.6 kcal/mol. From here, C–H activation gives cyclometalated **C** at −5.5 kcal/mol via **TS(B-C)** at +9.7 kcal/mol; C–C activation entails a higher transition state, **TS(B**-*rac***-2)**, at +19.1 kcal/mol, to form *rac***-2** at −7.1 kcal/mol. The alternative methyl C–H bond activation in **A** proceeds via initial rotation of an isopropyl substituent to form **Int(A-E)1**. From here, a methyl–C–H agostic intermediate **Int(A-E)2** can be accessed from which C–H activation proceeds with a minimal barrier to give **E** at −2.7 kcal/mol.

Overall, both methine and methyl C–H activation processes are kinetically accessible with low overall barriers of 12.6 and 12.1 kcal/mol, respectively. Methine C–H activation is favored thermodynamically (**C** at −5.5 kcal/mol, cf. **E** at −2.7 kcal/mol^80^) likely due to preferential formation of a 6-membered metallacycle over a 7-membered metallacycle. Nonetheless, both processes would be reversible, allowing access to the thermodynamically more stable C–C activated *rac***-2** at −7.1 kcal/mol. The return barrier for C–C coupling is 26.2 kcal/mol, consistent with this process being reversible at the temperatures used for catalysis.

After loss of COE, access to a Rh-boryl complex is possible via oxidative addition of B_2_pin_2_ to [Rh(Ind)(NHC)] or by reaction with Rh-alkyl/aryl species^81^ generated through cyclometallation producing a bis(boryl) or mono boryl complex, respectively. The complexity of the onward catalytic reaction (indenyl hapticity and rotation; multiple isomers with NHC, Bpin, aryl/alkyl, and hydride ligands; σ-complex intermediates; the potential for oxidative addition/reductive elimination or σ-CAM pathways; the presence of cyclometalated intermediates, etc.) meant that further computational investigation of the catalytic cycle has not been possible so far, but an analogous cycle to the [RhCp*] system is possible if indenyl binds in an η^3^ binding mode (Scheme 7). A σ-CAM pathway only requires the presence of one boryl ligand, with σ-borane complexes playing a crucial part,^82,83^ and is perhaps more likely than oxidative addition to Rh^3+^ as it avoids Rh^5+^. The proposed catalytic cycle shows two possibilities with either loss of HBpin first (pathway A) or loss of RBpin first (pathway B). Finally, there is the potential for different cycles to be important for benzene borylation at 80 °C or alkane borylation at 140 °C, and it should be noted that the Rh–NHC catalysts were observed to give very similar reaction rates for the higher temperature reactions.

Additional insights into the mechanism are possible from an in situ analysis of the catalytic reactions. Unfortunately, monitoring catalytic borylation reactions involving **9** using ^1^H NMR spectroscopy did not reveal distinct NMR signals, indicating that a mixture of complexes had formed instead of a single resting state or intermediate. However, as mentioned above, in the borylation of toluene using **1** at 110 °C, *rac***-2** was observed first, which then evolved into a symmetrical Rh complex featuring a hydride ligand, in agreement with [RhCp(H)(X)(SIDipp)] where X could be Bpin or aryl. The catalytic borylation of benzene using **4**, B_2_pin_2_, and HBpin also revealed a Rh–H resonance, in agreement with [Rh(Ind)(H)(X)(IDipp)] as an intermediate. Other evidence for the reaction pathways proposed in Scheme 7 includes the observation of [^*i*^PrBpin + H]^+^ by mass spectrometry (Figure S47), showing that B_2_pin_2_ reacts with Rh-^*i*^Pr complexes to form RBPin.

## 3 Conclusions

This research has demonstrated that the [Rh(Ind)(SIDipp)(COE)] precatalyst is superior to the cyclopentadienyl analogue for arene and alkane C–H borylation and superior to [RhCp*(C_2_H_4_)_2_] for the C–H borylation of benzene at 80 °C. SIDipp was the best NHC ligand for Rh-catalyzed arene C–H borylation most obviously due to a much shorter induction period than the smaller mesityl-substituted NHCs SIMes and IMes, as well as the unsaturated derivative IDipp. Thus, there is both a steric and electronic benefit from using the SIDipp ligand. The smaller IMe_4_ carbene gave intermediate performance between Dipp- and Mes-substituted complexes. [RhCp*(C_2_H_4_)_2_] proved to be the best catalyst for *n*-hexane and *n*-decane borylation and superior to [Rh(Ind)(NHC)(COE)] catalysts, which suffered from significant catalyst deactivation within the first hour of the reaction. [RhCp(SIDipp)(COE)] was again observed to be a poorer catalyst than the indenyl analogue, demonstrating the clear superiority of the indenyl ligand in catalytic C–H borylation. For alkane borylation at high temperatures (120–165 °C), there was much less of a difference in yields observed between different NHC ligands.

## 4 General Procedure for Borylation Reactions

Full experimental details and the general experimental description are available in the Supporting Information. General procedures are given below:

### 4.1 NMR-Scale Reactions of the Borylation of Benzene and Toluene

In a glovebox, the Rh complex (3.5 μmol, 5 mol %), ferrocene (internal standard, 1.4 mg, 7.5 μmol), and B_2_pin_2_ (17.7 mg, 70 μmol) were combined in the arene (3:1 protio/deuterio arene, 0.7 mL) and added to an NMR tube equipped with a J. Young valve. The sample was then heated in an oil bath at 80 °C (benzene) or 110 °C (toluene), and the reaction was monitored using ^1^H and ^11^B NMR spectroscopy.

### 4.2 NMR-Scale Reactions of the Borylation of *n*-Decane

In a glovebox, the Rh complex (3.7 μmol,5 mol %) and B_2_pin_2_ (19 mg, 75 μmol) were combined in *n*-decane (0.7 mL) and sealed in an NMR tube equipped with a J. Young valve. The sample was then heated in an oil bath at 140 °C, and the reaction was monitored using ^11^B NMR spectroscopy.

### 4.3 Larger-Scale *n*-Decane Borylations Monitored by GC-FID

In a glovebox, the Rh complex (7.9 μmol, 5 mol %) and B_2_pin_2_ (40 mg, 158 μmol) were combined in *n*-decane (3 mL) and sealed in a glass vessel equipped with a J. Young valve. The sample was then heated at 140 °C and the reaction monitored by GC-FID using 0.1 mL aliquots extracted from the vessel periodically under a positive pressure of nitrogen.

### 4.4 Borylation of *n*-Hexane

In a glovebox, B_2_pin_2_ (121 mg, 0.476 mmol) and the Rh catalyst (5 mol %, 0.024 mmol) were added to a 450 mL Parr autoclave. Dry *n*-hexane (15 mL, 114 mmol) was then placed in the autoclave under a flow of nitrogen on a Schlenk line. The autoclave was sealed and placed in a preheated (150 °C) Al heating block. The internal thermocouple was monitored until it reached 150 °C and then stirring commenced at 500 rpm. The temperature displayed on the internal thermocouple was found to rise to approximately 165 °C. The autoclave was cooled using an ice bath, and a sample of the product solution was passed through filter paper. The crude filtered sample was analyzed by GC-FID, and the yield was obtained using a calibrated dodecane internal standard.

## Supporting Information Available

Full experimental details including synthesis and thermal stability of the catalysts , comprehensive product characterization including NMR, MS, and XRD data, catalysis results, and details of the computational investigations including computational references and computed reaction profiles (PDF)
Structures of all the computationally modeled structures (XYZ)

## Supplementary Material

om4c00025_si_001.pdf

om4c00025_si_002.xyz
