# Supplementary material for: Catalyst Design for Rh-Catalyzed Arene and Alkane C–H Borylation: The NHC Affects the Induction Period, and Indenyl is Superior to Cp
Source: Organometallics. 2024 Mar 28;43(9):974–86. doi: 10.1021/acs.organomet.4c00025 (PMC11094794; doi:10.1021/acs.organomet.4c00025)
Supplement: Supplementary file 1 — om4c00025_si_001.pdf [file om4c00025_si_001.pdf]

Catalyst design for Rh-catalysed arene and alkane C-H borylation: The NHC affects the induction period and indenyl is superior to Cp

Paul A. Morton, Abigayle L. Boyce, Anamarija Pišpek, Lennox W. Stewart, Daniel J. Ward, Bengt E. Tegner,[a] Stuart A. Macgregor[b,]* and Stephen M. Mansell*

Institute of Chemical Sciences, Heriot-Watt University, Edinburgh, EH14 4AS, UK.

[a] Current address: Department of Chemistry, University of Liverpool, Liverpool L69 3BX, UK.

[b] Current address: EaSTCHEM School of Chemistry, North Haugh, University of St. Andrews, St. Andrews, KY16 9ST

E-mail: s.a.macgregor@hw.ac.uk; s.mansell@hw.ac.uk, https://www.mansellresearch.org.uk

1 Table of Contents

2 Experimental Details and NMR spectra ... 2

2.1 General details ... 2

2.2 Synthesis of [RhCp(SIDipp)(COE)] (**1**) ... 3

2.3 Thermal stability of [RhCp(SIDipp)(COE)] (**1**) ... 5

2.4 Thermal stability of [Rh(Ind)(SIDipp)(COE)] ... 8

2.5 Synthesis of [RhCp{Si(OEt)$_3$}(H)(SIDipp)] (***rac*-3**) ... 9

2.6 Synthetic routes to [Rh(Ind)(NHC)(alkene)] ... 10

2.6.1 [Rh(Ind)(IDipp)(COE)] (**4**) ... 10

2.6.2 [Rh(Ind)(IDipp)(CO)] (**5**) ... 11

2.6.3 [Rh(Ind)(IMe$_4$)(COE)] (**6**) ... 13

2.7 Synthesis of [Rh(Flu)(COE)$_2$] (**7**) ... 14

2.8 Synthesis of [Rh(Flu-H$_4$)(COE)$_2$] (**8**) ... 15

2.9 General procedure for borylation reactions ... 17

2.9.1 Synthesis and characterisation of the alkylBpin GC standards from hydroboration reactions ... 17

2.10 ^{1}H NMR spectroscopic monitoring of arene borylation reactions ... 21

2.10.1 Benzene C-H borylation ... 21

2.10.2 Toluene borylation ... 22

2.10.3 Reaction profiles for benzene C-H borylation at 80°C ... 25

2.10.4 Decane borylation: NMR spectroscopic monitoring ... 29

2.10.5 Control reactions ... 32

2.11 Mass spectrometry from C-H borylation reactions ... 33

3 X-ray crystallography ... 36

3.1 Crystallographic details ... 36

3.2 Structures of additional monodentate complexes ... 36

3.2.1 [Rh(Ind)(IDipp)(COE)] (**4**) ... 36

3.2.2 [Rh(Ind)(IDipp)(CO)] (**5**) 36
3.2.3 A new polymorph of [Rh(Ind)(SIMes)(COE)] 37
3.2.4 [Rh(Ind)(IMe_4)$_2$] (**6**) 37
3.2.5 [Rh(Flu-H_4)(COE)$_2$] (**8**) 37
3.3 Comparsion of bond lengths and angles for Rh complexes 38
3.4 Crystallographic tables of data 40
4 High resolution mass spectrometry data 43
4.1 Rh complexes 43
4.1.1 [Rh(Ind)(IDipp)(CO)] (**4**) 43
4.1.2 [Rh(Ind)(IMe_4)(COE)] (**6**) 44
4.1.3 [Rh(Flu-H_4)(COE)$_2$] (**8**) 45
5 Computational Details. 46
5.1 Computational References 46
5.2 Computed Reaction Profiles. 47
6 References 50

2 Experimental Details and NMR spectra

2.1 General details

All reactions requiring inert condition were performed under an oxygen free nitrogen atmosphere by using standard Schlenk line techniques or by using an MBRUAN UNILab Plus glovebox, unless otherwise noted. Dry toluene, CH_2Cl_2 and THF were obtained from a solvent purification system (MBraun SP-300) and stored over 4 Å molecular sieves prior to use. Benzene was dried over molten potassium and distilled, or dried over activated 4 Å molecular sieves, prior to use. Toluene-d_8 was freeze-pump-thaw degassed and dried over activated 4 Å molecular sieves prior to use. Non-dry solvents were used as received from Fisher Scientific. For the catalytic borylation reactions, benzene and toluene were dried as described above; n-octane and n-decane were dried over molecular sieves. n-Hexane and cyclohexane were purchased as a dry solvent over molecular sieves (Fisher). SIDipp,[1] SIMes,[1] IDipp,[1] IMes[2] and IMe_4[3] were synthesised according to literature methods. $RhCl_3$ hydrate was received from Johnson Matthey and used to make the following starting materials according to literature methods: [{Rh(μ-Cl)(COE)$_2$}$_2$],[4] [{Rh(μ-Cl)(C_2H_4)$_2$}$_2$],[5] [{Rh(μ-Cl(CO)$_2$}$_2$],[6] [Rh(Ind)(COE)$_2$],[7] [Rh(Cp)(COE)$_2$][8] and [Rh(Cp*)(C_2H_4)$_2$][9]. Tetrahydrofluorene was synthesised as previously described.[10] NMR spectra were obtained using either a AVIII400 (400 MHz) or AVIIIHD (400 MHz) spectrometer. ^{1}H NMR spectra were recorded at 400 MHz and referenced to the residual solvent peak (7.24 for $CDCl_3$, 2.09 for toluene-d_8, and 7.16 for C_6D_6). ^{13}C{^{1}H} NMR spectra were recorded at 101 MHz and referenced to the residual solvent peak (77.16 for $CDCl_3$ and 128.06 for C_6D_6). ^{11}B{^{1}H} NMR spectra were recorded at 128.4 MHz and referenced to an external sample of BF_3.OEt_2. FTIR was performed on a Thermo Scientific Nicolet iS5/iD5 ATR spectrometer. Mass spectrometry was conducted at the National Mass Spectrometry Facility at Swansea University using the techniques stated or using an in-house Shimadzu LCMS-2020 mass spectrometer in ASAP mode. Elemental analyses were performed at Heriot-Watt University (non air-sensitive), London Metropolitan University (air sensitive) and Elemental Microanalysis Ltd (Okehampton, air sensitive).

GC analysis used a Shimazdu GC-2014 with a DB-5 column (0.25 µm film thickness, 30 m length, 0.25 mm inner diameter). The GC method used an initial temperature of 55 °C with a hold time of 1 minute. This was followed by a ramp at 10 °C/min until 100 °C, followed by a ramp at 30 °C/min with a final temperature of 250 °C and a hold time of 5 minutes. Concentrations and yields of B_2pin_2 and the product alkylBpin were determined by calibrated GC-FID analysis using an internal standard (hexadecane).

2.2 Synthesis of [RhCp(SIDipp)(COE)] (1)

[Rh(Cp)(COE)$_2$] (179 mg, 0.461 mol), SIDipp (180 mg, 0.461 mol) and toluene (5 cm^3) were combined in a flask equipped with a J. Young cap in a glovebox. The flask was then taken out of the glovebox and stirred at 90 °C for 22 h. All of the volatiles were removed under vacuum giving an orange oil. This was completely dissolved in pentane (15 cm^3) and the solution was concentrated to ca. 5 cm^3 under reduced pressure and placed at 4°C for 16 h. The yellow crystalline material that was produced was isolated by filtration and dried under vacuum giving [RhCp(SIDipp)(COE)] (**1**) as a yellow-orange solid (82 mg, 0.113 mmol, 24 %).

^{1}H (400.1 MHz, 298 K, C_6D_6) δ /ppm: 7.22 (m, 2 H, Ar *p*-H), 7.14 (m, 4 H Ar *m*-H), 4.69 (d, $^{2}J_{Rh\text{-}H}$ = 0.6 Hz, 5H, Cp-H), 3.53 (apparent sept., $^{3}J_{H\text{-}H}$ = 6.9 Hz, C*H*Me$_2$), 3.34 (s, 4 H NC*H*$_2$), 2.22 (m, 2 H, C=CH), 2.13 (m, 2 H, COE CH$_2$), 1.73 (m, 2 H, COE CH$_2$), 1.53 (d, $^{3}J_{H\text{-}H}$ = 6.9 Hz and overlapping COE CH$_2$, 16 H), 1.33 (m, 4 H, COE CH$_2$), 1.11 (d, $^{3}J_{H\text{-}H}$ = 6.9 Hz, 12 H, Dipp Me);**^{13}C{^{1}H} (100.6 MHz, 298 K, C_6D_6) δ /ppm:** 215.38 (d, $^{1}J_{Rh\text{-}C}$ = 67.3 Hz, carbene), 147.25 (s, Ar 4°C), 141.21 (s, Ar 4° C), 128.89 (s, *p*-Ar CH), 124.89 (s, *m*-Ar C-H), 88.28 (d, $^{1}J_{Rh\text{-}H}$ = 3.4 Hz, Cp), 54.56 (d, $^{2}J_{Rh\text{-}C}$ = 1.5 Hz, NCH$_2$), 53.87 (d, $^{1}J_{Rh\text{-}C}$ = 16.8 Hz, *C*=*C*), 34.80 (s, COE CH$_2$), 33.33 (s, COE CH$_2$), 29.30 (s, Dipp CH), 27.40 (s, COE CH$_2$), 26.65 (s, Dipp Me), 24.08 (s, Dipp Me); **HRMS (ASAP/TOF)**: Calcd. for [$C_{32}H_{43}N_2{}^{103}Rh]^+$: 558.2, [M-COE]$^+$, Found: 558.2 m/z; **Elemental analysis:** Calcd (%) for $C_{40}H_{57}N_2Rh$: C 71.83, H 8.59, N 4.19; found C 71.67, H 8.73, N 4.07.

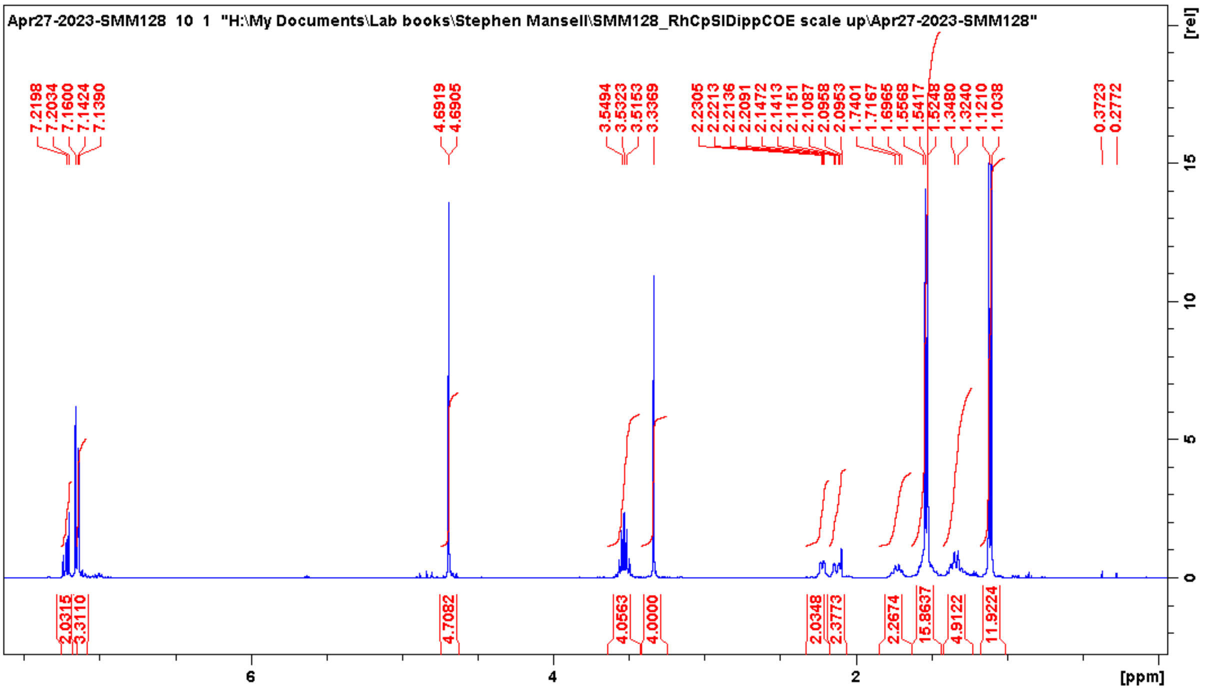

Figure S1. ^{1}H NMR spectrum (400 MHz, C_6D_6, 298 K) of [RhCp(SIDipp)(COE)] (**1**).

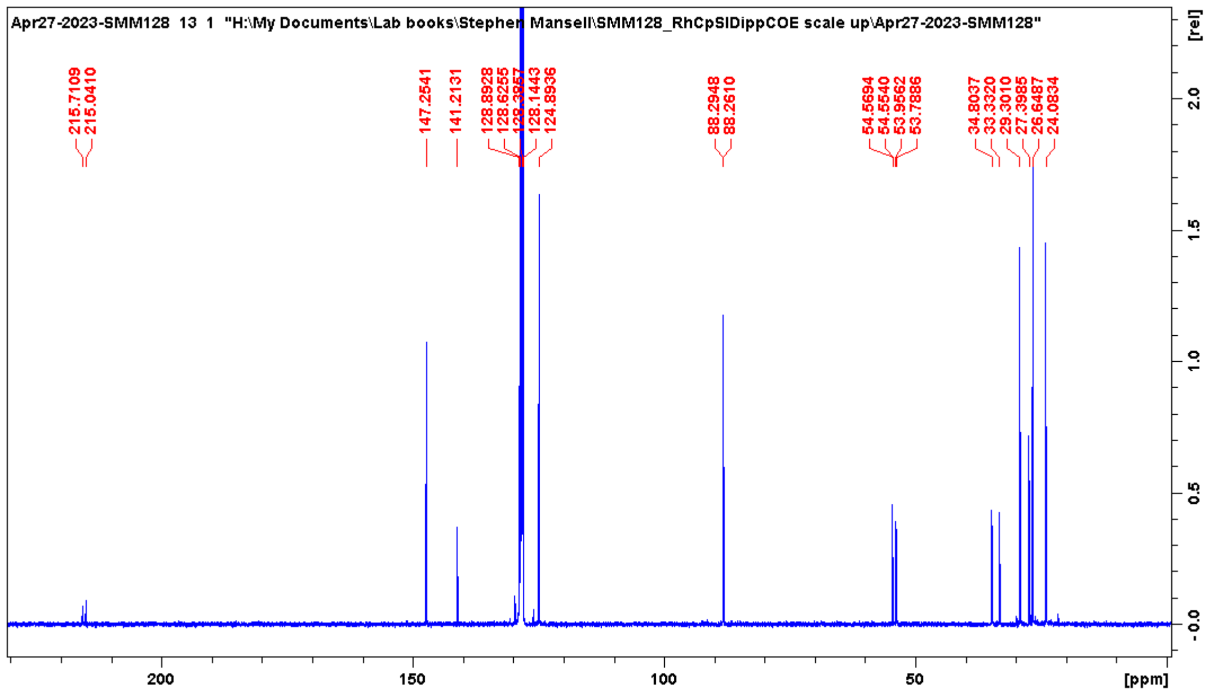

Figure S2. $^{13}C\{^{1}H\}$ NMR spectrum (101 MHz, C_6D_6, 298 K) of [RhCp(SIDipp)(COE)] (**1**).

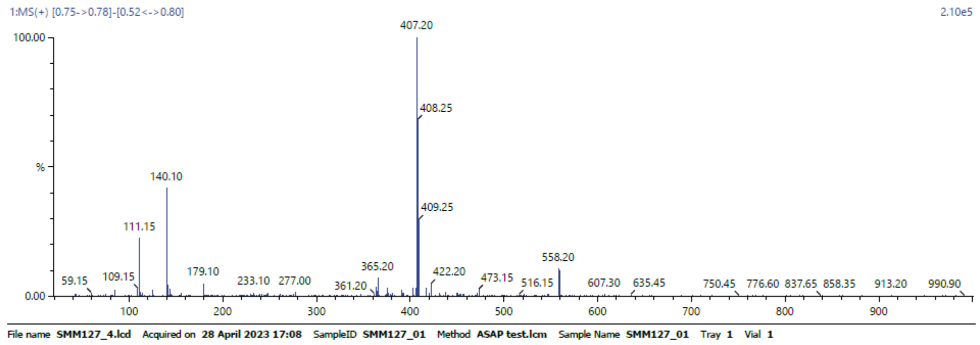

Figure S3. Mass spectrum of **1** for [M-COE]$^+$.

2.3 Thermal stability of [RhCp(SIDipp)(COE)] (1)

A solution of [RhCp(SIDipp)(COE)] (13 mg) in C_6D_6 (0.7 cm^3) was heated to 80°C and the reaction monitored by ^{1}H NMR spectroscopy.

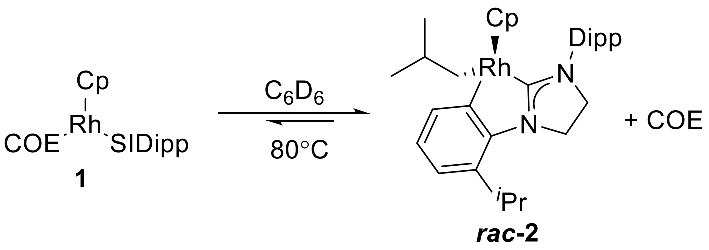

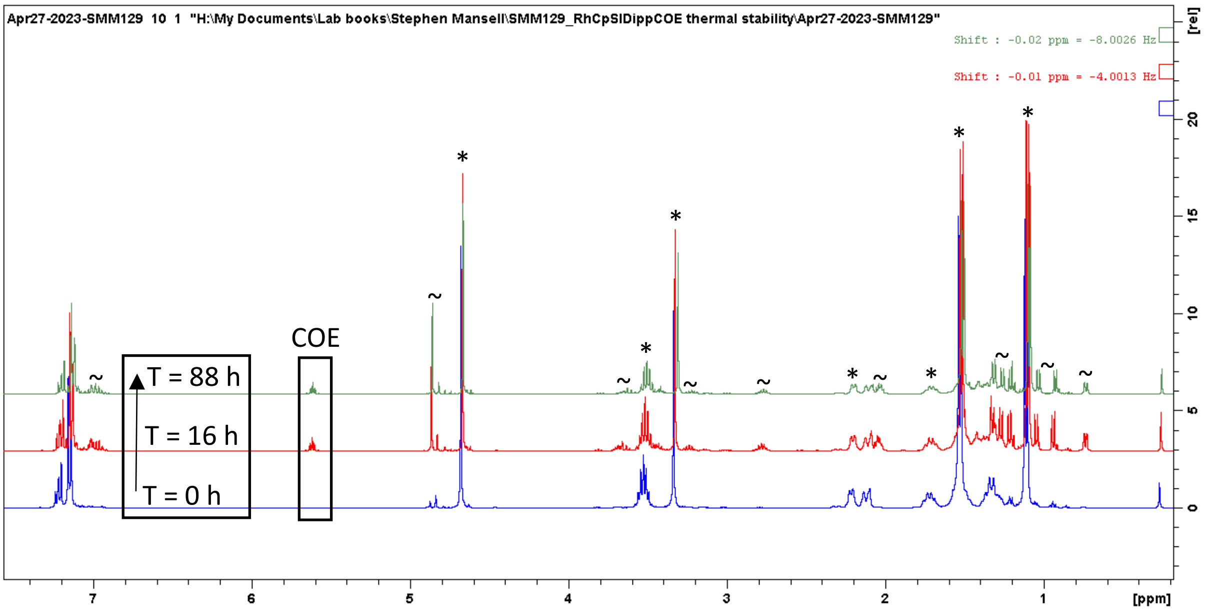

Figure S4. ^{1}H NMR spectra (400 MHz, C_6D_6, 298 K) of [RhCp(SIDipp)(COE)] after heating at 80°C. * indicates [RhCp(SIDipp)(COE)] starting material; ~ indicates resonances for the C-C activation product ***rac*-2**. After 16 h there was a 2.67:1 ratio of **1**:***rac*-2**, which remained the same after 88 h heating.

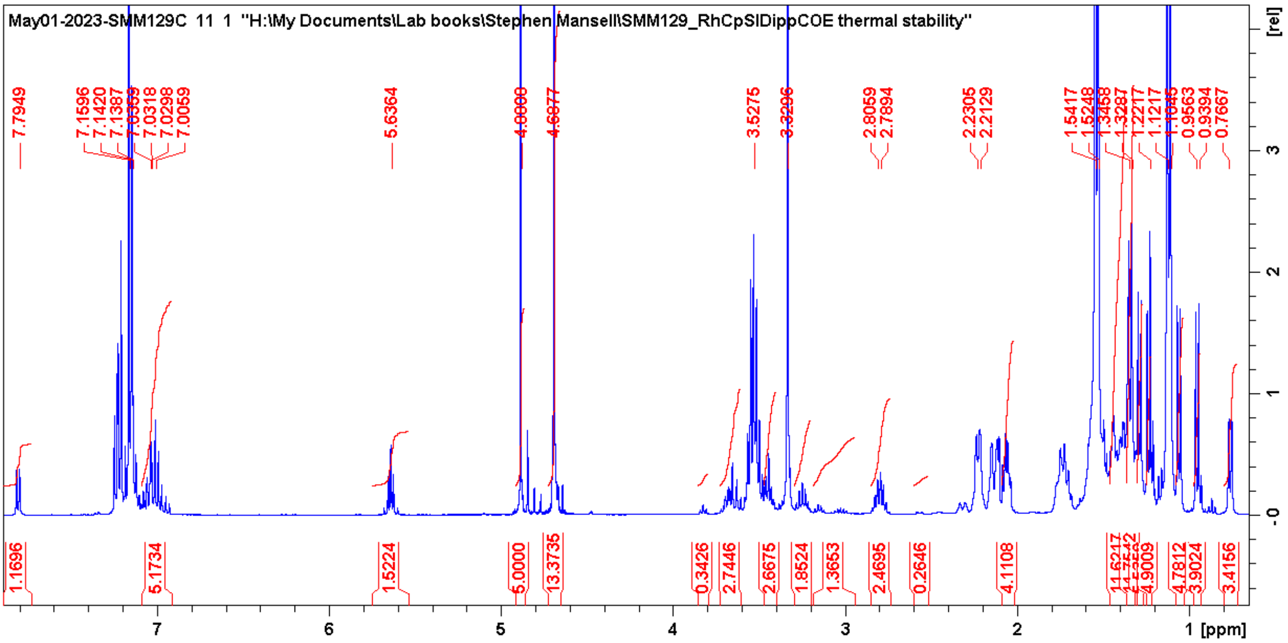

Figure S5. Integrated ^{1}H NMR spectrum (400 MHz, C_6D_6, 298 K) of **1** / ***rac*-2**.

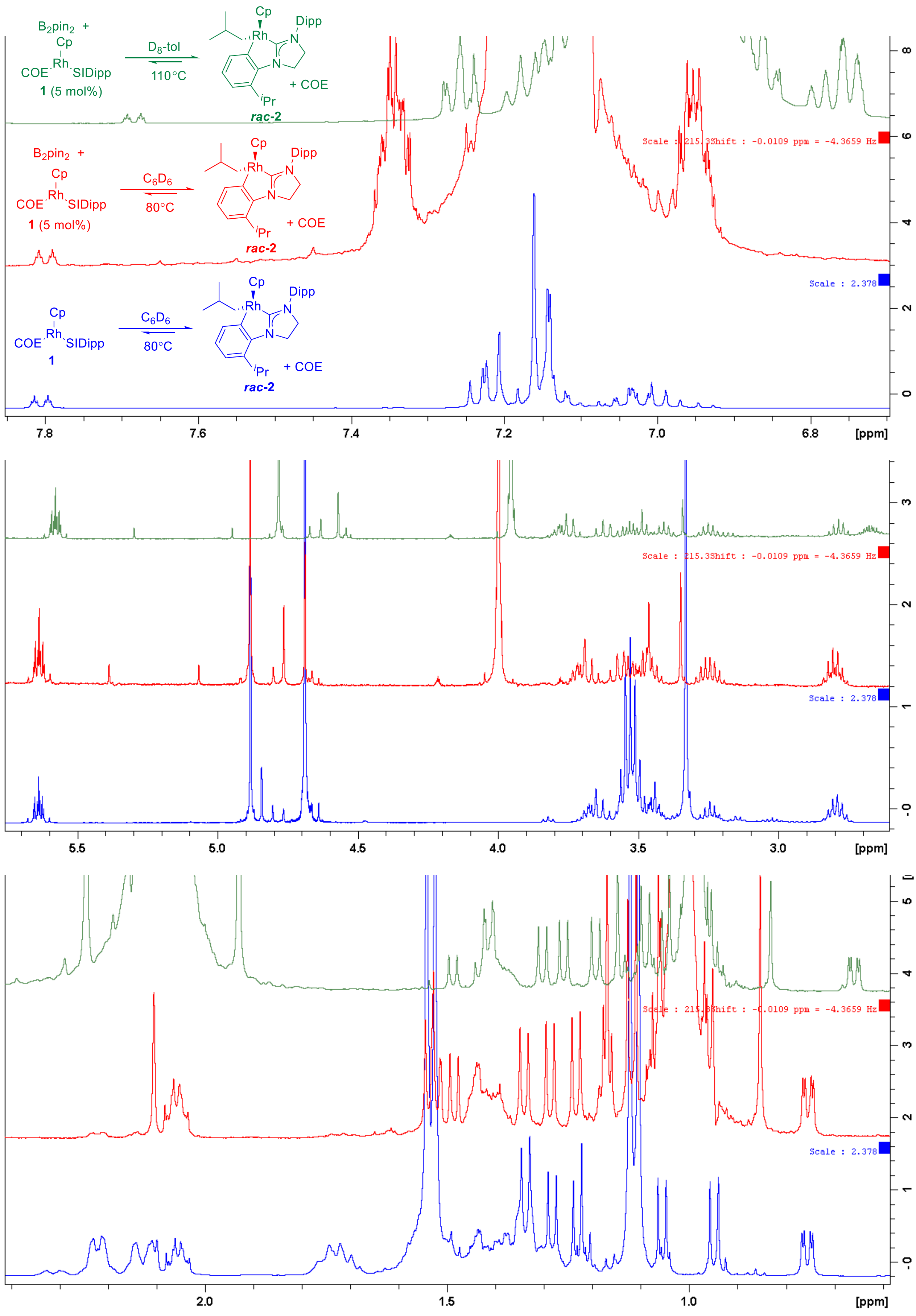

Figure S6. Comparison of ^{1}H NMR spectra (split into three different regions) of ***rac*-2** observed in heating **1** in C_6D_6 (blue), and from heating 5 mol% **1** / B_2pin_2 in C_6D_6 (red) and d_8-toluene (green).

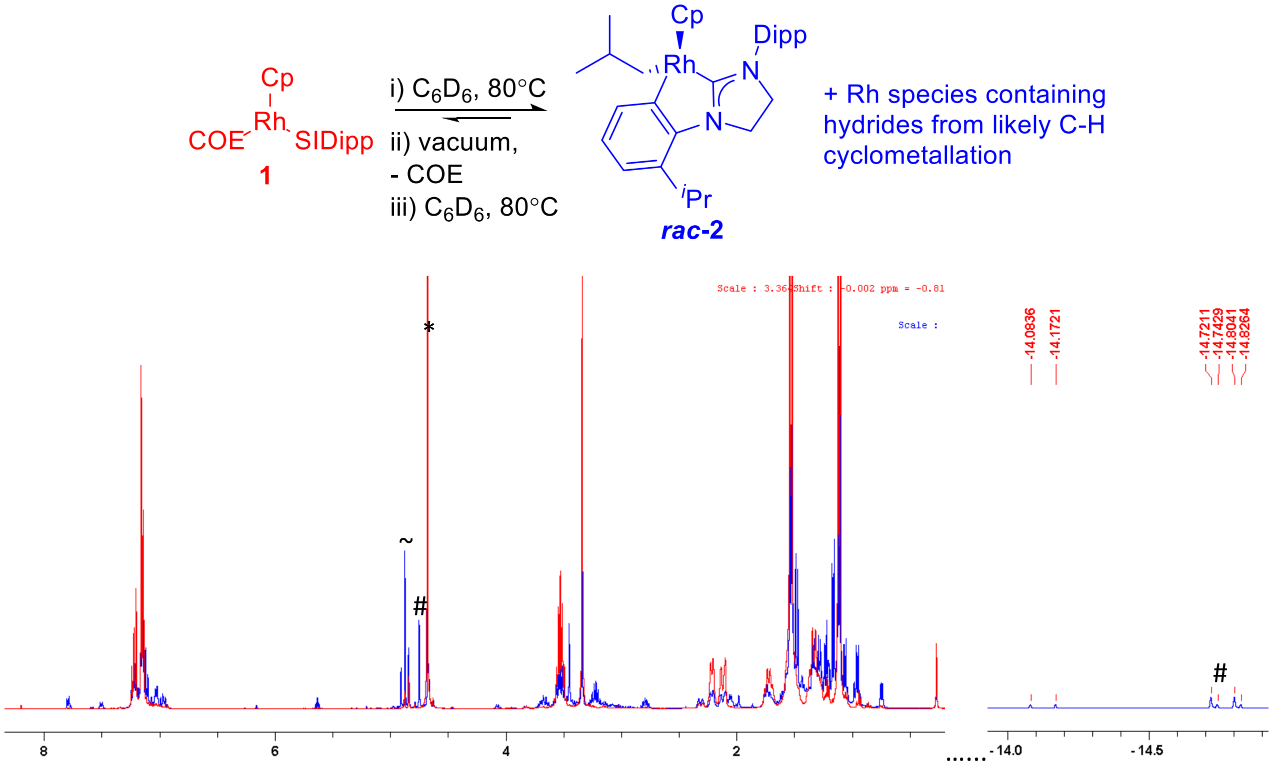

Figure S7. ^{1}H NMR spectra (400 MHz, C_6D_6, 298 K) of [RhCp(SIDipp)(COE)] (**1**) before and after heating at 80°C, removal of all volatiles under vacuum, dissolution in C_6D_6 and further heating at 80°C. Red spectrum is pure [RhCp(SIDipp)(COE)] (**1**) starting material; blue spectrum shows a mixture of **1**, ***rac*-2** and at least two further species that feature Rh hydride moieties. When the largest hydride doublet resonance, denoted #, is integrated and set to 1, the Cp resonance denoted # integrates to approximately 5. This complex is tentatively assigned as a product of C-H cyclometallation of an NHC Dipp group.

2.4 Thermal stability of [Rh(Ind)(SIDipp)(COE)]

A solution of [Rh(Ind)(SIDipp)(COE)] (9 mg) in C_6D_6 (0.7 cm^3) was heated to 80°C and the reaction monitored by ^1H NMR spectroscopy.

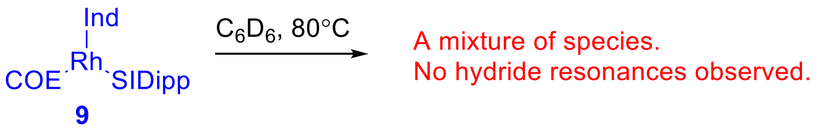

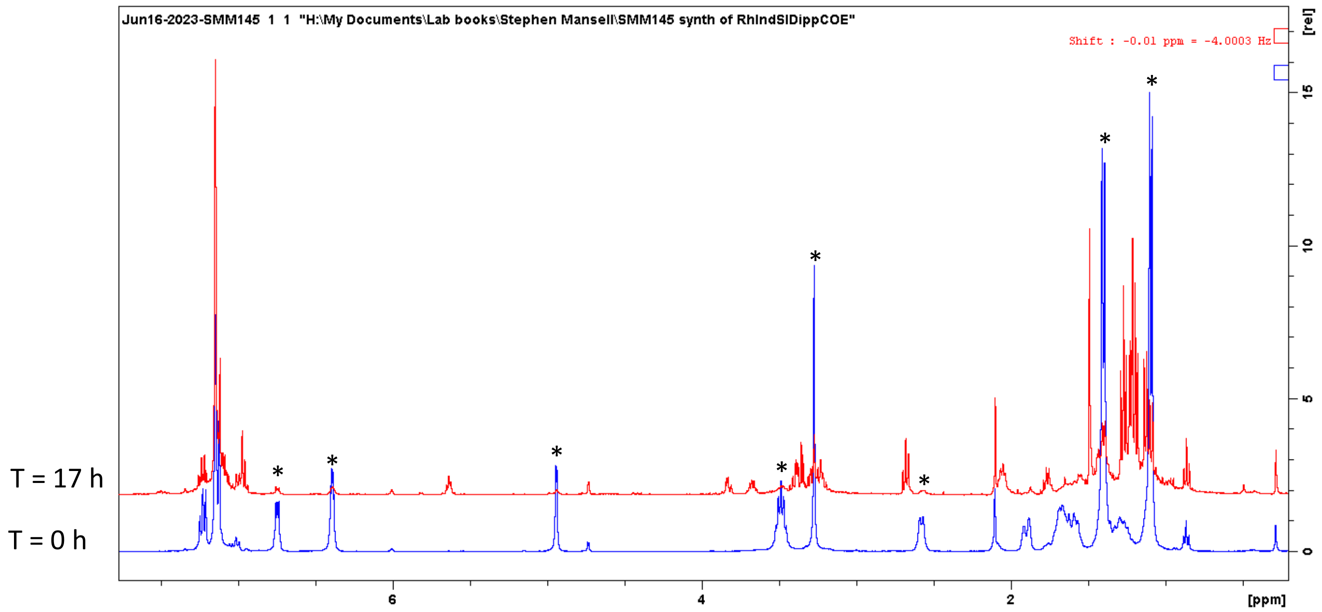

Figure S8. ^1H NMR spectra (400 MHz, C_6D_6, 298 K) of [Rh(Ind)(SIDipp)(COE)] after heating. * indicates [Rh(Ind)(SIDipp)(COE)] starting material. No hydrides were observed in either spectrum (no resonances between 0 and -30 ppm).

2.5 Synthesis of [RhCp{Si(OEt)$_3$}(H)(SIDipp)] (rac-3)

In the glovebox, [Rh(Cp)(SIDipp)(COE)] (**1**) (13 mg, 0.019 mmol) was dissolved in C_6D_6 (0.7 cm^3) then added to a vial containing $(EtO)_3SiH$ (10 mg, 0.058 mmol, 3 eq.). The reaction mixture was transferred to an NMR tube equipped with a J. Young tap and the reaction progress monitored by ^{1}H NMR spectroscopy. 3 d at 20°C led to almost no change, so the reaction was heated to 80°C for 6 h 15 min; ^{1}H NMR spectroscopy showed almost complete conversion into a new Rh complex. The volatiles were then removed in vacuo and the residue extracted into pet. ether (1 cm^3), and the product crystallised as a few colourless crystals upon storage at -25°C after 2 d.

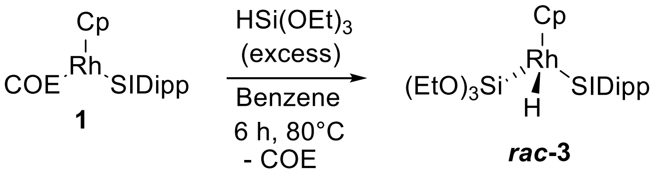

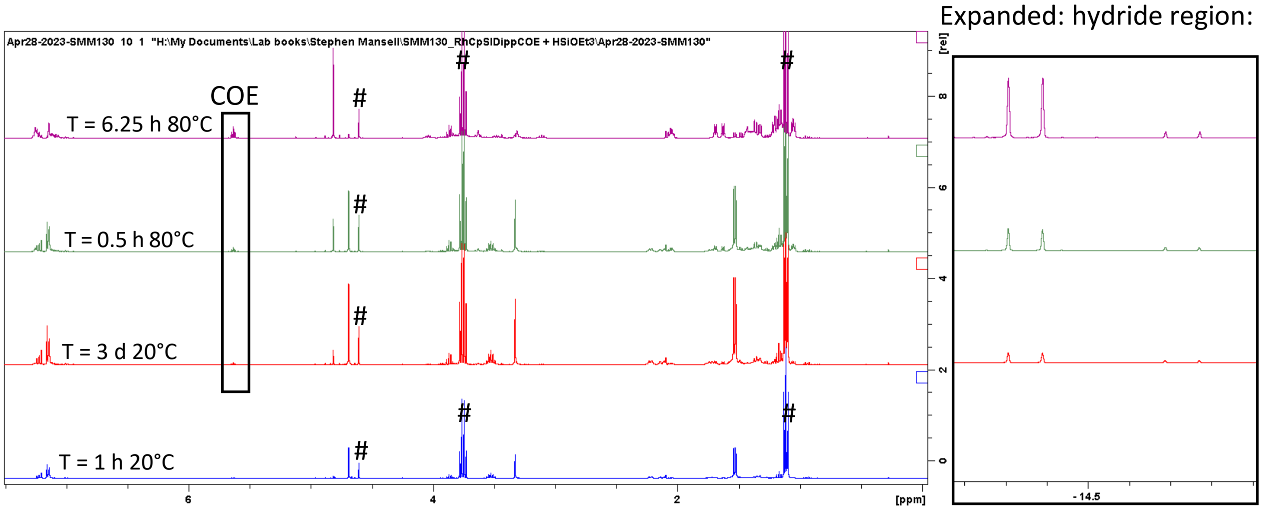

Figure S9. ^{1}H NMR spectra (400 MHz, C_6D_6, 298 K) of [Rh(Cp)(SIDipp)(COE)] reacting with $HSi(OEt)_3$; # = $HSi(OEt)_3$. Hydride resonance: -14.35 ppm, 33.3 Hz.

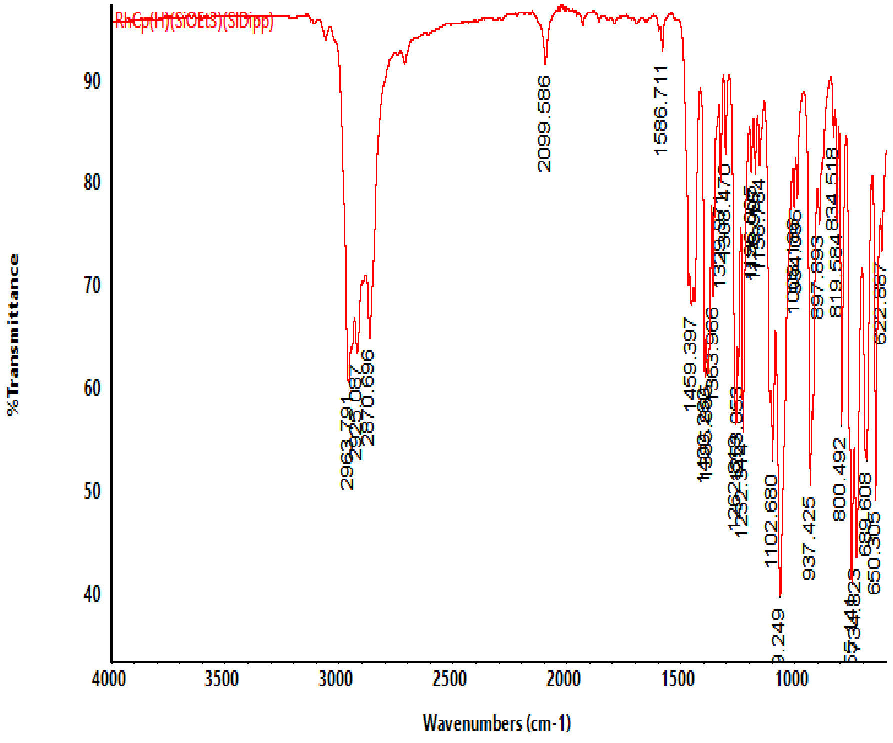

Figure S10. IR spectrum (ATR, C_6D_6 solution) of [RhCp{Si(OEt)$_3$}(H)(SIDipp)] (***rac*-3**).

2.6 Synthetic routes to [Rh(Ind)(NHC)(alkene)]

2.6.1 [Rh(Ind)(IDipp)(COE)] (4)

[Rh(Ind)$(COE)_2$] (369 mg, 0.81 mmol), IDipp (329 mg, 0.81 mmol) and toluene (5 cm^3) were combined in a flask equipped with a J. Young cap in a glovebox. The flask was then taken out of the glovebox and stirred at 60 °C for 16 hours. The solvent was removed under vacuum giving a red-orange glass. The solid was then washed with n-pentane (3 x 10 cm^3) and dried thoroughly under vacuum to yield a yellow powder (362 mg, 0.505 mmol, 62%). Single crystals suitable for X-ray diffraction were grown from a toluene/pentane (1:5) solution.

^{1}H NMR (400 MHz, C_6D_6, 298 K): δ = 7.29-7.24 (m, 2H, Ar), 7.14 (s, 2H, Ar), 6.83-6.80 (m, 2H, Ar), 6.60 – 6.58 (m, 2H, Ar), 6.33 (s, 2H, NHC C*H*=C*H*), 6.28 (q, J = 2.7 Hz, 1H, IndH), 4.82 (d, J= 2.8 Hz, 2H, Ar), 3.24 (sept., J = 6.8 Hz, 4H, $CHMe_2$), 1.37 (d, J = 6.8 Hz 12H, CH_3), 0.97 (d, J = 6.8 Hz, 12H, CH_3); **^{13}C{^{1}H} NMR (400 MHz, C_6D_6,298 K)**: δ = 186.1 (d, J = 73.7 Hz, carbene C), 145.9 (Ar C), 139.1 (Ar C), 129.4 (Ar 2 x CH), 124.7 (d, J = 1.4 Hz, NCHCHN, 2 x CH), 121.8 (Ar C), 120.9 (Ar CH), 116.9 (Ar CH), 96.4 (d, J= 6.8 Hz, Ar CH), 73.1 (d, J= 4.2 Hz, Ar CH), 60.2 (d, J = 15.5 Hz, COE HC=CH), 33.3 (COE CH_2), 32.5 (COE CH_2), 29.0 (IDipp CH), 27.2 (COE CH_2), 25.5 (CH_3), 23.2 (CH_3); **Elemental analysis** calcd (%) for $C_{44}H_{57}N_2Rh$: C 73.72, H 8.01, N 3.91; found C 73.66, H 8.62, N 3.86; **UV-vis**: 5320 $dm^3mol^{-1}cm^{-1}$ at λ_{max} 399 nm.

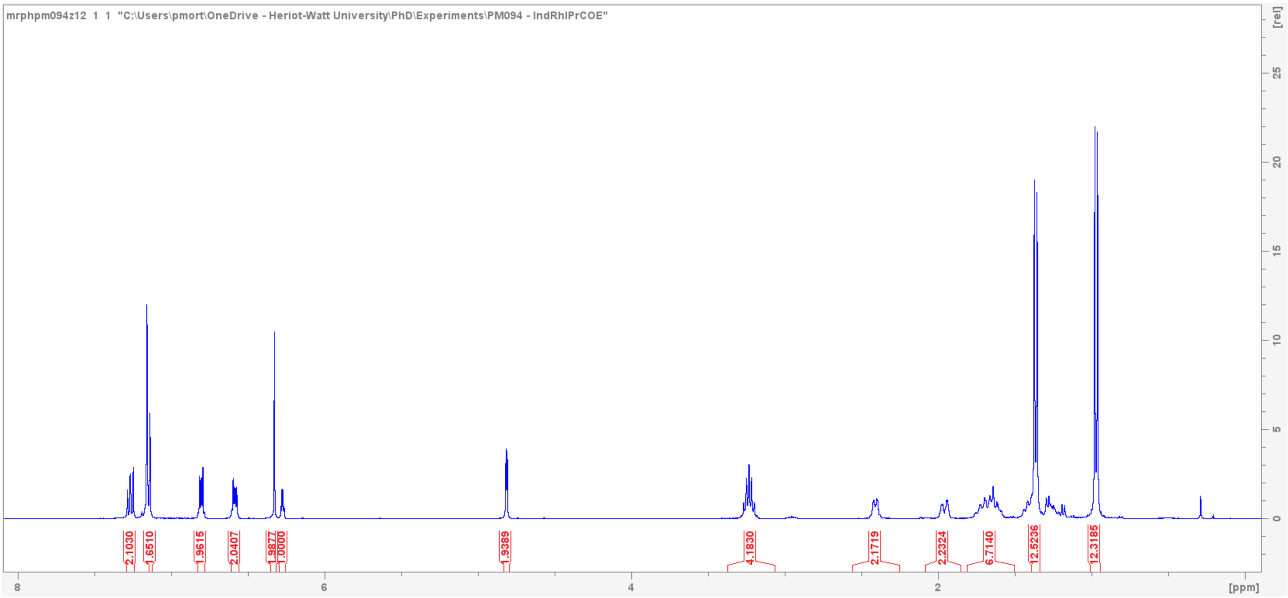

Figure S11. ^{1}H NMR spectrum (400 MHz, C_6D_6, 298 K) of [Rh(Ind)(IDipp)(COE)] (**4**).

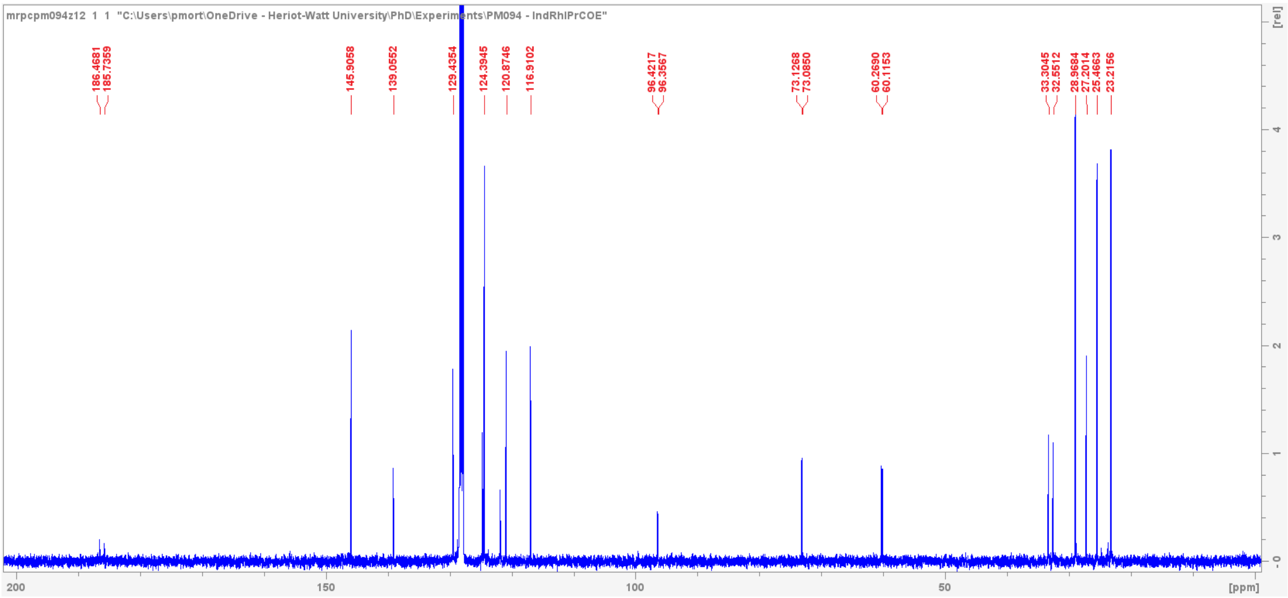

Figure S12. $^{13}C\{^{1}H\}$ NMR spectrum (101 MHz, C_6D_6, 298 K) of [Rh(Ind)(IDipp)(COE)] (**4**).

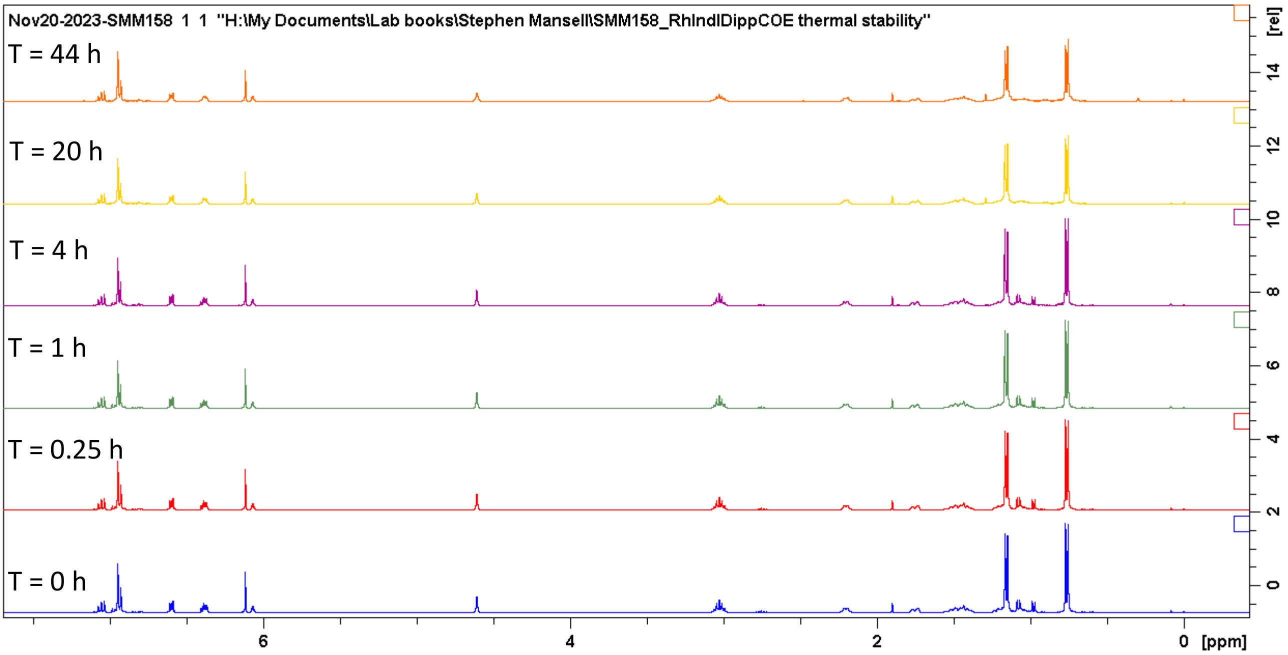

Figure S13. ^{1}H NMR spectra (400 MHz, C_6D_6, 298 K) of [Rh(Ind)(IDipp)(COE)] (**4**) with heating at 80°C revealing almost no change /decomposition.

2.6.2 [Rh(Ind)(IDipp)(CO)] (5)

Method A

[Rh(Ind)(IDipp)(COE)] (**4**) (80 mg, 0.112 mmol) was dissolved in toluene (5 cm^3) in a flask equipped with a J. Young cap in a glovebox. The solution was degassed at -78°C and CO (1 atm) was added. The solution was allowed to return to room temperature with stirring and turned redder in colour. Toluene was removed under reduced pressure to yield an orange powder (67 mg, 0.106 mmol, 95 %). Single crystals suitable for X-ray diffraction were grown from toluene/pentane (1:5) solution.

Method B

$[Rh(\mu\text{-}Cl)(CO)_2]_2$ (10 mg, 0.026 mmol, 0.5 equiv.), lithium indenide (6.2 mg, 0.051 mmol) and C_6D_6 (0.7 cm3) were combined in an NMR tube fitted with J. Youngs tap. The solution was shaken and left to react for an hour before IDipp (20 mg, 0.051 mmol) was added. The vessel was then shaken and left to react overnight at room temperature.

^{1}H NMR (400 MHz, C_6D_6, 298 K): δ = 7.27-7.23 (m, 2H, Ar), 7.13 (s, 2H, Ar), 7.11 (s, 2H, Ar), 6.80-6.76 (m, 2H, Ar), 6.73 – 6.71 (m, 2H, Ar), 6.50 (s, 2H, NHC C*H*=C*H*), 6.05 (q, J = 2.8 Hz, 1H, IndH), 4.96 (d, J= 2.9 Hz, 2H, Ar), 2.94 (sept., J = 6.8 Hz, 4H, $CHMe_2$) 1.28 (d, J = 6.8 Hz 12H, CH_3), 1.01 (d, J = 6.8, 12H, CH_3); **^{13}C{^{1}H} NMR (400 MHz, C_6D_6, 298 K)**: δ = 195.1 (d, J = 92.3, CO), 186.9 (d, J = 72.5 Hz, carbene C), 146.2 (Ar C), 138.1 (Ar C), 130.0 (Ar 2 x CH), 124.5 (NCHCHN, 2 x CH), 123.7 (d, J = 1.75, ArC), 122.7 (Ar C), 121.8 (Ar CH), 116.5 (Ar CH), 99.2 (d, J= 6.6 Hz, Ar CH), 70.5 (Ar CH), 29.1 (IDipp CH), 25.5 (CH_3), 22.8 (CH_3); **IR (thin film)/ cm^{-1}**: 1941 (CO); **HRMS (ASAP/TOF)**: Calcd. for $[C_{37}H_{44}N_2O^{103}Rh]^+$: 635.2503 $[M+H]^+$, Found: 635.2501 m/z.

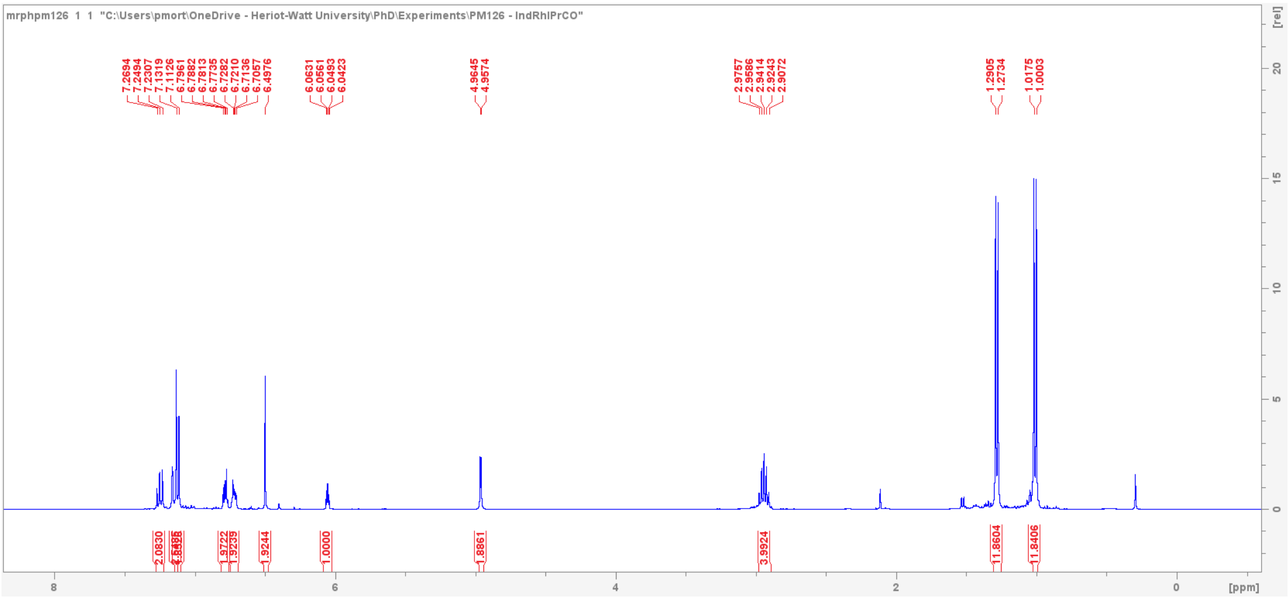

Figure S14. ^{1}H NMR spectrum (400 MHz, C_6D_6, 298 K) of [Rh(Ind)(IDipp)(CO)] (**5**).

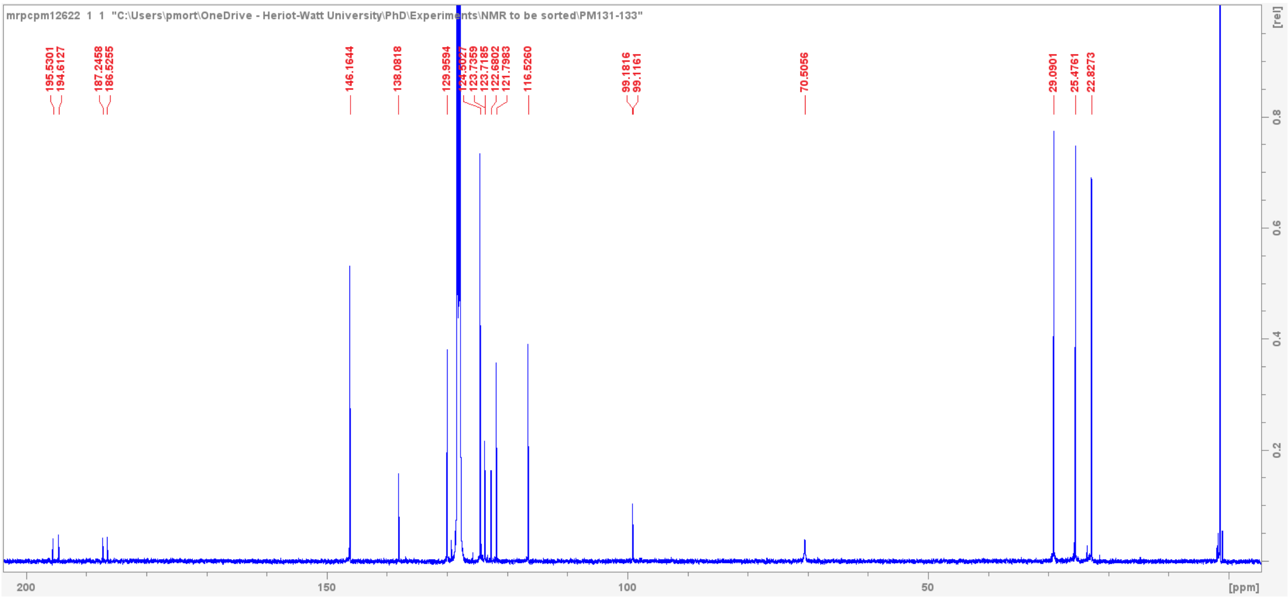

Figure S15. ^{13}C{^{1}H} NMR spectrum (101 MHz, C_6D_6, 298 K) of [Rh(Ind)(IDipp)(CO)] (**5**).

2.6.3 [Rh(Ind)(IMe$_4$)(COE)] (6)

[Rh(Ind)(COE)$_2$] (300 mg, 0.67 mmol), IMe$_4$ (101 mg, 0.82 mmol, 1.4 equiv.) and toluene (5 cm^3) were combined in a flask equipped with a J. Young cap in a glovebox. The flask was then taken out of the glovebox and stirred at 80 °C for 48 hours. The crude product was then filtered through Celite® and the solvent removed under reduced pressure to give a brown powder (96 mg, 0.21 mmol, 32 %yield).

^{1}H NMR (400 MHz, C_6D_6, 298 K): δ = 7.31-7.28 (m, 2H, Ar), 7.13 (s, 1H, Ar), 7.12-7.11 (m, 1H, Ar), 6.40 (quart., J = 2.5 Hz, 1H, IndH) 5.12 (d, J= 2.7 Hz, 2H, Ar), 3.12 (s, 6H, NCH_3) 1.31 (s, 6H, CH_3). **^{13}C{^{1}H} NMR (400 MHz, C_6D_6,298 K):** δ = 181.1 (d, J = 66.9 Hz, carbene C), 123.6 (Ar C), 119.8 (Ar CH), 118.2 (Ar CH), 116.3 (d, J = 1.1 Hz, NC=CN), 96.1 (d, J= 5.4 Hz, Ar CH), 70.9 (d, J= 4.1 Hz, Ar CH), 56.9 (d, J = 16.6 Hz, COE HC=CH), 35.0 (CH_3), 33.7 (d, J = 1.3, COE CH_2), 33.2 (d, J = 1.9 Hz, COE CH_2), 27.3 (COE CH_2), 8.81 (CH_3); **HRMS (ASAP/TOF):** Calcd. for $C_{24}H_{34}N_2{}^{103}Rh^+$: 453.1772, $[M+H]^+$, Found: 453.1775 m/z.

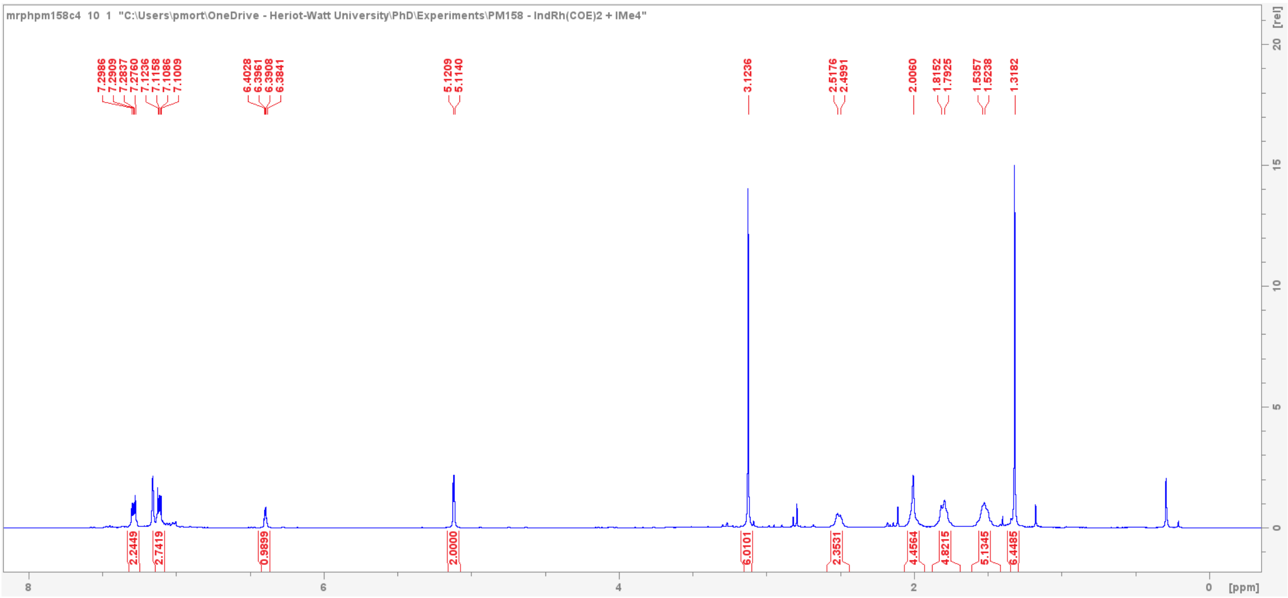

Figure S16. ^{1}H NMR spectrum (400 MHz, C_6D_6, 298 K) of [Rh(Ind)(IMe$_4$)(COE)] (**6**).

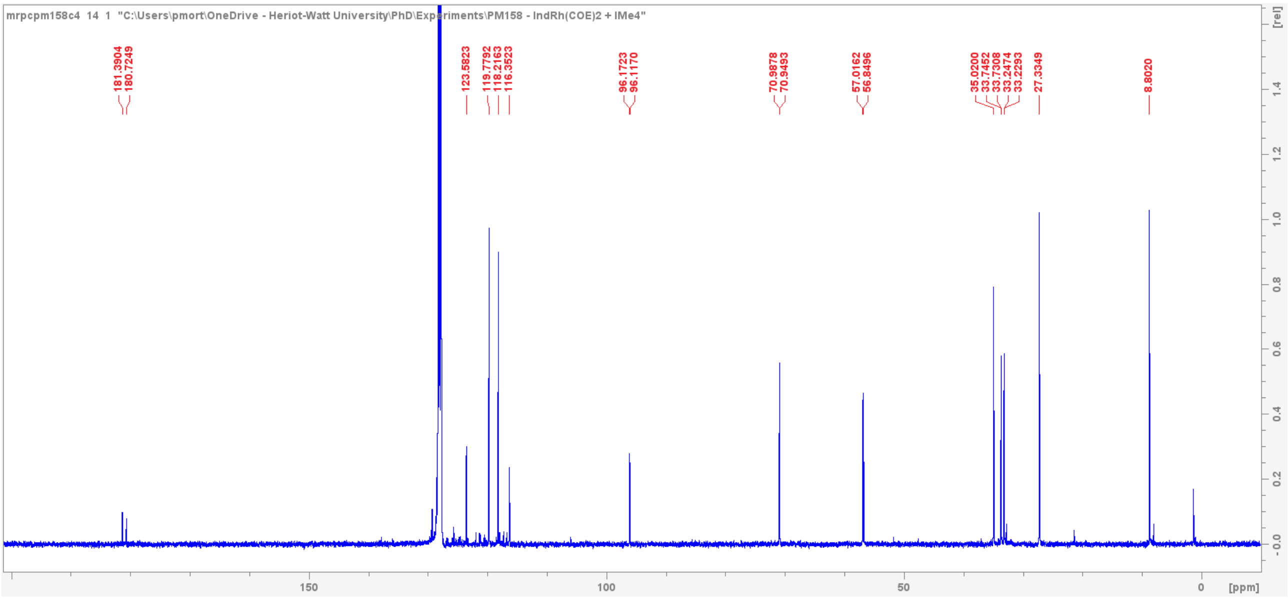

Figure S17. ^{13}C{^{1}H} NMR spectrum (101 MHz, C_6D_6, 298 K) of [Rh(Ind)(IMe$_4$)(COE)] (**6**).

2.7 Synthesis of [Rh(Flu)(COE)$_2$] (7)

To a solution of [Rh(μ-Cl)(COE)$_2$]$_2$ (200 mg, 0.279 mmol) in toluene (5 cm^3) at -78°C, LiFlu (95 mg, 0.548 mmol, 2 equiv.) in toluene (5 cm^3) was then added and stirred for 16 hrs at room temperature. The black suspension was then filtered through a celite® plug, using toluene to wash the product through, to give an orange solution. The solvent was removed under reduced pressure and the product was dried to give a yellow powder (38 mg, 0.078 mmol).

^{1}H NMR (400 MHz, C_6D_6, 298 K): δ = 7.46 -7.44 (m, 2H, Flu H), 7.42 -7.39 (m,2H, Flu H), 7.29 – 7.24 (m, 2H, Flu H), 7.14 – 7.10 (m, 2H, Flu H), 4.67 (s, 1H, Flu-9H). **^{13}C{^{1}H} NMR (101 MHz, C_6D_6, 298 K):** δ = 130.3 (s, Flu CH), 127.1 (s, Flu CH), 123.4 (d, J = 13.1 Hz, Flu CH), 121.2 (s, Flu CH), 119.4 (s, Flu CH), 116.8 (d, J = 3.02 Hz, Flu C), 108.9 (d, J = 1.8 Hz, Flu C), 67.5 (d, J = 6.5 Hz, Rh(HC=CH)), 32.1 (s, CH_2,COE), 29.5 (s, CH_2, COE), 26.8 (s, CH_2, COE). Mass spectrometry revealed no assignable peaks.

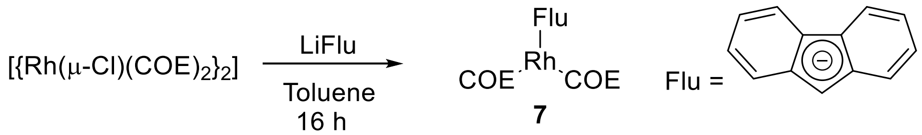

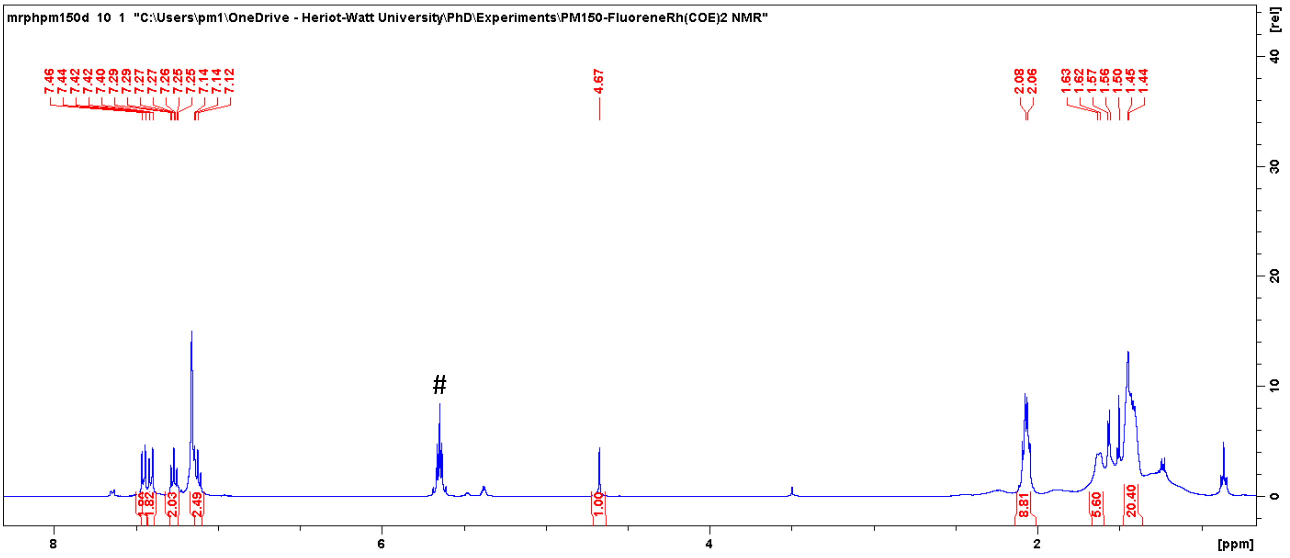

Figure S18. ^{1}H NMR spectrum (400 MHz, C_6D_6, 298 K) of [Rh(Flu)(COE)$_2$] (**7**), unpurified reaction mixture. # is free COE.

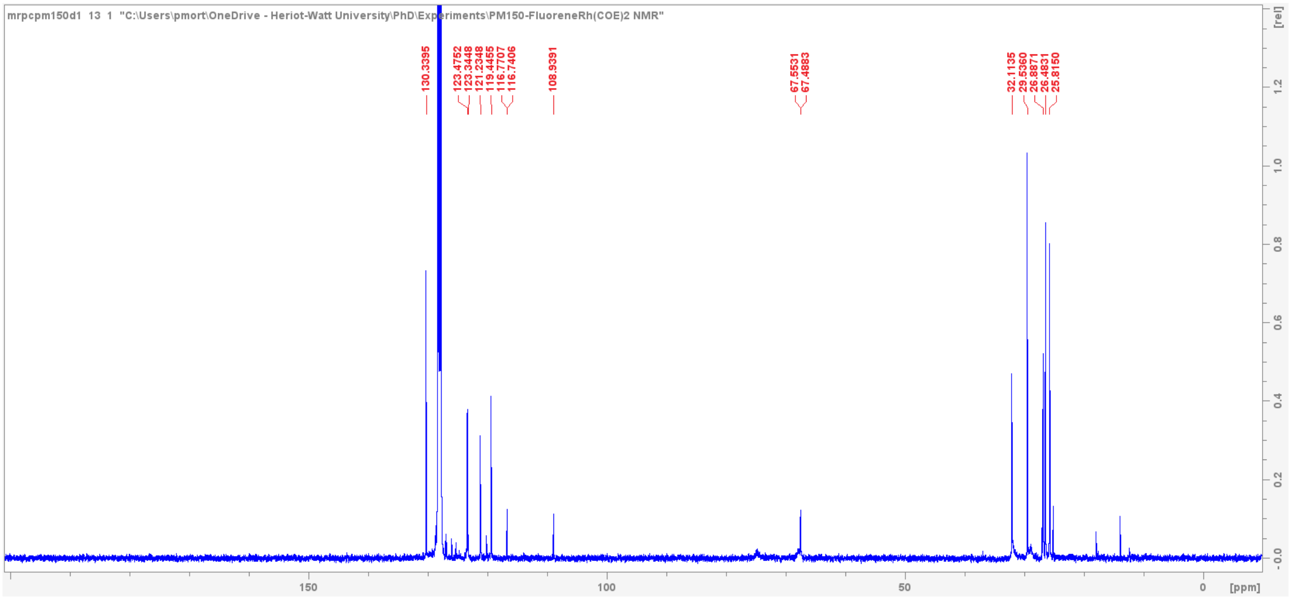

Figure S19. $^{13}C\{^1H\}$ NMR spectrum (101 MHz, C_6D_6, 298 K) of $[Rh(Flu)(COE)_2]$ (**7**).

2.8 Synthesis of $[Rh(Flu\text{-}H_4)(COE)_2]$ (8)

To a solution of tetrahydrofluorene (180 mg, 1.06 mmol) in THF (10 cm^3), n-BuLi (0.7 cm^3, 1.06 mmol) was added at -78°C and the colourless solution became deep yellow. To this solution, $[Rh(\mu\text{-}Cl)(COE)_2]_2$ (380.3 mg, 0.53 mmol, 0.5 equiv) in THF (10 cm^3) was added at -78°C. The solution was allowed to warm to room temperature to give a black suspension. The reaction was allowed to stir for a further 16 hrs. All solvent was removed under reduced pressure then filtered through a celite® plug with toluene to give an orange solution. All volatiles were removed under reduced pressure and the product was dried to give a brown oil (133.8 mg, 0.27 mmol, 26 %yield). The product crystallised over time to yield single crystals suitable for X-ray diffraction.

^1H NMR (400 MHz, C_6D_6, 298 K): δ = 7.26-7.22 (m, 1H, Flu H), 7.19-7.14 (m,1H, Flu H), 7.11-7.06 (m, 1H, Flu H), 6.83 (dd, *J*= 7.57 and 0.6 Hz, 1H, Flu H), 4.46 (s, 1H, Flu-9H) 2.98-2.93 (m, CH, COE), 2.89-2.79 (m, CH, COE), 2.49-2.40(m, 2CH, COE) 2.32-2.02 (m, COE and CH_2 ($Flu\text{-}H_4$)). **$^{13}C\{^1H\}$ NMR (101 MHz, C_6D_6, 298 K):** δ = 129.3(C), 128.7(C), 128.6(C), 125.7(C), 123.3(CH), 121.5(CH), 120.0(CH), 115.9(CH), 113.4 (d, J = 5.2 Hz, Flu CH(9H)), 78.2 (d, J = 5.6 Hz, Rh(HC=CH)), 40.8(CH_2), 26.8(CH_2), 25.3(CH_2), 23.7(CH_2), 23.6(CH_2), 23.2(CH_2), 22.9(CH_2), 22.5(CH_2) and 21.4(CH_2). **HRMS (ASAP/TOF)**: Calcd. for $[C_{21}H_{26}{}^{103}Rh_2]^+$: 381.1089 $[Rh(Ind)(C_8H_{13})]^+$, Found: m/z. 381.1091. Mass spectrometry revealed a fragment; [M – COE – H].

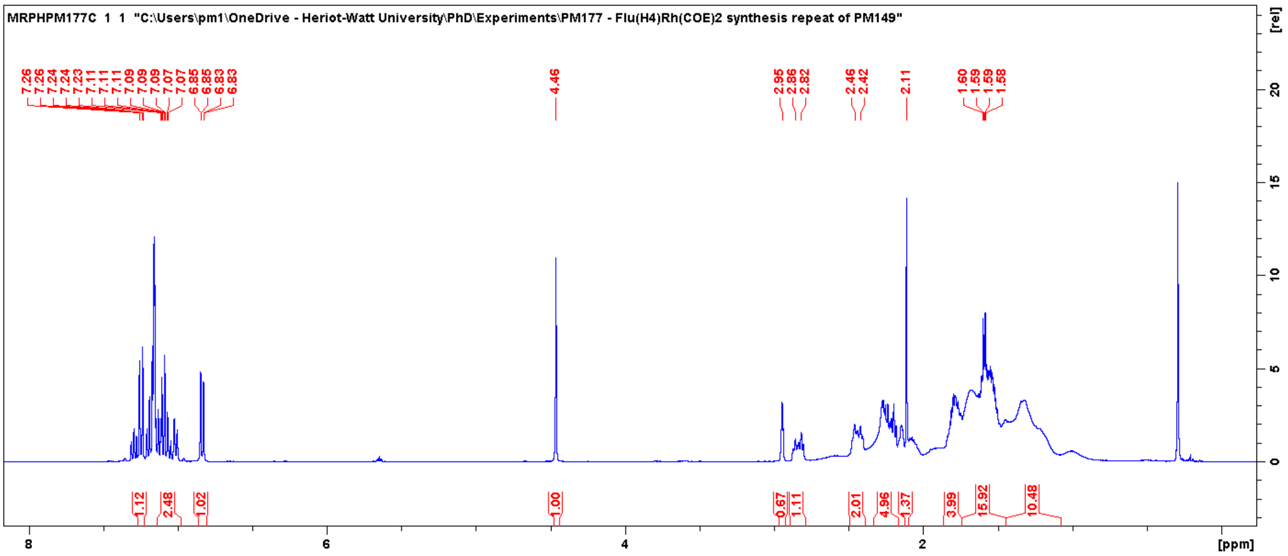

Figure S20. ^{1}H NMR spectrum (400 MHz, C_6D_6, 298 K) of [Rh(Flu-H_4)(COE)$_2$] (**8**).

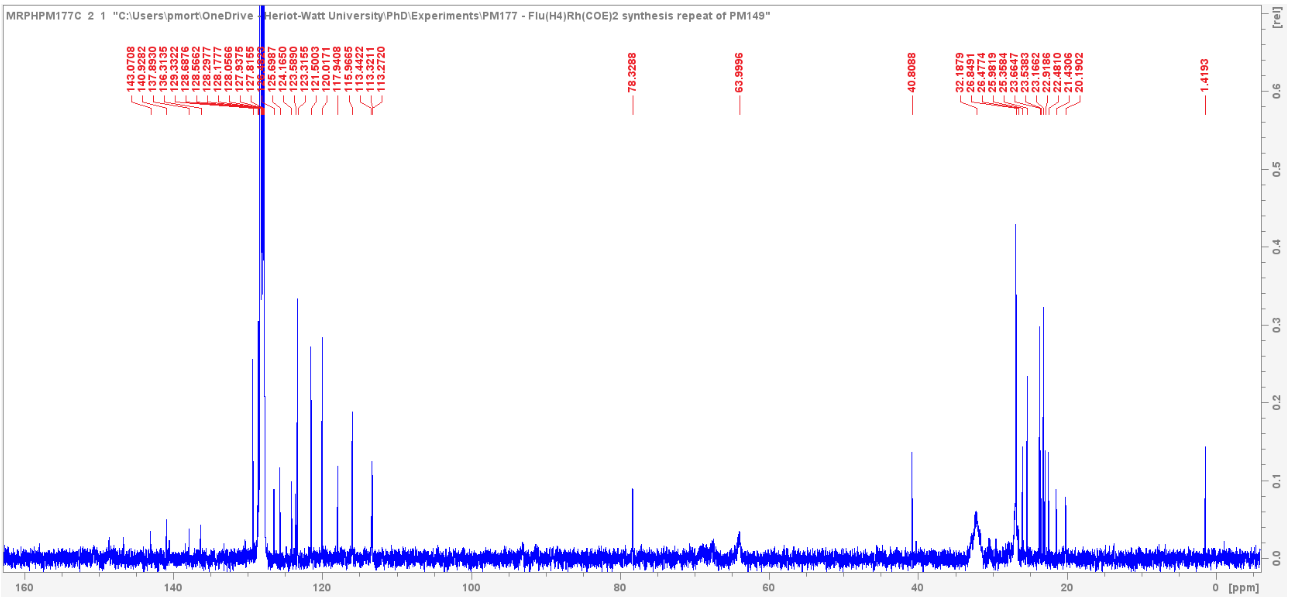

Figure S21. $^{13}C\{^{1}H\}$ NMR spectrum (101 MHz, C_6D_6, 298 K) of [Rh(Flu-H_4)(COE)$_2$] (**8**).

2.9 General procedure for borylation reactions

The reactants were mixed in a flask equipped with a J. Young tap in a glovebox. The flask was then removed from the glovebox and heated using a silicon oil bath at either 80 °C, 110 °C or 140 °C.

2.9.1 Synthesis and characterisation of the alkylBpin GC standards from hydroboration reactions

Decyl boronic acid pinacol ester (decylBpin)

Following a literature procedure,[11] pinacolborane (2,9 cm^3, 20 mmol) was diluted in 1,2-dichloroethane (2 cm^3) and 1-decene (1.9 cm^3, 10 mmol) was added. The reaction mixture was warmed to 40 °C and stirred for 72 h. However, ^1H and ^{11}B NMR spectroscopic analysis revealed very poor conversion, so following analogous procedures,[12] Wilkinson's catalyst ($[RhCl(PPh_3)_3]$, 23 mg, 0.025 mmol, 0.25 mol%) was added and the reaction heated to 50°C for 16 h. The reaction was poured into saturated aqueous NH_4Cl solution (250 mL) then diethyl ether (150 mL was added). The organic phase was separated then washed with saturated aqueous NH_4Cl solution (100 mL) and dried over $MgS0_4$. The solvent was removed in vacuo, affording a residue which was purified by flash column chromatography (2% ether in hexane) providing the desired product as a clear oil (1.553 g, 5.79 mmol, 58% yield). This was further purified by Kugelrohr distillation (2 x10^{-2} mbar, 110°C) to give decylBpin as a colourless oil (1.189 g, 4.43 mmol, 44% yield; GC: 95% pure).

^1H NMR (300 MHz, $CDCl_3$, 298 K): δ = 1.37 (m, 2H, CH_2), 1.26 -1.21 (m, 26H, CH_2 and Bpin-*Me*), 0.85 (t, 3H, CH_3), 0.74 (t, 2H, CH_2). **$^{11}B\{^1H\}$ NMR (128 MHz, $CDCl_3$, 298 K):** δ = 34.1 (s, decylBpin).

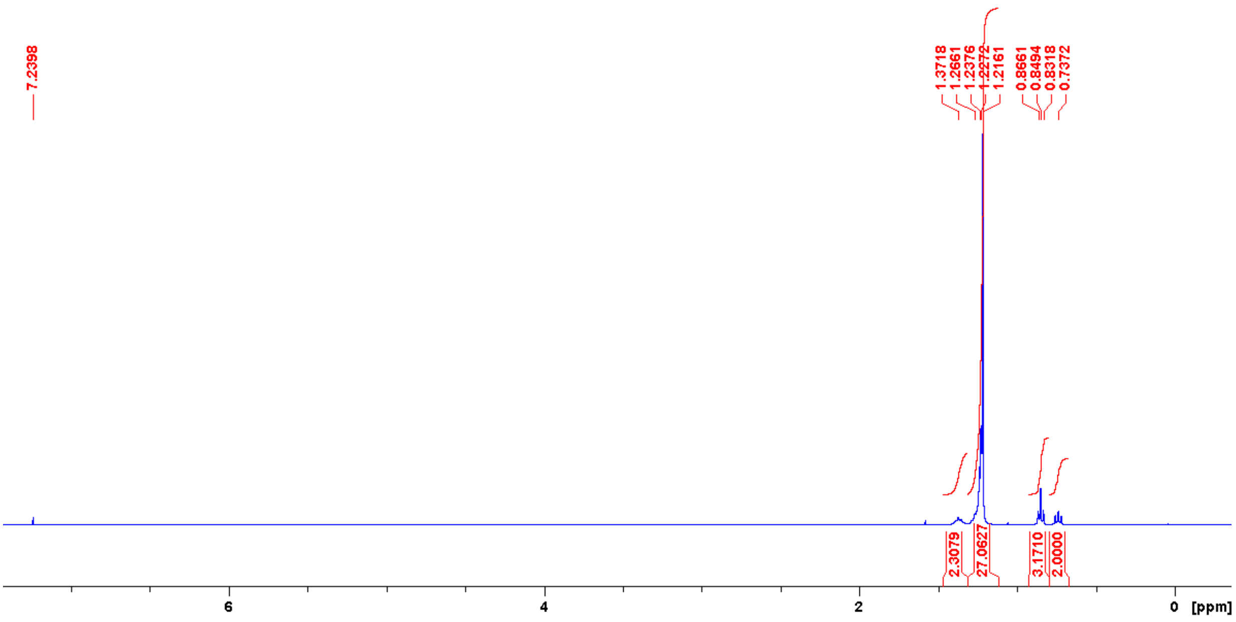

Figure S22. ^1H NMR (400 MHz, $CDCl_3$, 298 K) of decylBpin synthesized from hydroboration of 1-decene using Wilkinson's catalyst.

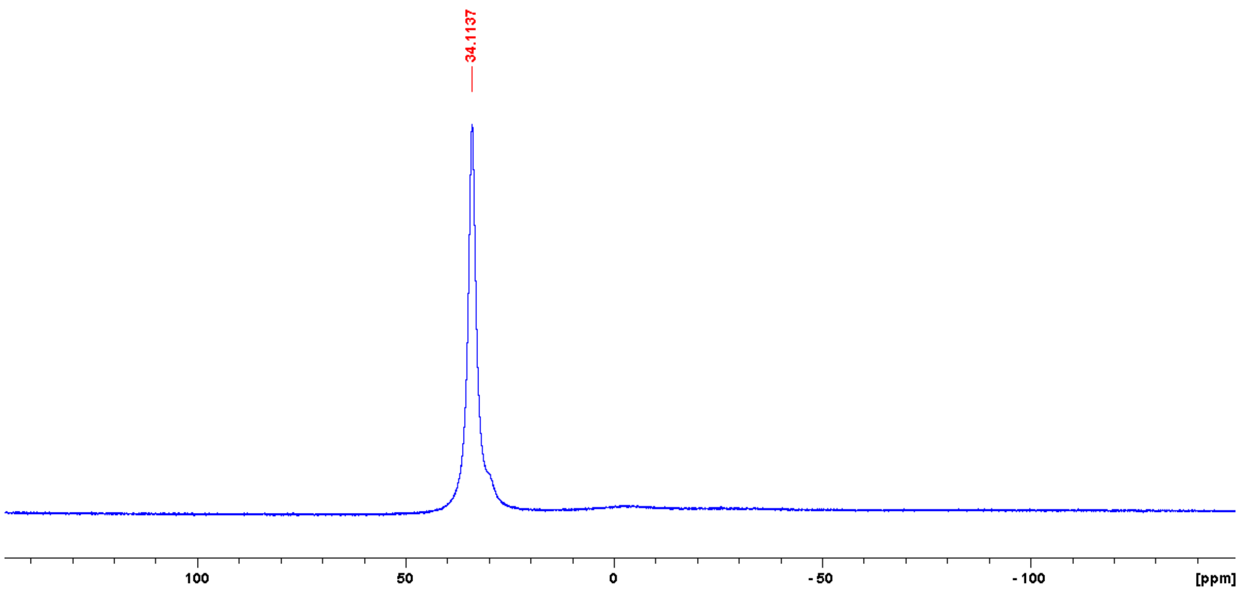

Figure S23. $^{11}B\{^1H\}$ NMR (128 MHz, $CDCl_3$, 298 K) of decylBpin synthesized from hydroboration of 1-decene using Wilkinson's catalyst.

Hexyl boronic acid pinacol ester (hexylBpin)

H-Bpin (1.523 g, 11.90 mmol) and Wilkinson's catalyst [$RhCl(PPh_3)_3$] (0.023 g, 0.0244 mmol, 0.2 mol%) were added to a flask equipped with a J. Young tap in a glove box. Under inert conditions, 1-hexene (1.36 mL, 11.88 mmol) and dry THF (10 mL) were added to the flask. The solution was stirred at ambient temperature for 36 h, after which an aliquot was taken for $^{11}B\{^1H\}$ NMR analysis. The aliquot suggested low conversion; therefore, the temperature was increased to 50 °C for 3 h. All solvent was removed under reduced pressure and the crude product dissolved in pentane was filtered through a silica plug. The solvent was removed under reduced pressure yielding a yellow oil (2.088 g, 9.84 mmol, 83% yield). Following analysis by GC-FID, the product was distilled to improve purity by Kugelrohr distillation (80 °C and 0.42 mbar). The product after distillation was a colourless/clear oil (0.955 g, 4.50 mmol, 38%).

^{1}H NMR (300 MHz, $CDCl_3$, 298 K): δ = 1.37 (m, 2H, CH_2), 1.26 (m, 6 H, CH_2), 1.22 (s, 12 H, Bpin-*Me*), 0.84 (m, 3 H, CH_3), 0.74 (t, 2H, CH_2). **$^{11}B\{^1H\}$ NMR (128 MHz, $CDCl_3$, 298 K):** δ = 34.1 (s, $CH_3(CH_2)_5$-Bpin), **MS (ASAP, API):** Calculated for $[C_{12}H_{25}BO_2 + H]^+$: 213.15, Found: 213.15 $[M + H]^+$.

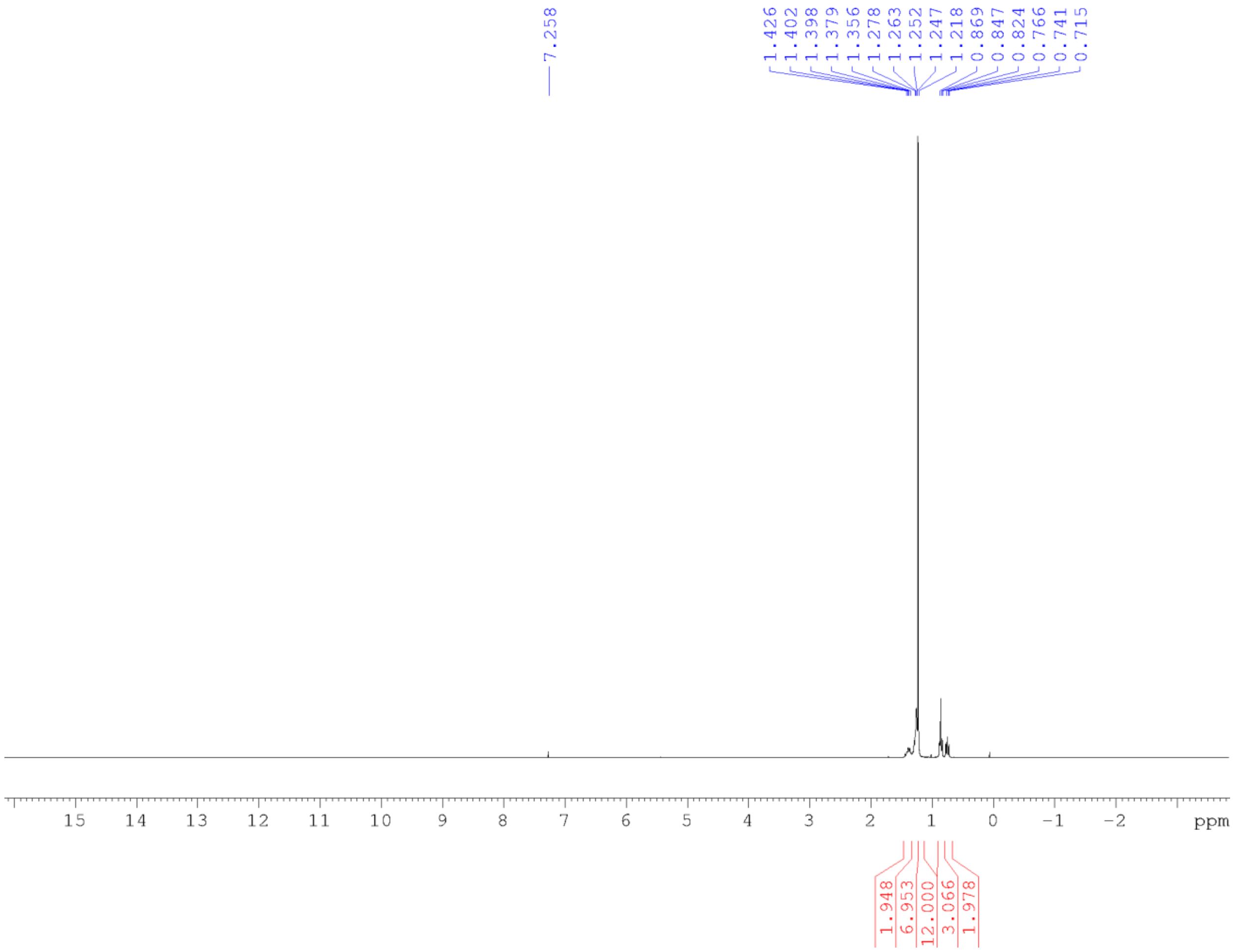

Figure S24. ^{1}H NMR (400 MHz, $CDCl_3$, 298 K) of hex-Bpin synthesized from hydroboration of 1-hexene using Wilkinson's catalyst.

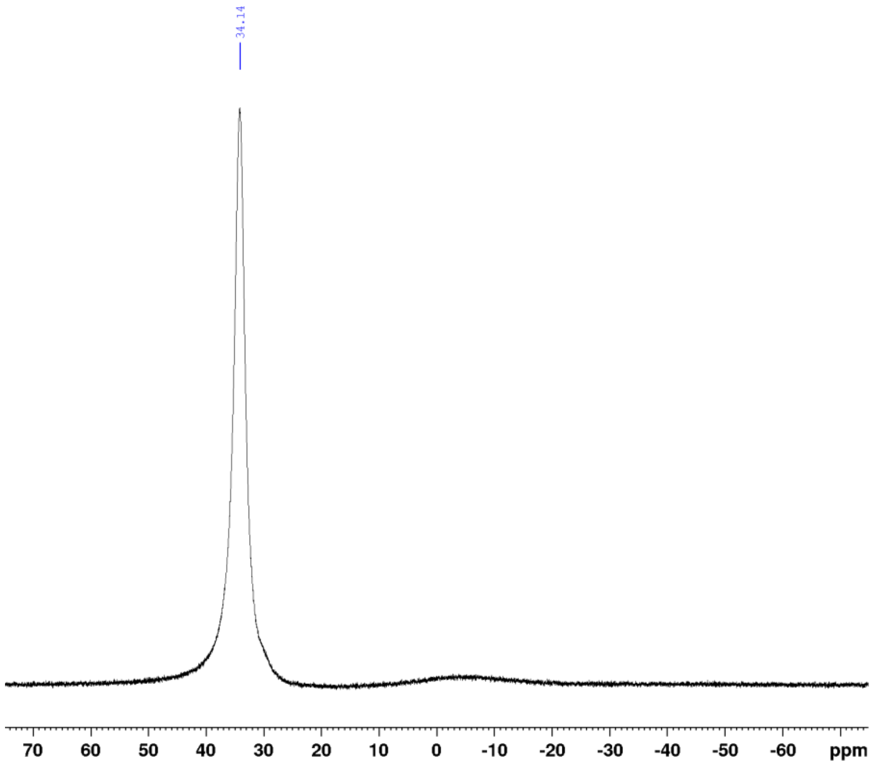

Figure S25. $^{11}B\{^{1}H\}$ NMR (128 MHz, $CDCl_3$, 298 K) of hex-Bpin synthesized from hydroboration of 1-hexene using Wilkinson's catalyst.

GC characterisation of alkyl boranes

GC retention times: hexylBpin: 9.36 min, B_2pin_2: 10.226 min, hexadecane internal standard: 11.211 min, decylBpin: 11.525 min.

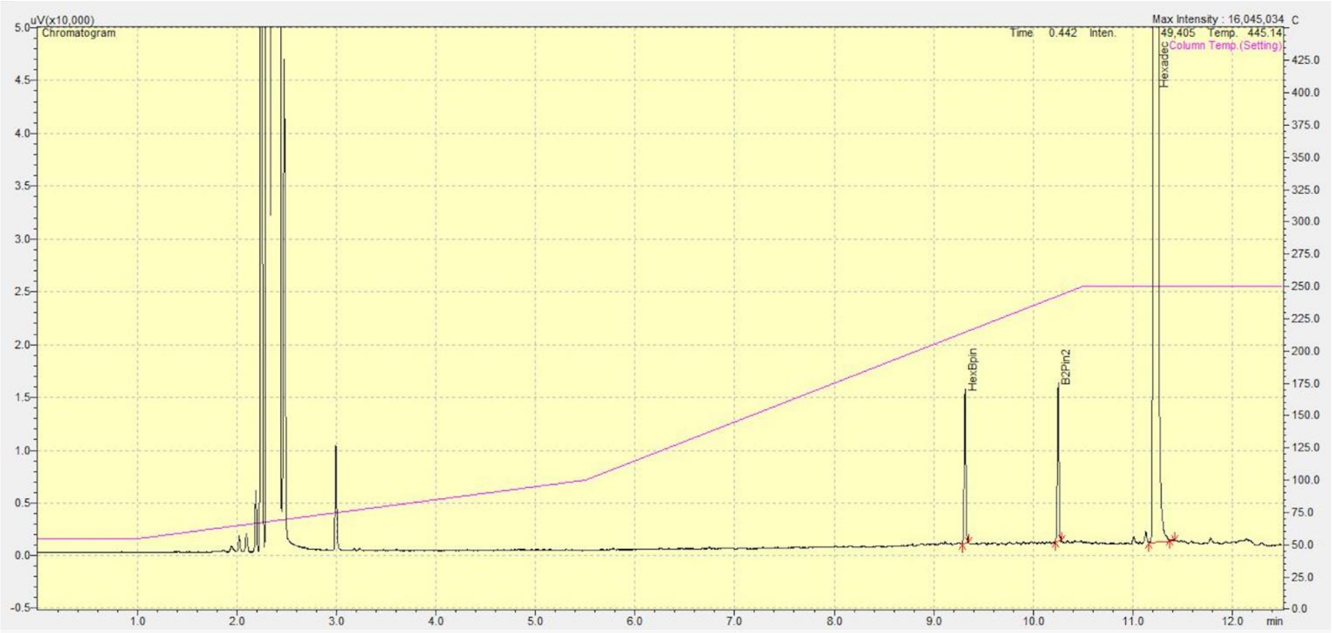

Figure S26. Example chromatogram for the borylation of hexane with B_2pin_2 (2h, 150 °C, 5 mol% [Rh(Ind)(SIDipp)(COE)]).

2.10 ^{1}H NMR spectroscopic monitoring of arene borylation reactions

2.10.1 Benzene C-H borylation

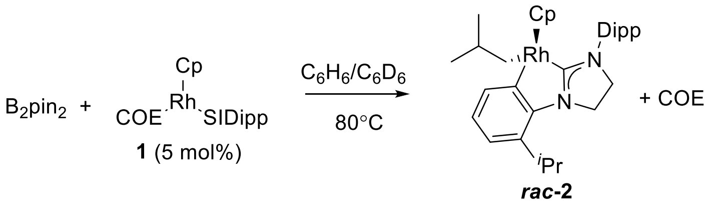

Internal standard: $FeCp_2$.

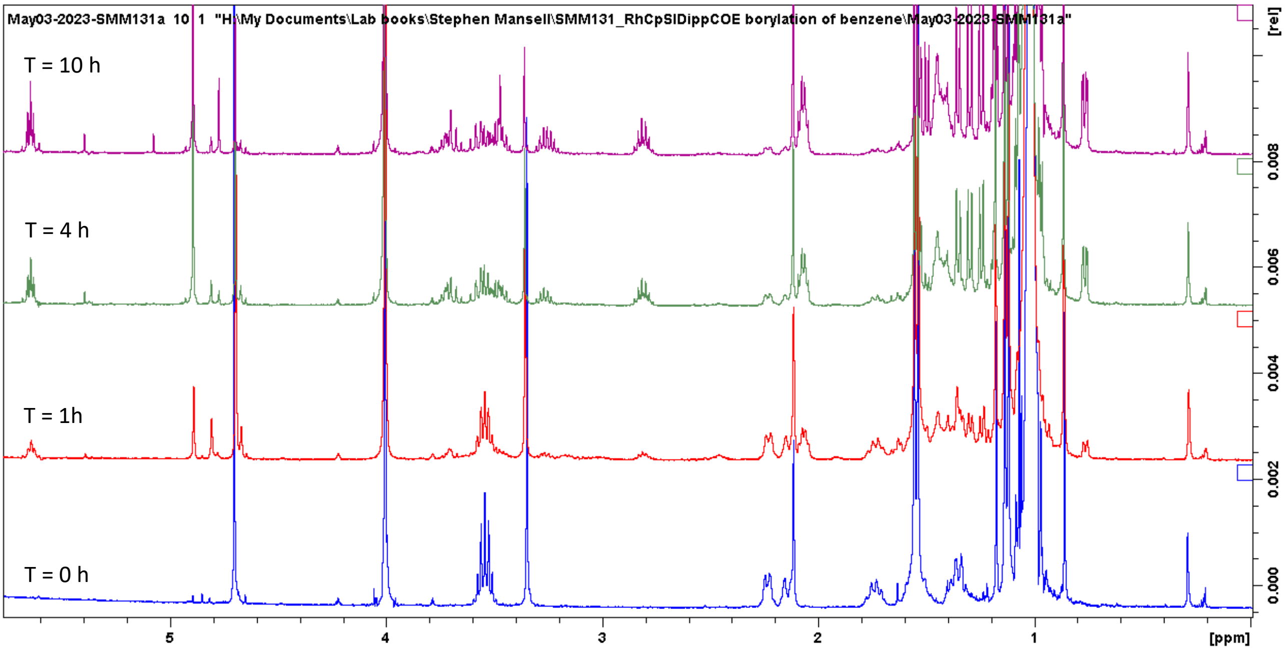

Figure S27. ^{1}H NMR spectroscopic comparison of [RhCp(SIDipp)(COE)] (**1**, 5 mol%) in C_6H_6/C_6D_6 with B_2pin_2 (100 mol%) and ferrocene as an internal standard under heating at 80°C. The C-C bond activation complex ***rac*-2** was the only new Rh species observed.

2.10.2 Toluene borylation

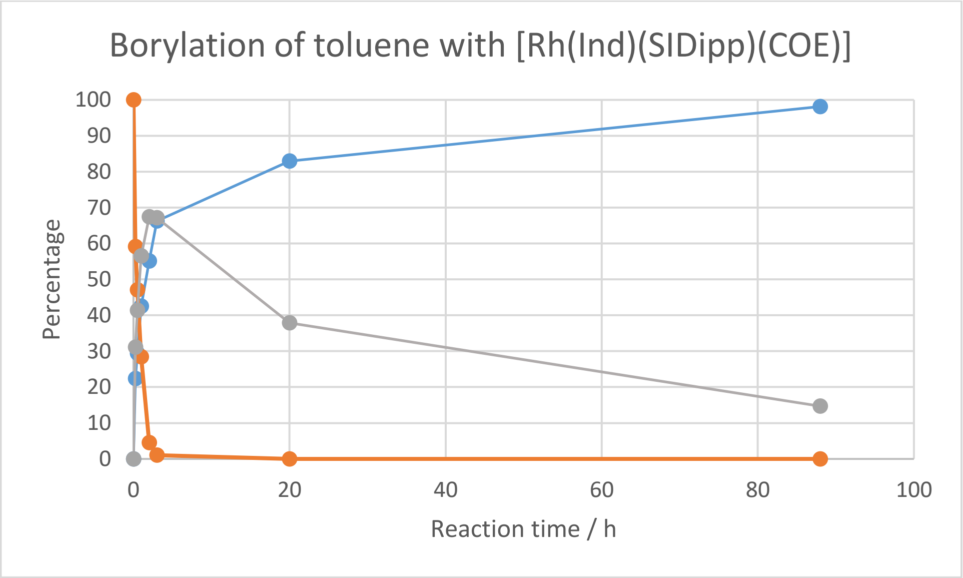

Figure S28. Reaction profile for the catalytic borylation of toluene at 110°C using 5 mol% [Rh(Ind)(SIDipp)(COE)]. Orange line is B_2pin_2, blue line is tolylBpin, grey line is HBpin.

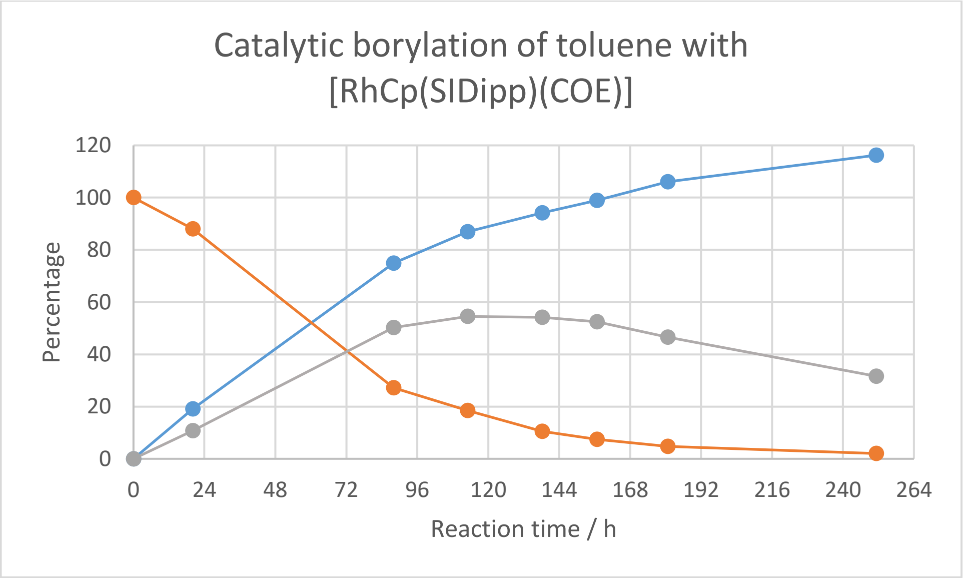

Figure S29. Reaction profile for the catalytic borylation of toluene at 110°C using 5 mol% [RhCp(SIDipp)(COE)] (ferrocene used as internal standard). Orange line is B_2pin_2, blue line is tolylBpin, grey line is HBpin.

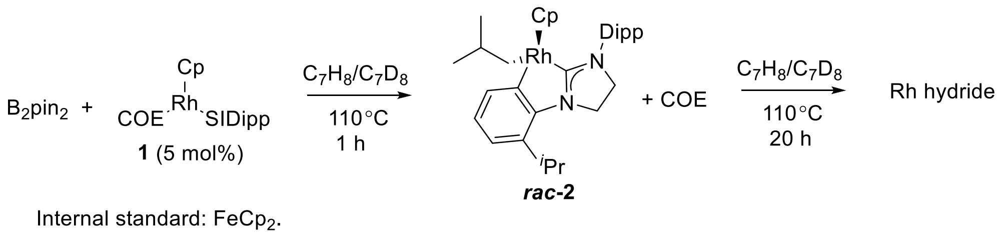

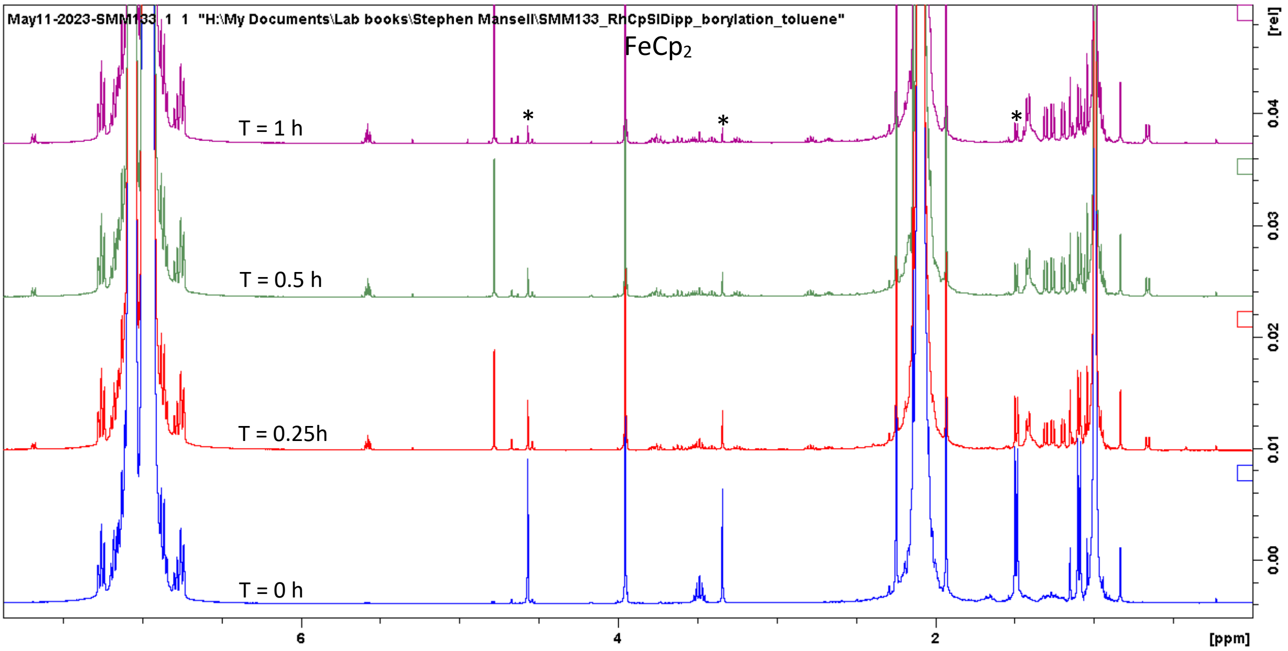

Figure S30. Reaction of [RhCp(SIDipp)(COE)] (5 mol%) in toluene/d^8-toluene with B_2pin_2 (100 mol%) and ferrocene as an internal standard under heating at 110°C. The C-C bond activation complex ***rac*-2** was observed at this stage of the reaction (T = 1 h). * indicates [RhCp(SIDipp)(COE)].

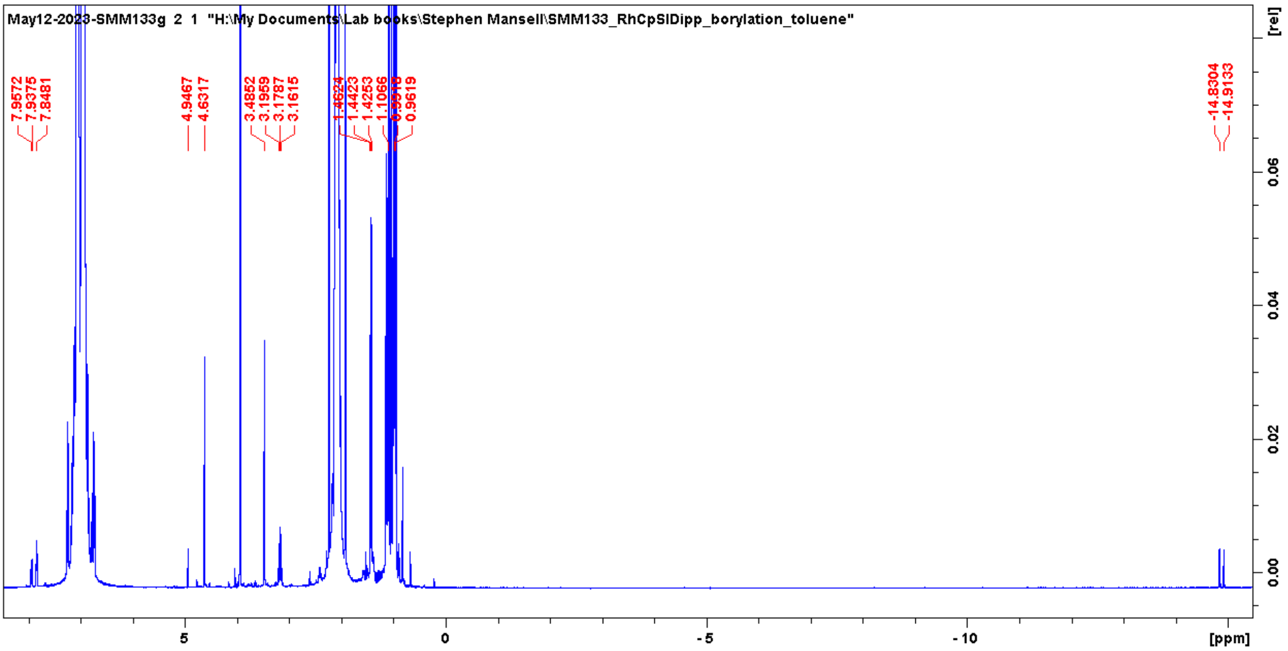

Figure S31. New Rh complex observed after 20 h heating at 110°C in the borylation of toluene/d^8-toluene with B_2pin_2 (100 mol%) using [RhCp(SIDipp)(COE)] (ferrocene as an internal standard). Rh-H resonance: -14.87 ppm, 33.2 Hz.

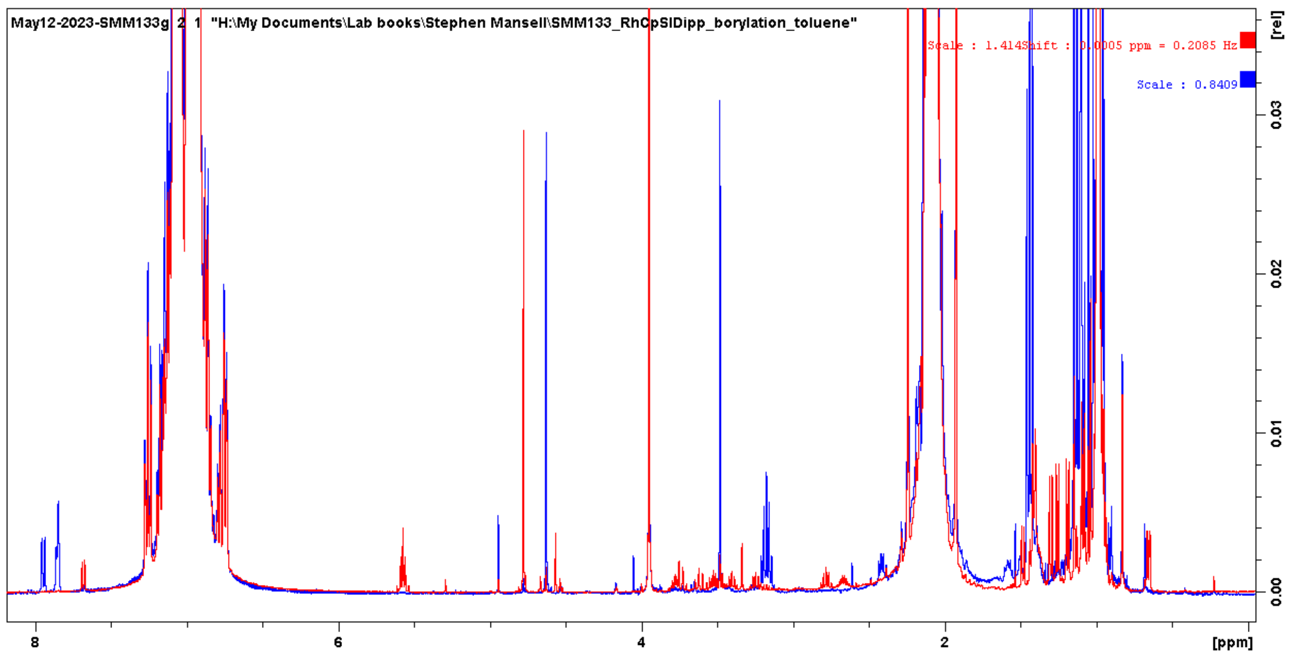

Figure S32. ^{1}H NMR spectroscopic comparison of the different Rh complexes generated from [RhCp(SIDipp)(COE)] after 1 h at 110°C (red: C-C activation product ***rac*-2**) with the new Rh complex observed after 20 h at 110°C (blue).

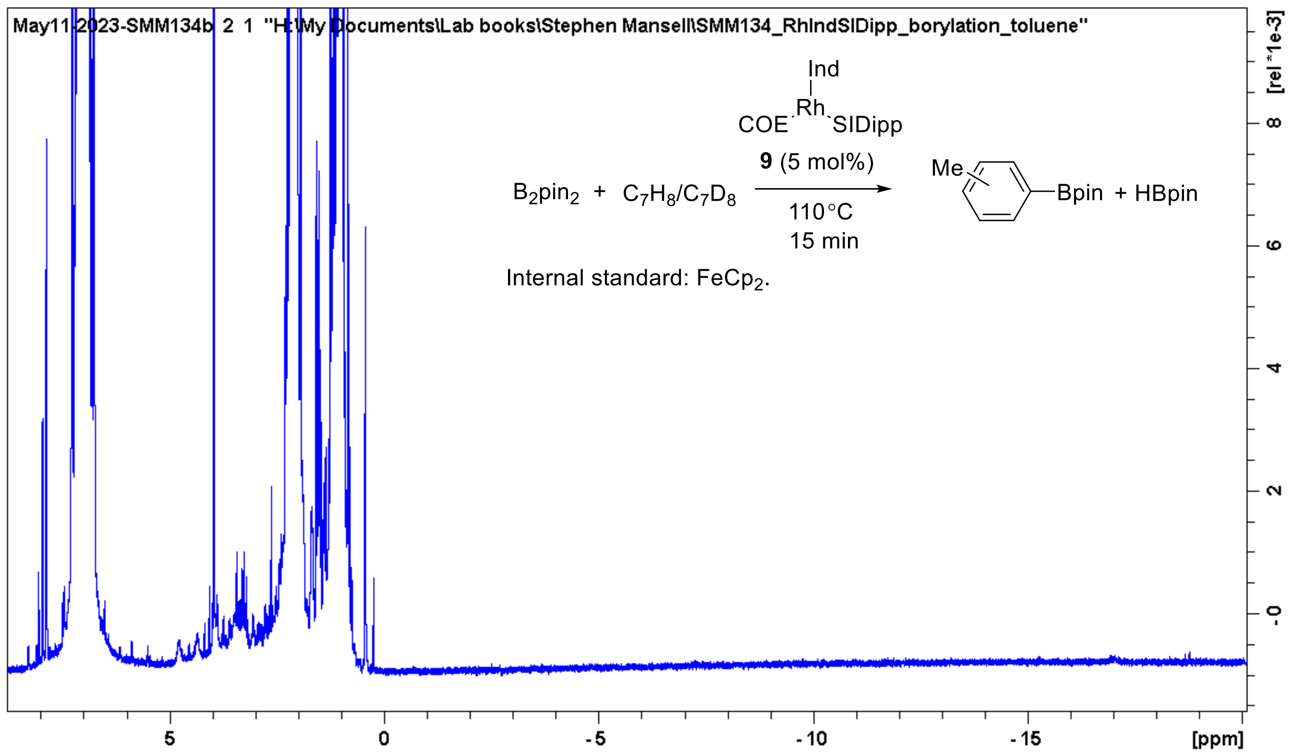

Figure S33. ^{1}H NMR spectrum of 5 mol% [Rh(Ind)(SIDipp)(COE)] with B_2pin_2 (100 mol%) in toluene/d^8-toluene with ferrocene as an internal standard after 15 min heating at 110°C. Unlike for **1**, a distinct intermediate or catalyst resting state was not observed.

2.10.3 Reaction profiles for benzene C-H borylation at 80°C

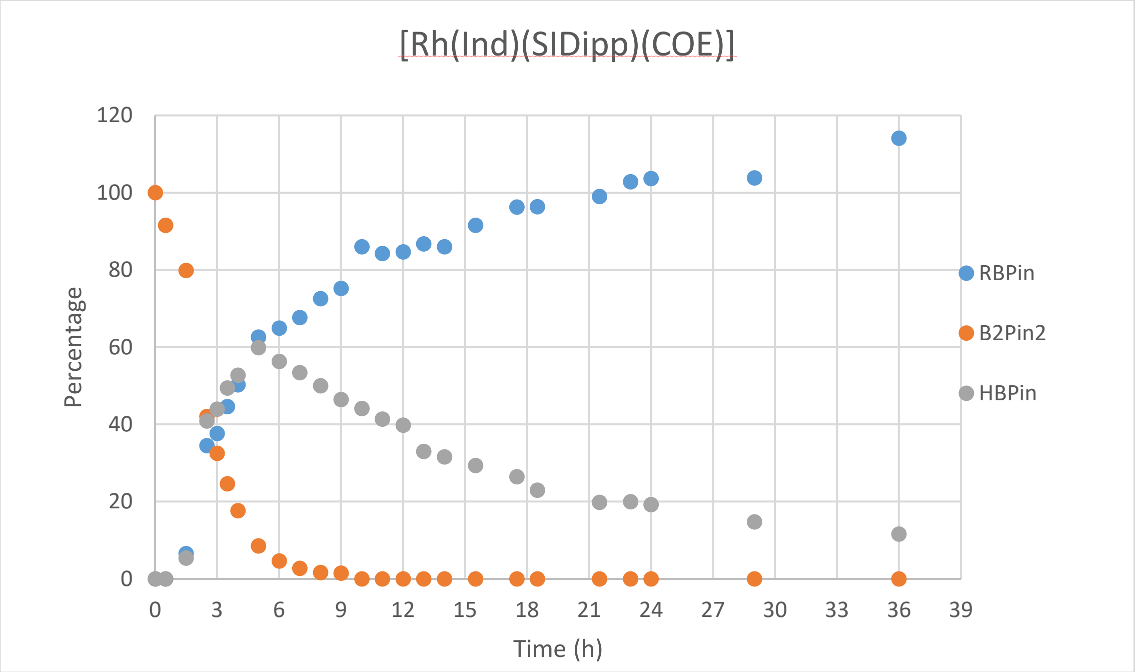

Figure S34. Reaction profile for [Rh(Ind)(SIDipp)(COE)] (**9**)(as catalyst (5 mol%) for the C-H borylation of benzene with B_2pin_2 (80°C, ferrocene used as internal standard). 100% yield is for the reaction of PhH + B_2pin_2 -> PhBpin + HBpin.

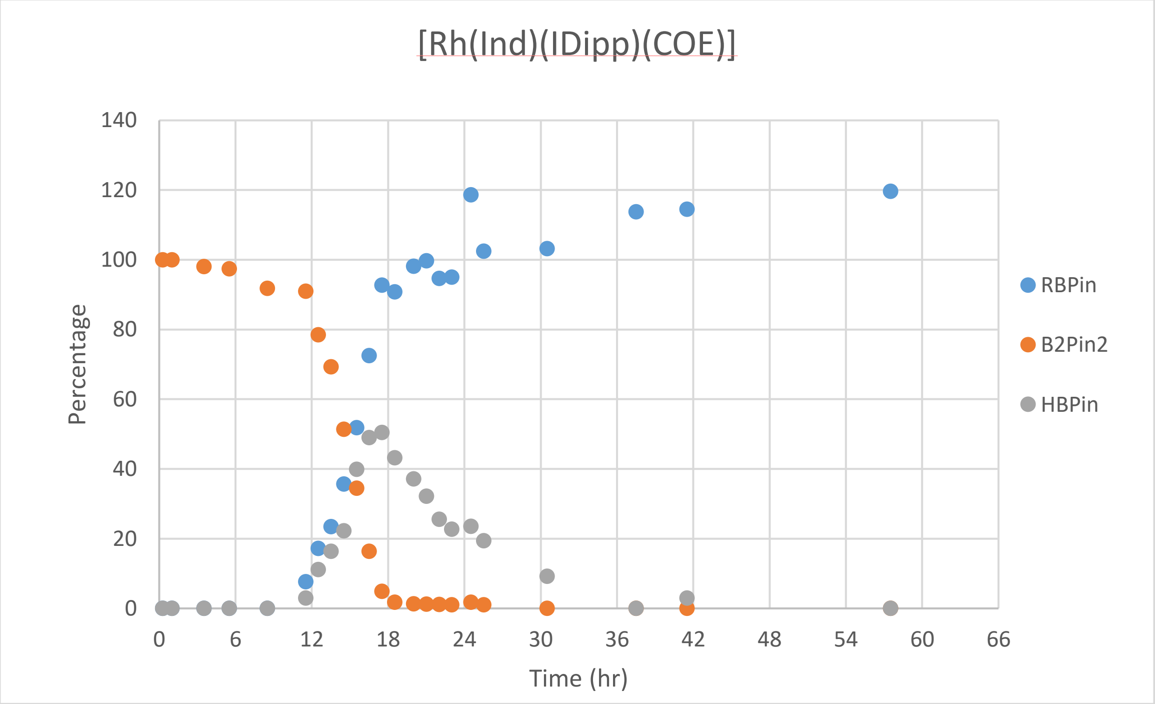

Figure S35. Reaction profile for [Rh(Ind)(IDipp)(COE)] (**4**) as catalyst (5 mol%) for the C-H borylation of benzene with B_2pin_2 (80°C, ferrocene used as internal standard). 100% yield is for the reaction of PhH + B_2pin_2 -> PhBpin + HBpin.

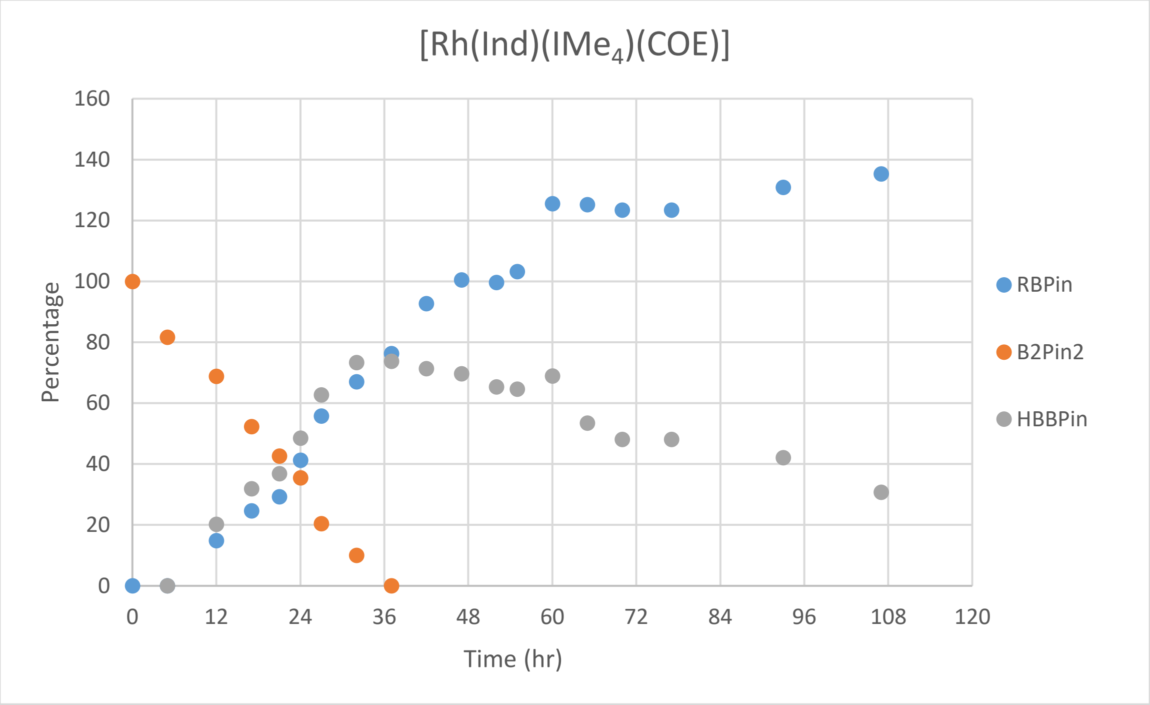

Figure S36. Reaction profile for [Rh(Ind)(IMe$_4$)(COE)] (**6**) as catalyst (5 mol%) for the C-H borylation of benzene with B_2pin_2 (80°C, ferrocene used as internal standard). 100% yield is for the reaction of PhH + B_2pin_2 -> PhBpin + HBpin.

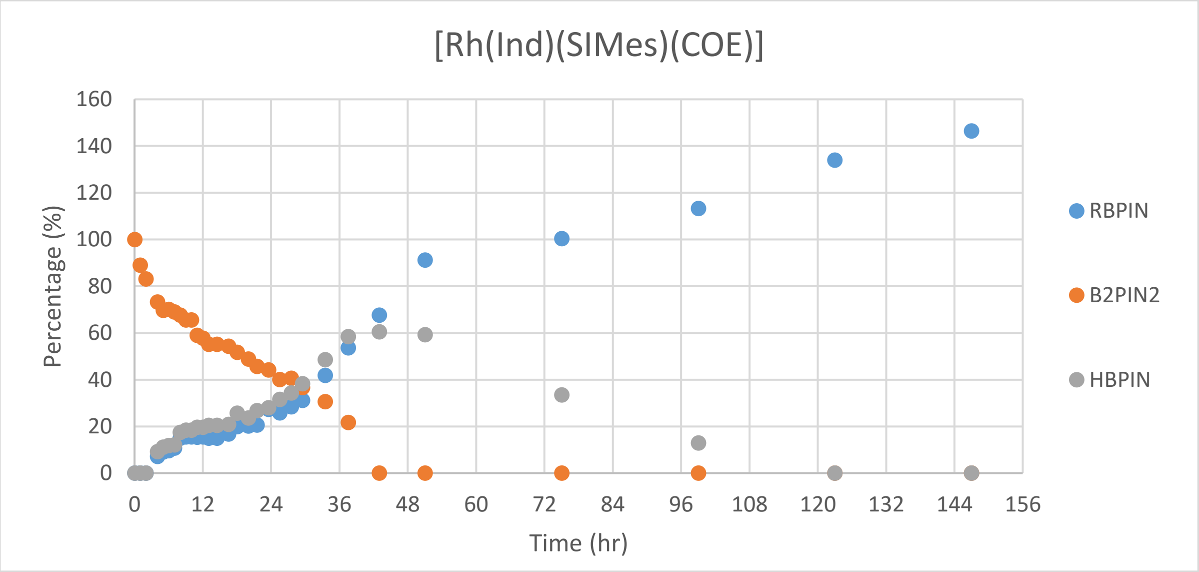

Figure S37. Reaction profile for [Rh(Ind)(SIMes)(COE)] (**10**) as catalyst (5 mol%) for the C-H borylation of benzene with B_2pin_2 (80°C, ferrocene used as internal standard). 100% yield is for the reaction of PhH + B_2pin_2 -> PhBpin + HBpin.

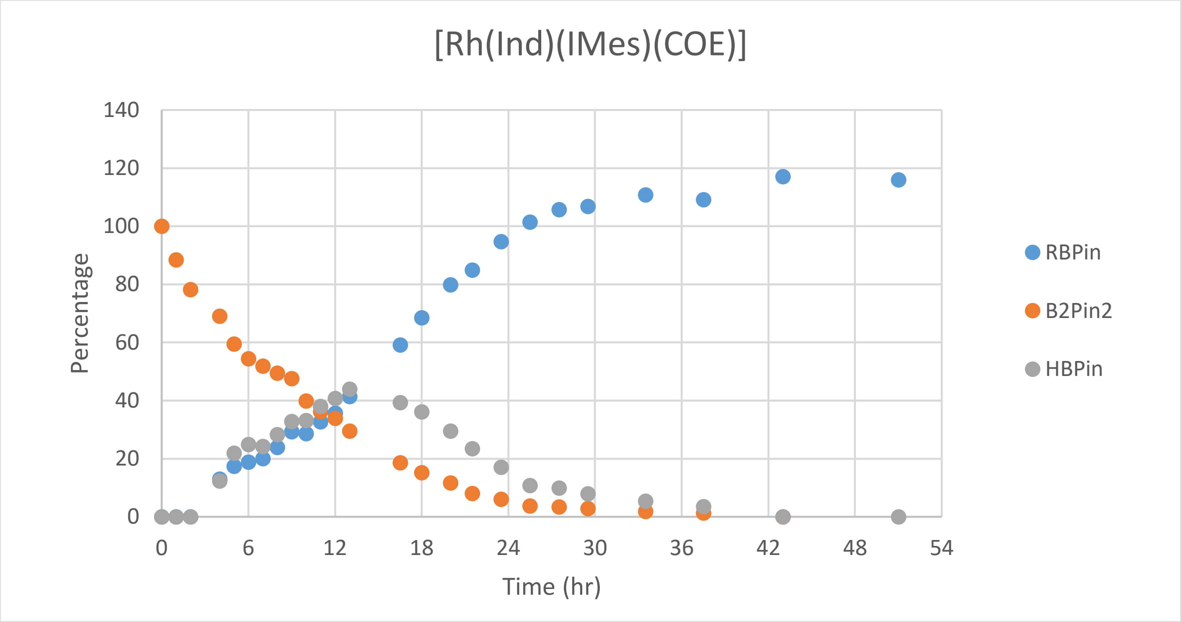

Figure S38. Reaction profile for [Rh(Ind)(IMes)(COE)] (**11**) as catalyst (5 mol%) for the C-H borylation of benzene with B_2pin_2 (80°C, ferrocene used as internal standard). 100% yield is for the reaction of PhH + B_2pin_2 -> PhBpin + HBpin.

NMR-scale reaction of the borylation of benzene with HBpin added

In a glovebox, [Rh(Ind)(IDipp)(COE)] (**4**) (2.5 mg, 3.5 µmol, 5 mol%), ferrocene (internal standard, 1.8 mg, 9.7 µmol), HBpin (6.8 mg, 53 µmol, 76 mol%) and B_2pin_2 (17.7 mg, 70 µmol) were combined in the benzene (3:1 protio:deuterio benzene, 0.7 mL) then added to an NMR tube equipped with a J. Young valve. The sample was then heated in an oil bath at 80°C and the reaction monitored using ^{1}H and ^{11}B NMR spectroscopy.

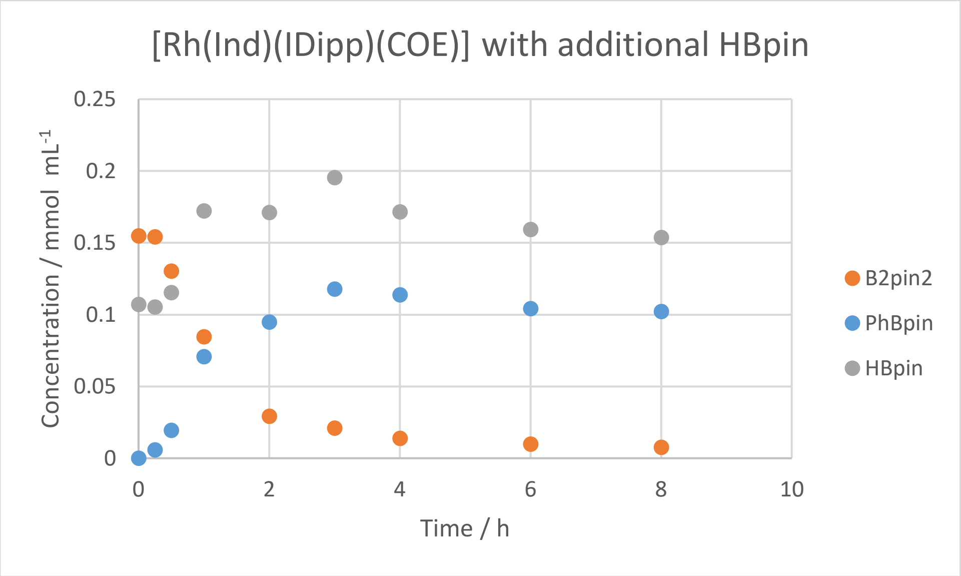

Figure S39. Reaction profile for [Rh(Ind)(IDipp)(COE)] (**4**) as catalyst (5 mol%) for the C-H borylation of benzene with B_2pin_2 with the addition of HBpin to remove the induction period (80°C, ferrocene used as internal standard).

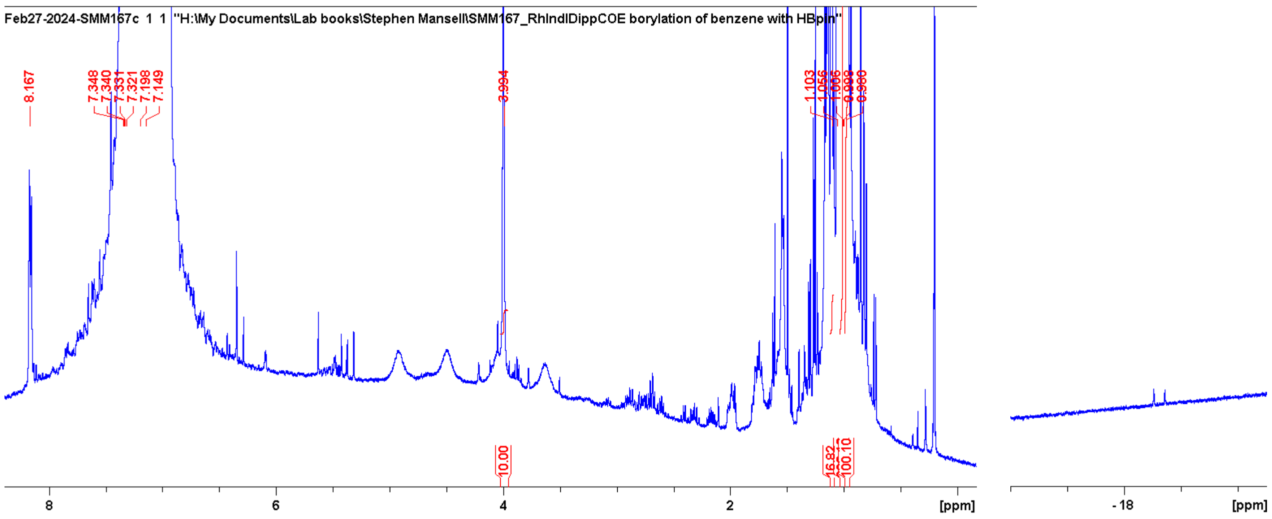

Figure S40. New Rh hydride complex observed after 30 min heating at 80°C in the borylation of C_6H_6/C_6D_6 with B_2pin_2 (100 mol%) and HBpin (76 mol%) using ferrocene as an internal standard. Rh-H resonance: -18.31 ppm, 38.9 Hz.

2.10.4 Decane borylation: NMR spectroscopic monitoring

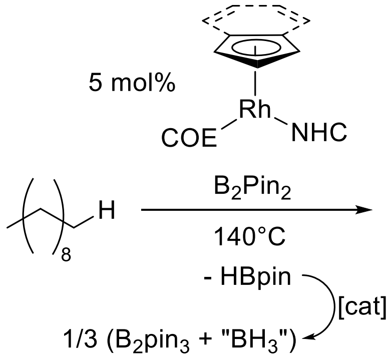

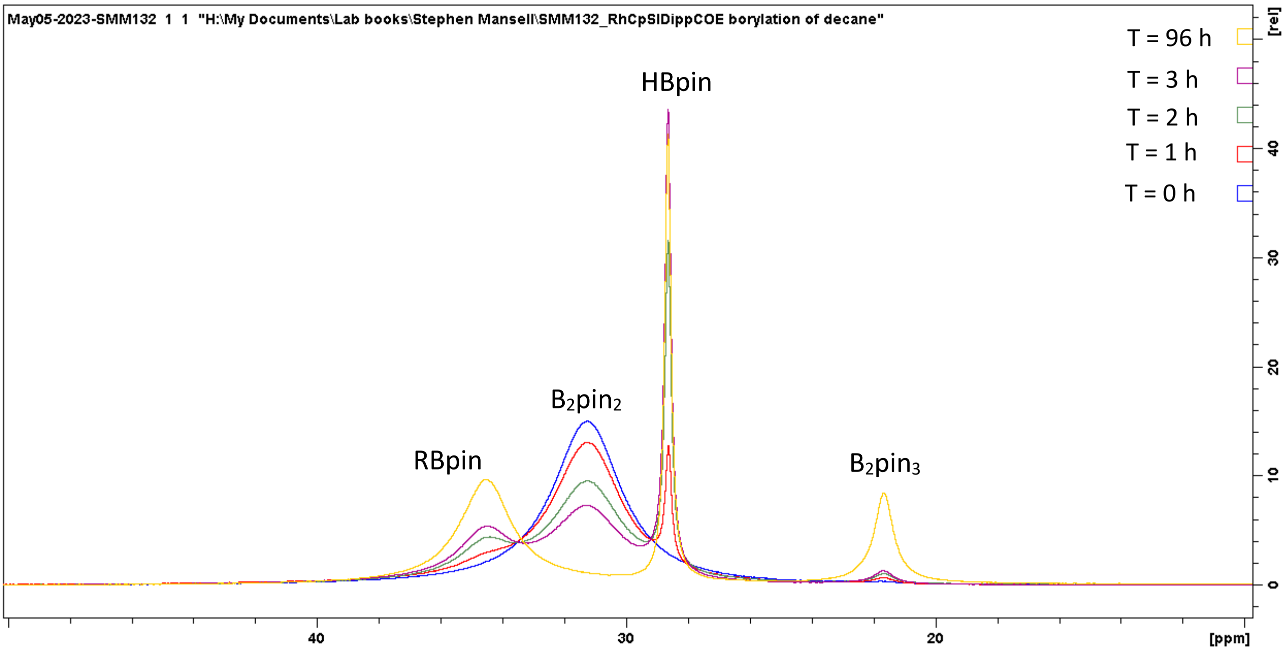

Figure S41. $^{11}B\{^1H\}$ NMR spectra for the attempted borylation of decane using B_2pin_2 and [RhCp(SIDipp)(COE)] (5 mol%).

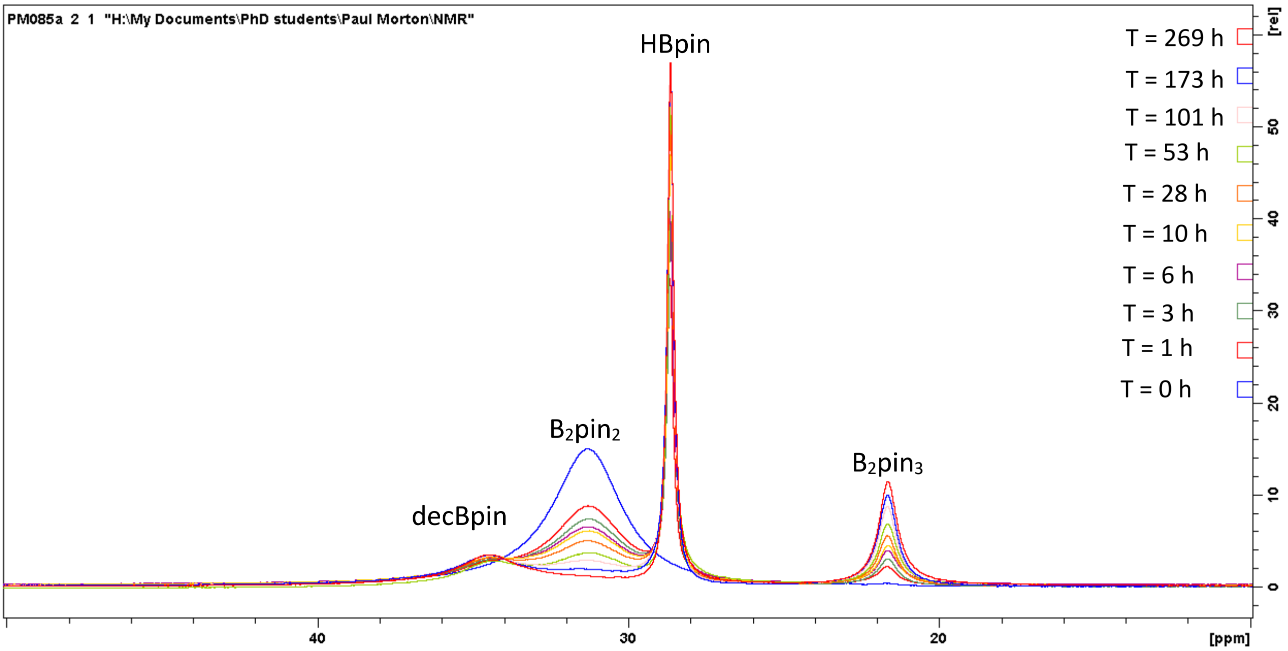

Figure S42. $^{11}B\{^1H\}$ NMR spectra for the borylation of decane using B_2pin_2 and [Rh(Ind)(SIDipp)(COE)] (5 mol%).

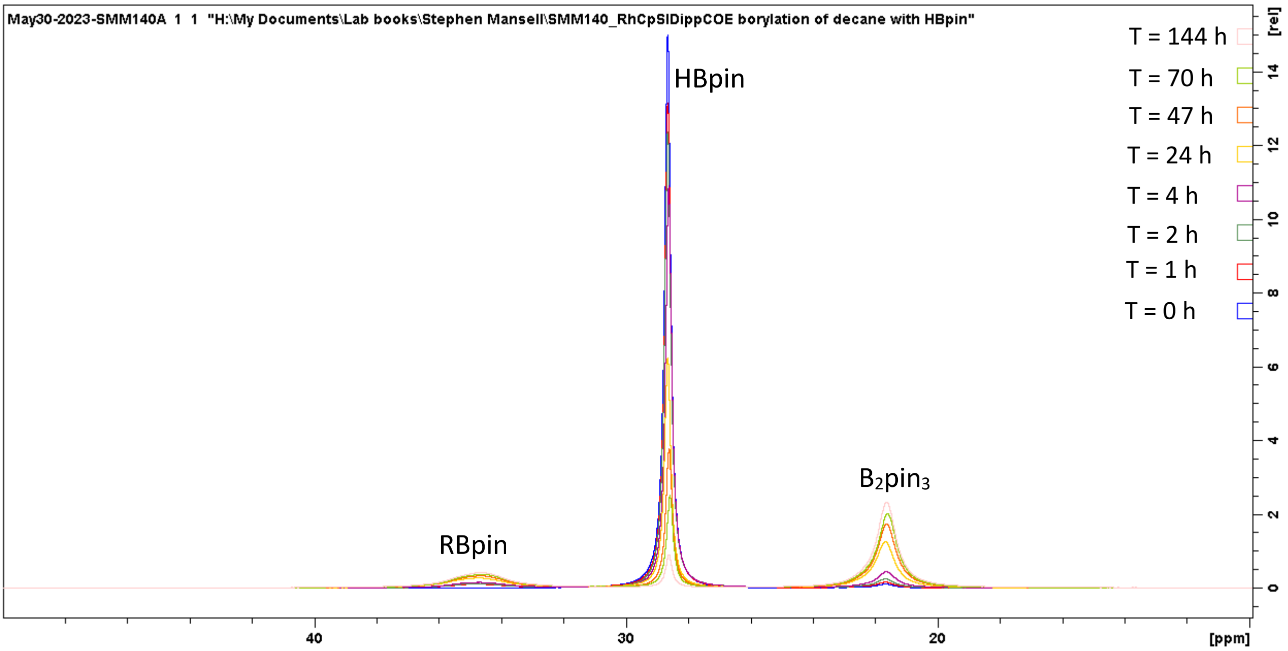

Figure S43. $^{11}B\{^1H\}$ NMR spectra for the attempted borylation of decane using HBpin and [RhCp(SIDipp)(COE)] (5 mol%).

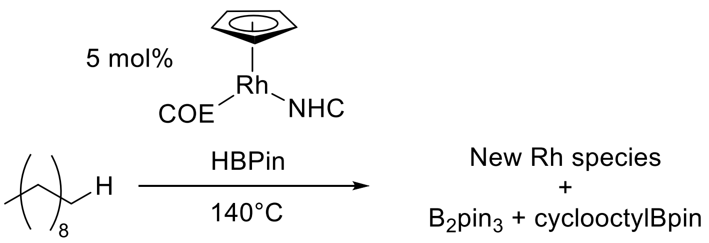

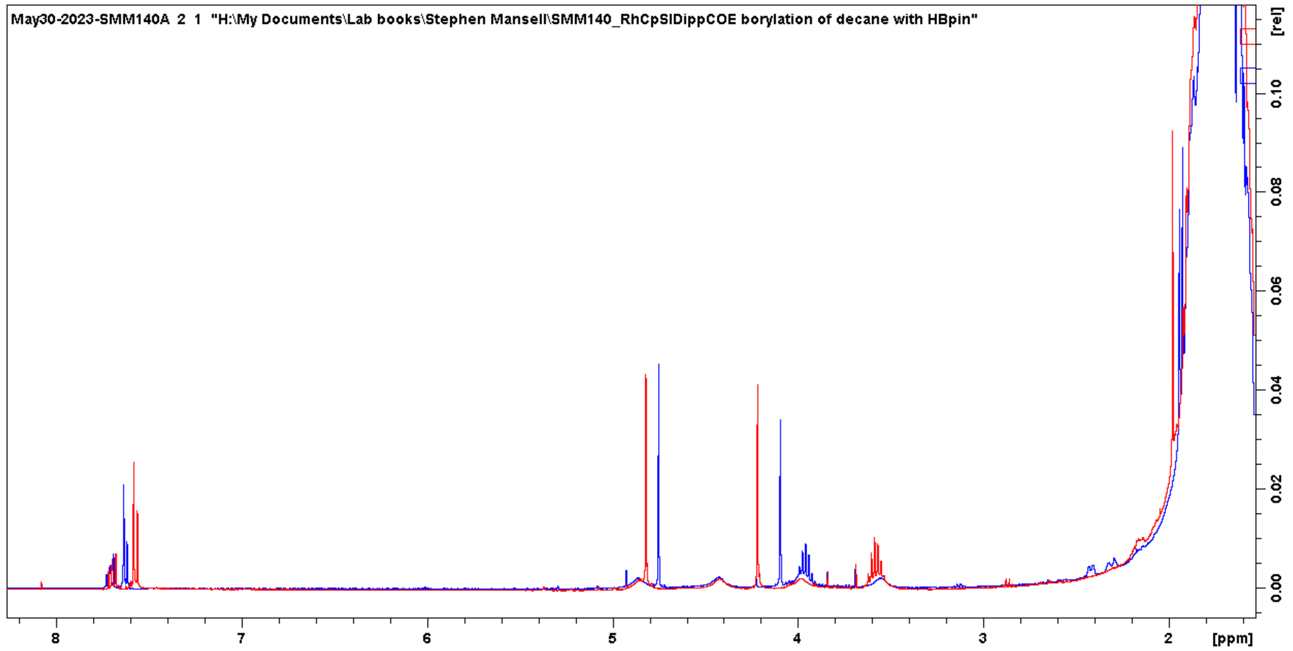

Figure S44. ^{1}H NMR spectroscopic comparison of Rh complexes present at T = 0 (blue) and T = 1 h (red) for the reaction of [RhCp(SIDipp)(COE)] with HBpin at 140°C in decane.

2.10.5 Control reactions

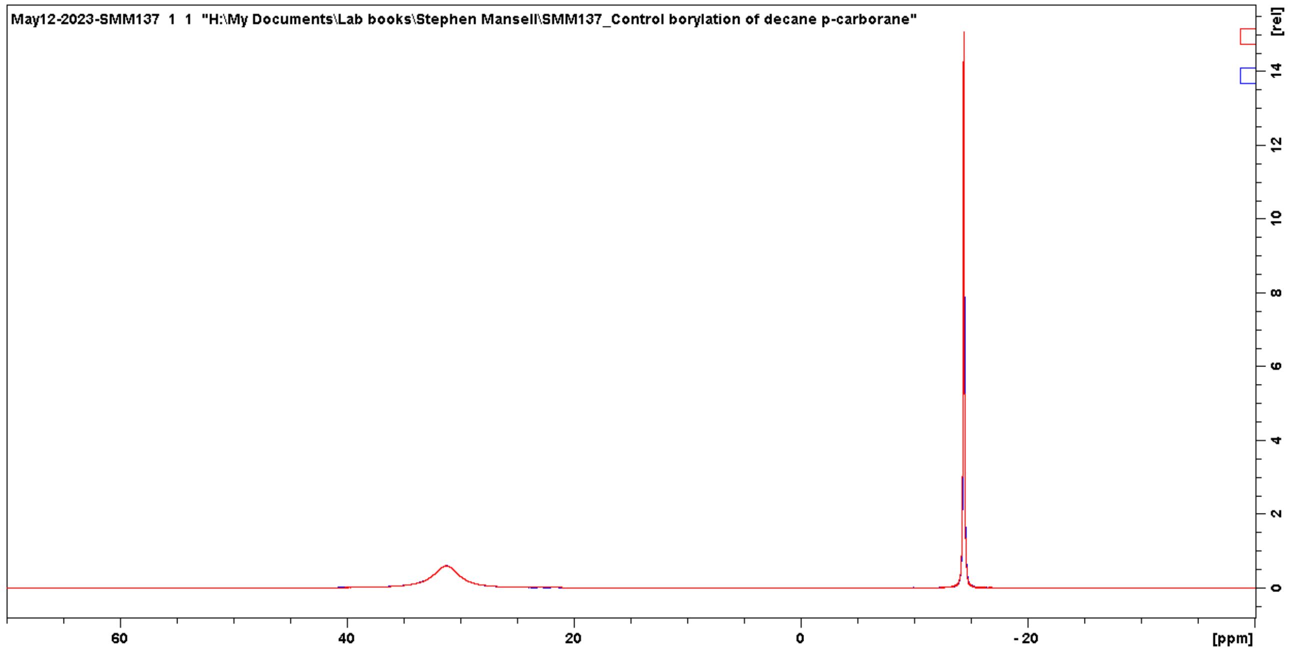

Figure S45. $^{11}B\{^1H\}$ NMR spectra of B_2pin_2 in decane with para-carborane as an internal standard showing no decomposition of B_2pin_2 after 3 d at 140°C.

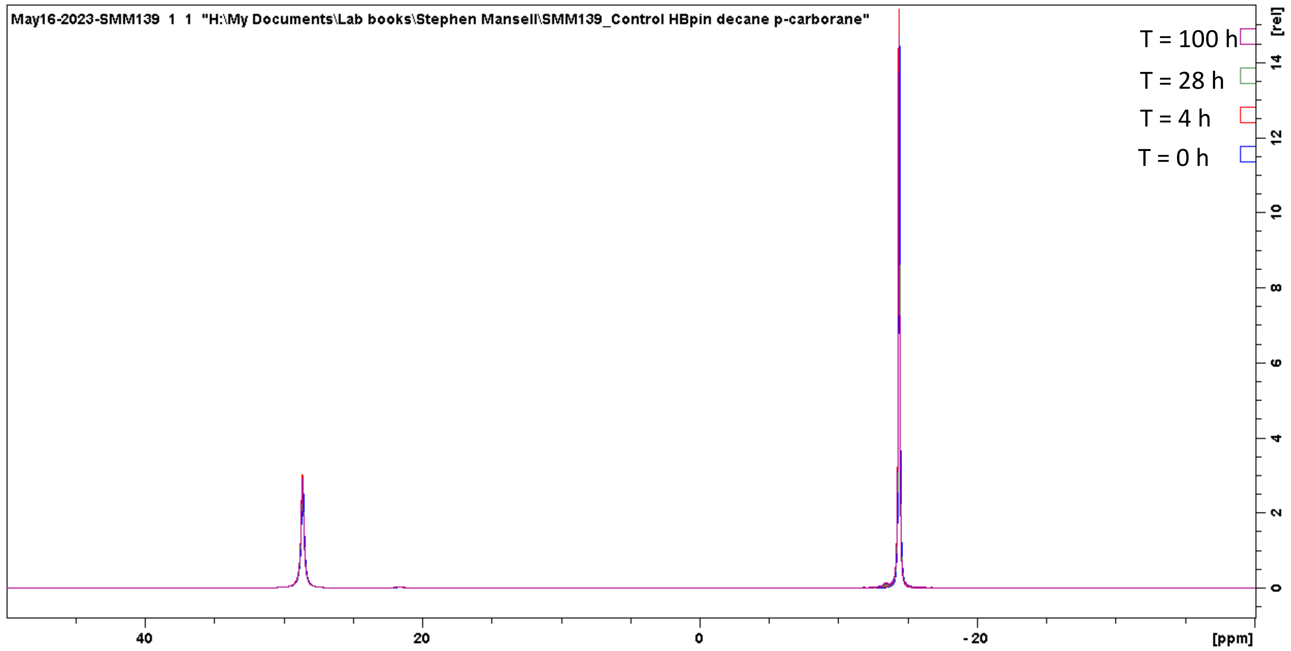

Figure S46. $^{11}B\{^1H\}$ NMR spectra of HBpin in decane with para-carborane as an internal standard showing almost no decomposition of HBpin after 3 d at 140°C. N.B. there is a trace reaction between HBpin and *para*-carborane to give *para*-$B_{10}C_2H_{11}$Bpin.

2.11 Mass spectrometry from C-H borylation reactions

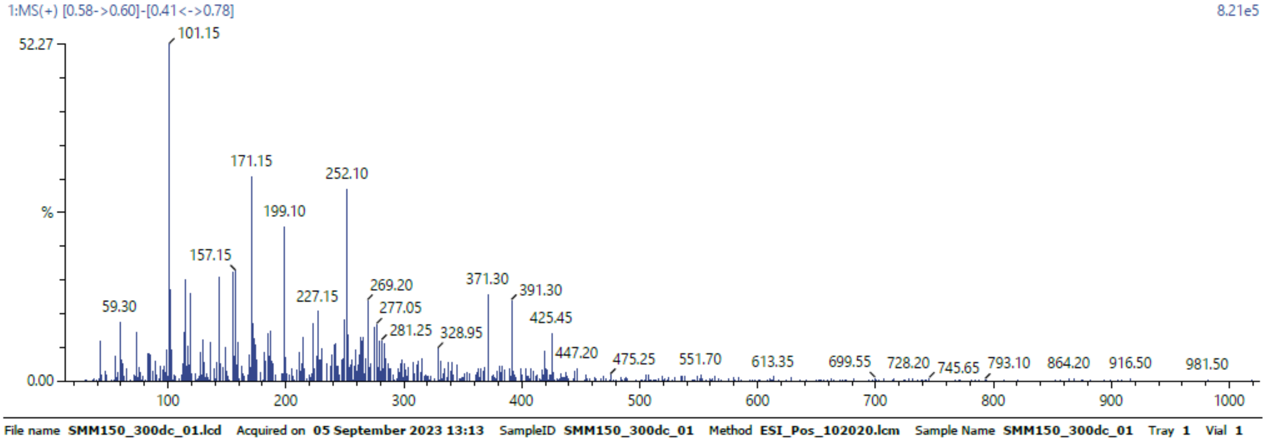

Figure S47. Mass spectrum (ASAP, ESI+ mode) from the reaction of decane with B_2pin_2 catalysed by [RhCp(SIDipp)(COE)]. Peak at 171.15 represents [^{i}PrBpin + H]$^+$; $C_{20}H_{39}B_2O_4^+$.

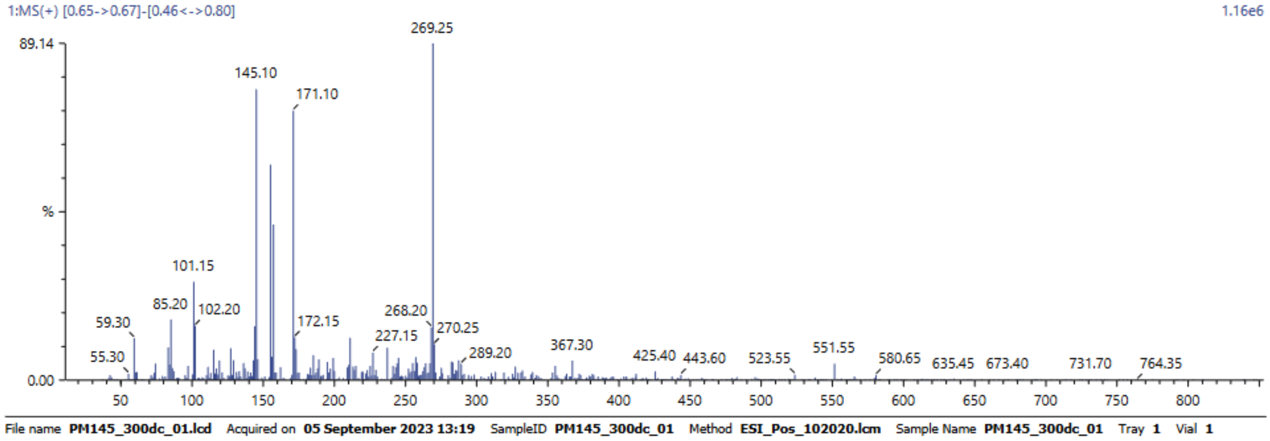

Figure S48. Mass spectrum (ASAP, ESI+ mode) from the reaction of decane with B_2pin_2 catalysed by [Rh(Ind)(IDipp)(COE)]. Peak at 269.25 represents [decBpin + H]$^+$; $C_{16}H_{34}BO_2^+$.

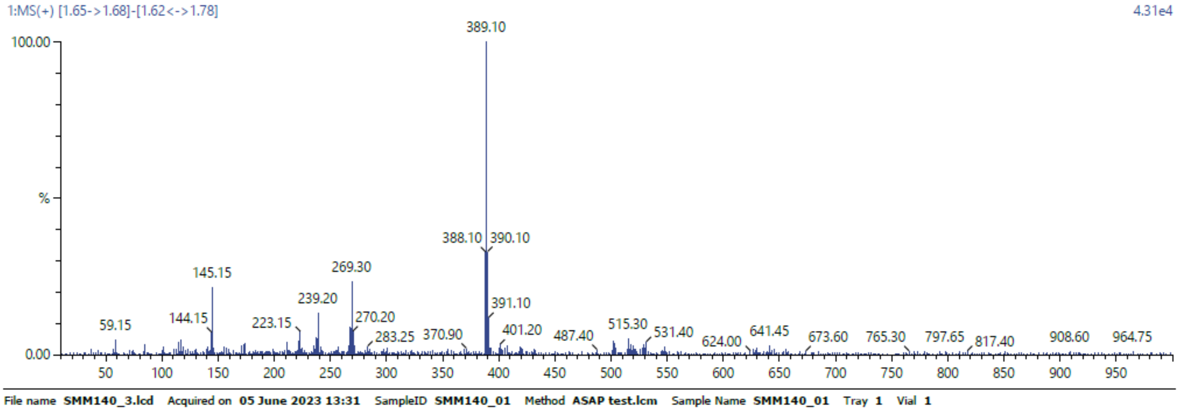

Figure S49. Mass spectrum (ASAP, ESI+ mode) collected from the attempted catalytic borylation of decane using HBpin and 5 mol% [RhCp(SIDipp)(COE)]. [pinBpinBpin+H_3O]$^+$: 389.3; [HOBpin+H]$^+$: 145.17; [pinBOBpin – H]$^+$: 269.17; [cyclooctylBpin+H]$^+$: 239.22.

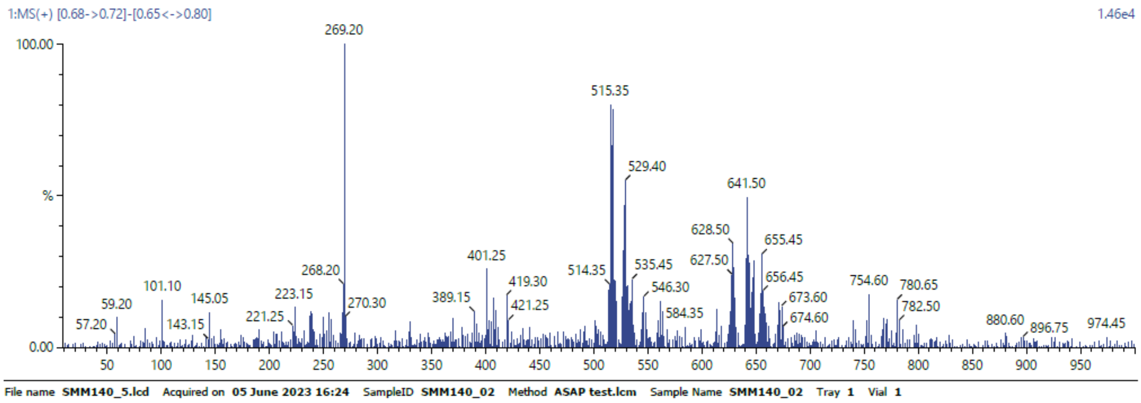

Figure S50. Mass spectrum (ASAP, ESI+ mode, higher probe temperature) collected from the attempted catalytic borylation of decane using HBpin and 5 mol% [RhCp(SIDipp)(COE)]. [pinBOBpin – H]$^+$: 269.17;.[RhCp(NHC')]: 515.19;

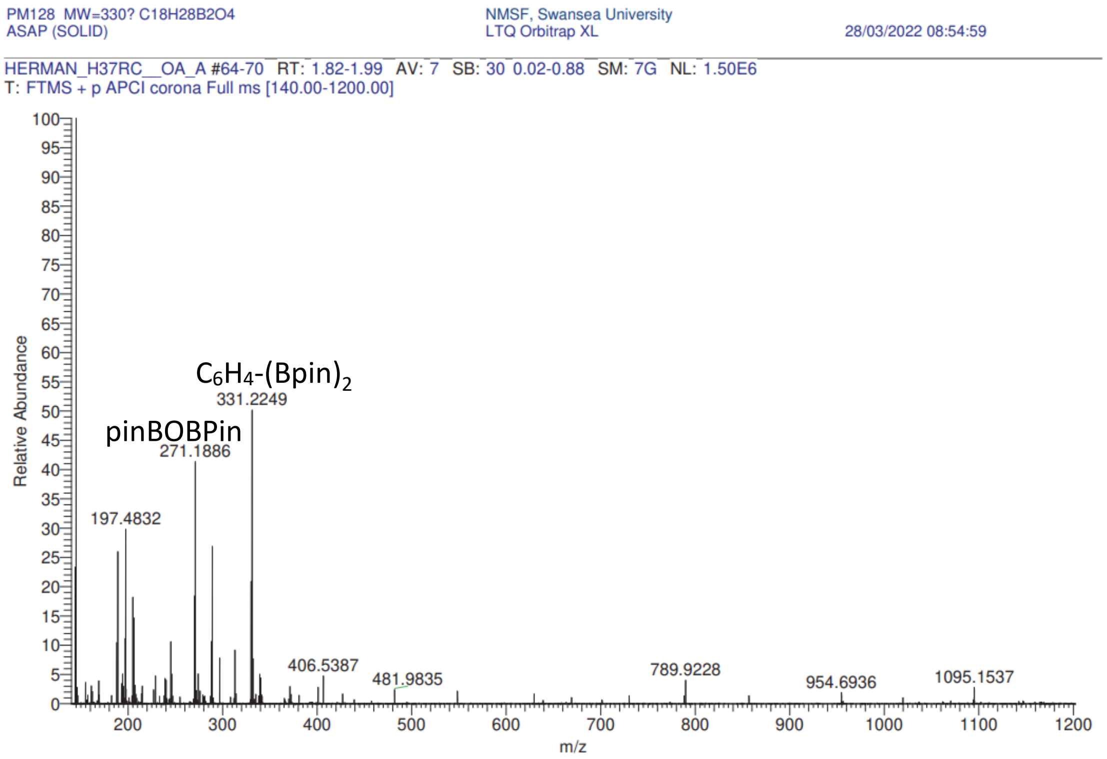

Figure S51. Mass spectrum (ASAP, +ve mode) collected from the product of benzene borylation by 5 mol% [RhCp(IDipp)(COE)] with B_2pin_2. $C_6H_4(Bpin)_2$ was observed ([M+H]$^+$ cald. for $C_{18}H_{29}B_2O_4$: 331.2246) along with pinBOBpin ([M+ H]$^+$ cald. for $C_{12}H_{25}B_2O_5$+: 271.1883).

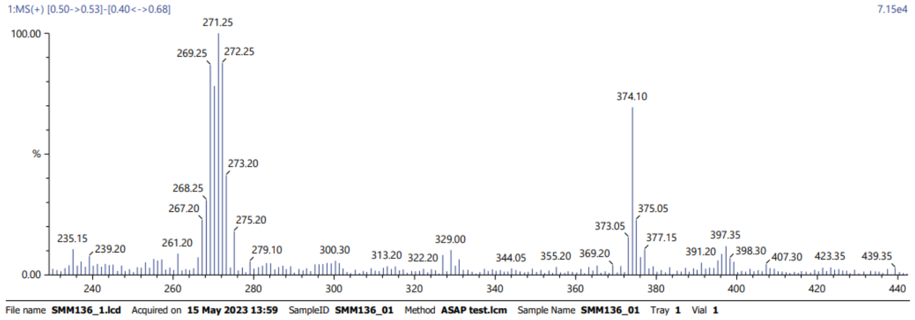

Figure S52. Mass spectrum (ASAP, ESI+ mode) of monoborylated para-carborane. Expected for [pinB-$C_2B_{10}H_{11}$+H]$^+$: 271.29.

3 X-ray crystallography

3.1 Crystallographic details

Single crystals suitable for X-ray diffraction were covered in inert oil and placed under the cold stream of a Bruker D8 Venture diffractometer at 100 K. Exposures were collected using Mo-K_{α} radiation (λ =0.71073) or Cu- K_{α} radiation (λ = 1.54178). Indexing, data collection and absorption corrections were performed. The structures were then solved using SHELXT[13] and refined by full-matrix least-squares refinement (SHELXL)[13] interfaced with the programme OLEX2.[14]

One of the ethoxide groups in [RhCp(H){Si(OEt)$_3$}(SIDipp)] was found to be disordered and was modelled over two positions in a ratio of 0.67:0.33. There was extensive disordered in the tetrahydrofluorenyl structure which was modelled over several positions. CCDC deposition numbers: 2294822-2294829.

3.2 Structures of additional monodentate complexes

3.2.1 [Rh(Ind)(IDipp)(COE)] (4)

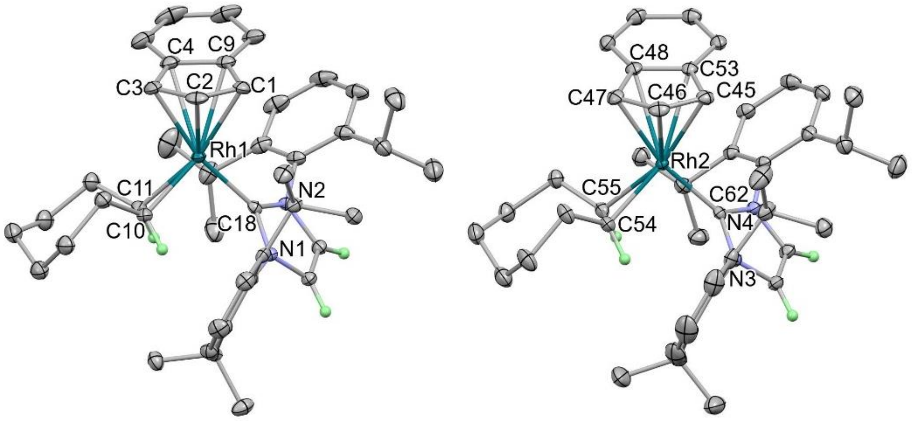

Figure S53. Comparison of the two molecules in the asymmetric unit for [Rh(Ind)(IDipp)(COE)] (**4**) with thermal ellipsoids at 50% probability. All H atoms except for those on the NHC backbone and the alkene have been removed for clarity.

3.2.2 [Rh(Ind)(IDipp)(CO)] (5)

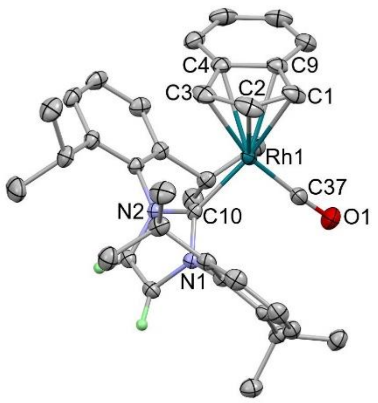

Figure S54. Molecular structure of [Rh(Ind)(IDipp)(CO)] (**5**). Thermal ellipsoids at 50% probability and all H atoms, except those on the NHC backbone, have been removed for clarity.

3.2.3 A new polymorph of [Rh(Ind)(SIMes)(COE)]

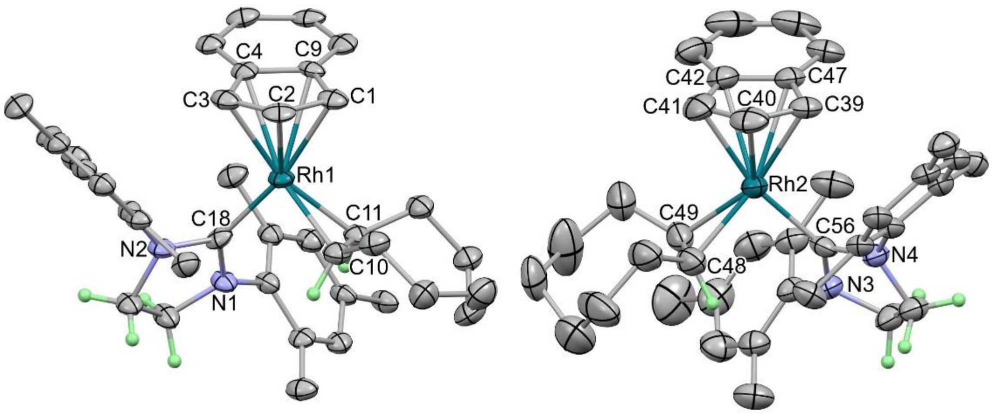

Figure S55. Comparison of the two molecules in the asymmetric unit for [Rh(Ind)(SIMes)(COE)] (**10**) with thermal ellipsoids at 50% probability. All H atoms except for those on the NHC backbone and the alkene have been removed for clarity. The second position of the disordered COE ring in the molecule featuring Rh2 has also been omitted for clarity.

3.2.4 [Rh(Ind)(IMe$_4$)$_2$] (6)

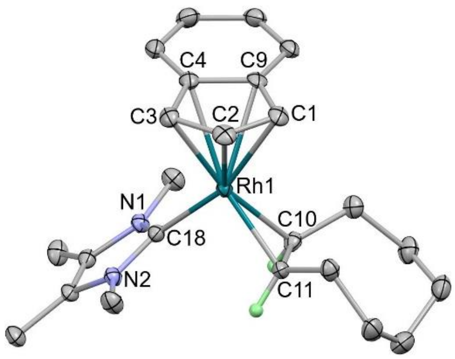

Figure S56. Molecular structure of [Rh(Ind)(IMe$_4$)(COE)] (**6**). Thermal ellipsoids at 50% probability and all H atoms, except those on the alkene, have been removed for clarity.

3.2.5 [Rh(Flu-H$_4$)(COE)$_2$] (8)

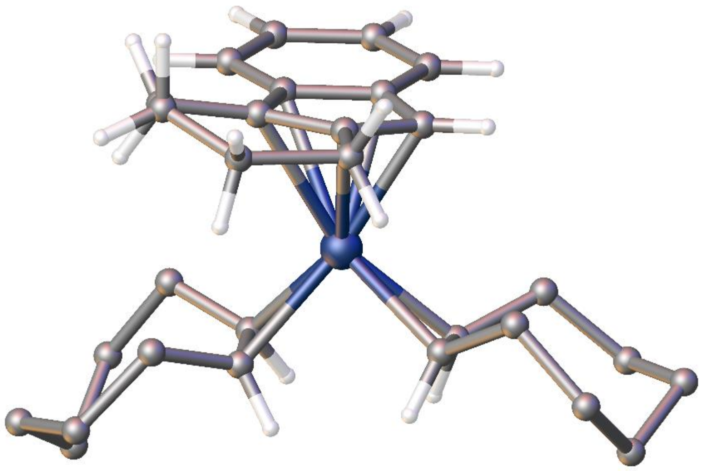

Figure S57. Molecular structure of [Rh(Flu-H$_4$)(COE)$_2$] (**8**) depicted as a ball-and-stick picture. There was disorder present in all the ligands, and only one position of each has been shown for clarity.

3.3 Comparsion of bond lengths and angles for Rh complexes

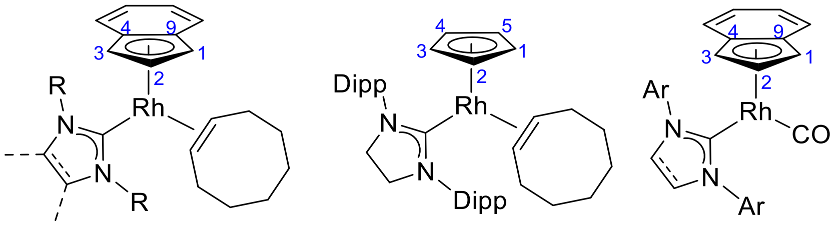

Table S1. Comparison of bond lengths (Å) for the piano-stool rhodium complexes. Fold angle is calculated from a place generated from the benzannulated ring and the plane formed from C1, C2, C3.

**Compound**	**C1**	**C2**	**C3**	**C4**	**C9**	**Fold angle /°**	**Rh-NHC**	**M-C$_{COE}$**		**C=C (COE)**
Rh(Ind)(COE)$_2$ [7]	2.230(2)	2.245(2)	2.2385(19)	2.4148(17)	2.4126(18)	9.0		2.150(2)<br>2.152(2)	2.151(2)<br>2.166(2)	1.405(3)<br>1.408(3)
Rh(Ind)(SIDipp)(COE) **9** [15] **A**	2.2955(16)	2.2034(16)	2.2310(16)	2.5277(16)	2.5674(16)	10.2	1.9881(15)	2.1161(16)	2.1401(16)	1.409(2)
Molecule **B**	2.2803(17)	2.1968(17)	2.2271(18)	2.5572(17)	2.5806(17)	12.4	1.9946(15)	2.1376(17)	2.1370(17)	1.407(2)
Rh(Ind)(IDipp)(COE) **4** **A**	2.2434(17)	2.1956(17)	2.2551(17)	2.5352(18)	2.5397(18)	12.3	2.0179(17)	2.1165(17)	2.1520(17)	1.410(3)
Molecule **B**	2.2318(16)	2.1960(17)	2.2486(17)	2.5483(17)	2.5326(17)	12.6	2.0154(17)	2.1175(16)	2.1516(16)	1.415(2)
Rh(Ind)(SIMes)(COE) **A**	2.232(5)	2.241(4)	2.291(5)	2.445(5)	2.437(4)	8.1	2.003(5)	2.112(5)	2.121(5)	1.423(7)
(New polymorph) Mol **B**	2.251(5)	2.228(6)	2.241(6)	2.446(5)	2.454(5)	10.0	2.015(5)	2.124(6)	2.139(6)	1.405(10)
Rh(Ind)(IMe$_4$)(COE) **6**	2.240(2)	2.2326(19)	2.2501(19)	2.3892(19)	2.3815(19)	5.5	1.9985(19)	2.0899(19)	2.1141(19)	1.419(3)
**Compound**	**C1**	**C2**	**C3**	**C4**	**C5**		**Rh-NHC**	**M-C$_{COE}$**		**C=C (COE)**
RhCp(SIDipp)(COE) **1**	2.278(2)	2.238(2)	2.319(2)	2.333(2)	2.273(2)		1.9935(19)	2.1233(19)	2.1230(19)	1.419(3)
RhCp(cyclometallated NHC)(iPr) **rac-2**	2.320(2)	2.302(2)	2.270(2)	2.267(2)	2.290(2)		1.939(2)	Rh-Ar: 2.004(2)	Rh-^{i}Pr: 2.150(2)	

Compound	C1	C2	C3	C4	C5		Rh-NHC	Rh-Si	Rh-H	H…Si
Rh(Cp){Si(OEt)$_3$}(H)(SIDipp) **rac-3**	2.2710(15)	2.2261(15)	2.2901(15)	2.3360(16)	2.3394(16)		1.9849(14)	2.2758(4)	1.412	2.392
**Compound**	**C1**	**C2**	**C3**	**C4**	**C9**	**Fold angle /°**	**Rh-NHC**	**Rh-Si**	**Rh-H**	**H…Si**
Rh(Ind){Si(OEt)$_3$}(H)(SIDipp) [15]	2.340(3)	2.248(3)	2.220(3)	2.462(3)	2.552(3)	8.8	2.014(3)	2.2691(8)	1.459	2.239
**Compound**	**C1**	**C2**	**C3**	**C4**	**C9**		**Rh-NHC**	**M-C$_{CO}$**		**C≡O**
Rh(Ind)(SIDipp)(CO) [15]	2.2485(17)	2.2209(19)	2.2358(18)	2.4854(16)	2.4762(16)	9.7	2.0103(14)	1.8277(19)		1.154(2)
Rh(Ind)(IDipp)(CO) **5**	2.2480(18)	2.2419(19)	2.2404(18)	2.4531(17)	2.4334(17)	7.5	2.0187(16)	1.822(2)		1.157(2)

3.4 Crystallographic tables of data

Table S2. Additional crystallographic data.

	[RhCp(SIDipp)(COE)] (**1**)	[RhCp(^{i}Pr)(SIDipp')] (***rac*-2**)	[RhCp(H){Si(OEt)$_3$}(SIDipp)] (***rac*-3**)
Empirical formula	$C_{40}H_{57}N_2Rh$	$C_{35}H_{50}N_2Rh$	$C_{38}H_{59}N_2O_3RhSi$
Formula weight	668.78	601.68	722.87
T/K	100.0	100.0	100.0
Crystal system	monoclinic	monoclinic	triclinic
Space group	P2$_1$/*c*	P2$_1$/*c*	P-1
a/Å	10.3232(3)	11.27470(10)	9.7812(3)
b/Å	16.9673(5)	16.1877(2)	11.2038(4)
c/Å	19.7805(6)	16.8479(2)	17.9863(6)
α/°	90	90	94.8030(10)
β/°	98.3910(10)	93.1180(10)	93.8310(10)
γ/°	90	90	108.8330(10)
Volume/Å^3	3427.60(18)	3070.38(6)	1849.76(11)
Z	4	4	2
ρ_{calc}g/cm^3	1.296	1.302	1.298
μ/mm^1	4.234	4.667	4.323
F(000)	1424.0	1276.0	768.0
Crystal size/mm^3	0.2 × 0.17 × 0.04	0.18 × 0.14 × 0.04	0.467 × 0.404 × 0.218
Radiation	CuK$_\alpha$ (λ = 1.54178)	CuK$_\alpha$ (λ = 1.54178)	CuK$_\alpha$ (λ = 1.54178)
2Θ range for data collection/°	6.896 to 159.956	7.578 to 144.342	4.954 to 144.148
Index ranges	-13 ≤ h ≤ 12, -21 ≤ k ≤ 21, -25 ≤ l ≤ 24	-13 ≤ h ≤ 12, -19 ≤ k ≤ 18, -20 ≤ l ≤ 20	-12 ≤ h ≤ 12, -13 ≤ k ≤ 13, -22 ≤ l ≤ 21
Reflections collected	95197	21150	60337
Independent reflections	7391 [R_{int} = 0.0451, R_{sigma} = 0.0203]	5982 [R_{int} = 0.0352, R_{sigma} = 0.0316]	7231 [R_{int} = 0.0376, R_{sigma} = 0.0201]
Data/ restraints/ parameters	7391/0/396	5982/0/352	7231/2/440
Goodness-of-fit on F^2	1.063	1.046	1.068
Final R indexes [I>=2σ (I)]	R_1 = 0.0287, $_wR_2$ = 0.0759	R_1 = 0.0273, $_wR_2$ = 0.0711	R_1 = 0.0204, $_wR_2$ = 0.0542
Final R indexes [all data]	R_1 = 0.0302, $_wR_2$ = 0.0767	R_1 = 0.0307, $_wR_2$ = 0.0729	R_1 = 0.0217, $_wR_2$ = 0.0543
Largest diff. peak/hole (e Å^{-3})	0.45/-0.67	0.83/-0.45	0.52/-0.62
CSD deposition numbers	2294822	2294823	2294824

Table S2 continued. Additional crystallographic data.

	[Rh(Ind)(IDipp)(COE)] (**4**)	[Rh(Ind)(IDipp)(CO)] (**5**)	[Rh(Ind)(SIMes)(COE)] New polymorph of **10**
Empirical formula	$C_{44}H_{57}N_2Rh$	$C_{37}H_{43}N_2ORh$	$C_{38}H_{47}N_2Rh$
Formula weight	716.82	634.64	634.68
T/K	101.0	100.0	100.0
Crystal system	monoclinic	monoclinic	triclinic
Space group	$P2_1/c$	$P2_1/n$	*P*-1
a/Å	18.8278(4)	9.9726(3)	10.9441(16)
b/Å	39.0600(8)	19.2837(6)	11.4368(16)
c/Å	10.5156(2)	16.8847(5)	28.391(5)
α/°	90	90	96.471(7)
β/°	104.3800(10)	104.9246(13)	92.253(8)
γ/°	90	90	94.806(6)
Volume/Å^3	7491.0(3)	3137.53(17)	3514.3(9)
Z	8	4	4
ρ_{calc}g/cm^3	1.271	1.344	1.200
μ/mm^1	3.913	4.628	4.108
F(000)	3040.0	1328.0	1336.0
Crystal size/mm^3	0.36 × 0.2 × 0.16	0.26 × 0.2 × 0.08	0.22 × 0.04 × 0.02
Radiation	CuKα (λ = 1.54178)	CuKα (λ = 1.54178)	CuKα (λ = 1.54178)
2Θ range for data collection/°	4.524 to 144.206	7.096 to 149.496	6.274 to 131.192
Index ranges	-23 ≤ h ≤ 22, -48 ≤ k ≤ 48, -12 ≤ l ≤ 12	-12 ≤ h ≤ 11, -24 ≤ k ≤ 23, -21 ≤ l ≤ 20	-12 ≤ h ≤ 12, -13 ≤ k ≤ 13, -33 ≤ l ≤ 33
Reflections collected	79689	70918	68959
Independent reflections	14648 [R_{int} = 0.0375, R_{sigma} = 0.0251]	6402 [R_{int} = 0.0408, R_{sigma} = 0.0196]	12028 [R_{int} = 0.0958, R_{sigma} = 0.0626]
Data/ restraints/ parameters	14648/0/863	6402/0/378	12028/34/773
Goodness-of-fit on F^2	1.085	1.046	1.035
Final R indexes [I>=2σ (I)]	R_1 = 0.0259, wR_2 = 0.0640	R_1 = 0.0253, wR_2 = 0.0671	R_1 = 0.0566, wR_2 = 0.1339
Final R indexes [all data]	R_1 = 0.0272, wR_2 = 0.0645	R_1 = 0.0274, wR_2 = 0.0685	R_1 = 0.0757, wR_2 = 0.1441
Largest diff. peak/hole (e Å^{-3})	0.59/-0.56	0.58/-1.03	0.80/-1.50
CSD deposition numbers	2294825	2294826	2294829

Table S2 continued. Additional crystallographic data.

	[Rh(Ind)(IMe$_4$)(COE)] (**6**)	[Rh(Flu-H$_4$)(COE)$_2$] (**8**)
Empirical formula	$C_{24}H_{33}N_2Rh$	$C_{29}H_{41}Rh$
Formula weight	452.43	492.53
T/K	100.0	100.0
Crystal system	triclinic	monoclinic
Space group	*P*-1	*P*2$_1$/*n*
a/Å	9.4639(10)	11.7294(3)
b/Å	10.0697(13)	10.8565(2)
c/Å	11.4186(13)	18.6200(5)
α/°	82.698(6)	90
β/°	89.424(5)	92.0116(11)
γ/°	77.113(4)	90
Volume/Å^3	1052.0(2)	2369.61(10)
Z	2	4
ρ_{calc}g/cm^3	1.428	1.381
μ/mm^1	6.621	0.734
F(000)	472.0	1040.0
Crystal size/mm^3	0.12 × 0.06 × 0.03	0.24 × 0.18 × 0.06
Radiation	CuKα (λ = 1.54178)	MoKα (λ = 0.71073)
2Θ range for data collection/°	7.806 to 149.634	5.114 to 61.062
Index ranges	-11 ≤ h ≤ 11, -11 ≤ k ≤ 12, -14 ≤ l ≤ 14	-16 ≤ h ≤ 16, -15 ≤ k ≤ 15, -26 ≤ l ≤ 26
Reflections collected	33663	79272
Independent reflections	4313 [R_{int} = 0.0482, R_{sigma} = 0.0261]	7242 [R_{int} = 0.0563, R_{sigma} = 0.0286]
Data/ restraints/ parameters	4313/0/248	7242/9/300
Goodness-of-fit on F^2	1.074	1.057
Final R indexes [I>=2σ (I)]	R_1 = 0.0227, wR_2 = 0.0582	R_1 = 0.0371, wR_2 = 0.0817
Final R indexes [all data]	R_1 = 0.0243, wR_2 = 0.0589	R_1 = 0.0523, wR_2 = 0.0898
Largest diff. peak/hole (e Å^{-3})	0.94/-0.49	1.44/-0.96
CSD deposition numbers	2294827	2294828

4 High resolution mass spectrometry data

N.B. ChemDraw structures are indicative only of MW, not structure.

4.1 Rh complexes

4.1.1 [Rh(Ind)(IDipp)(CO)] (4)

HRMS (ASAP/TOF): Calcd. for $[C_{37}H_{44}N_2O^{103}Rh]^+$: 635.2503 $[M+H]^+$, Found: 635.2501 m/z.

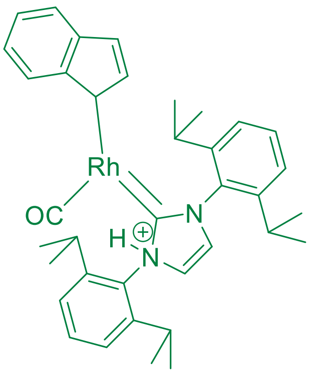

Chemical Formula: $C_{37}H_{44}N_2ORh^+$
Exact Mass: 635.2503
Molecular Weight: 635.6770

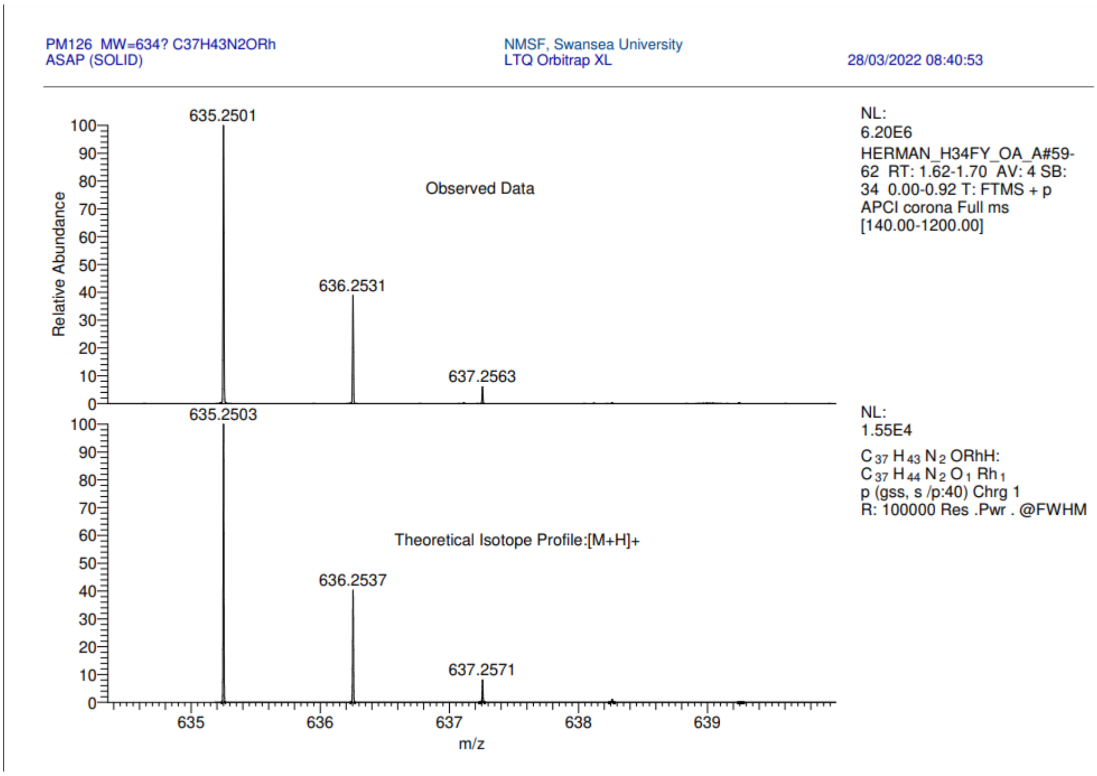

Figure S58. High resolution mass spectrum of **4**.

4.1.2 [Rh(Ind)(IMe₄)(COE)] (6)

HRMS (ASAP/TOF): Calcd. for $C_{24}H_{34}N_2{}^{103}Rh^+$: 453.1772, $[M+H]^+$, Found: 453.1775 m/z.

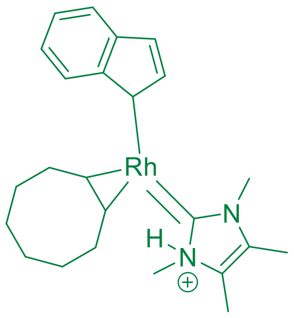

Chemical Formula: $C_{24}H_{34}N_2Rh^+$
Exact Mass: 453.1772
Molecular Weight: 453.4550

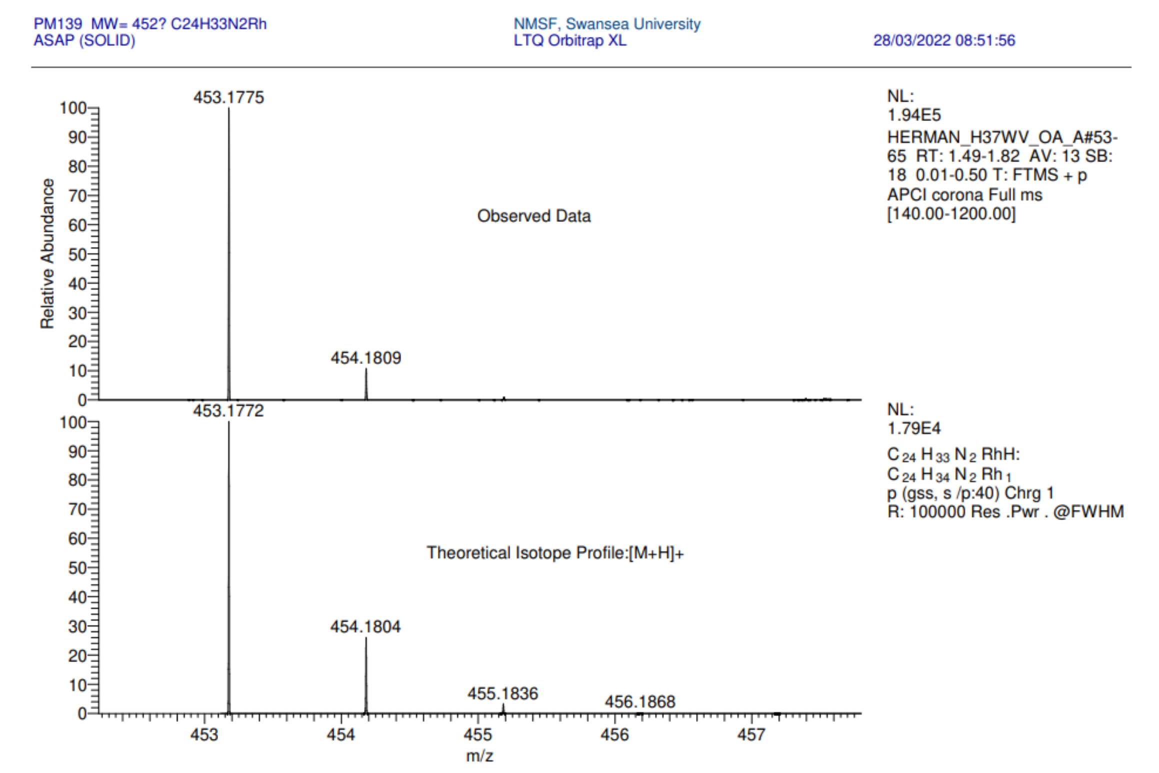

Figure S59. High resolution mass spectrum of **6**.

4.1.3 [Rh(Flu-H$_4$)(COE)$_2$] (8)

Mass spectrometry revealed fragments. **HRMS (ASAP/TOF)**: Calcd. for [$C_{21}H_{26}{}^{103}Rh_2$]$^+$: 381.1089 [Rh(Ind)(C_8H_{13})]$^+$, Found: m/z. 381.1091.

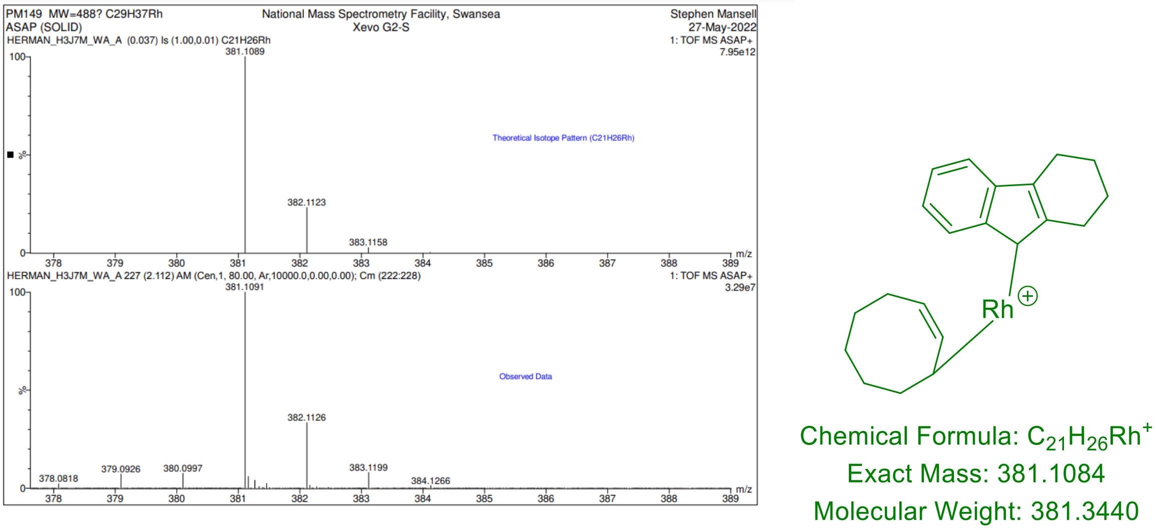

HRMS (ASAP/TOF): Calcd. for [$C_{26}H_{26}{}^{103}Rh$]$^+$: 441.1089 [Rh(Flu-H$_4$)$_2$]$^+$, Found: m/z. 441.1084.

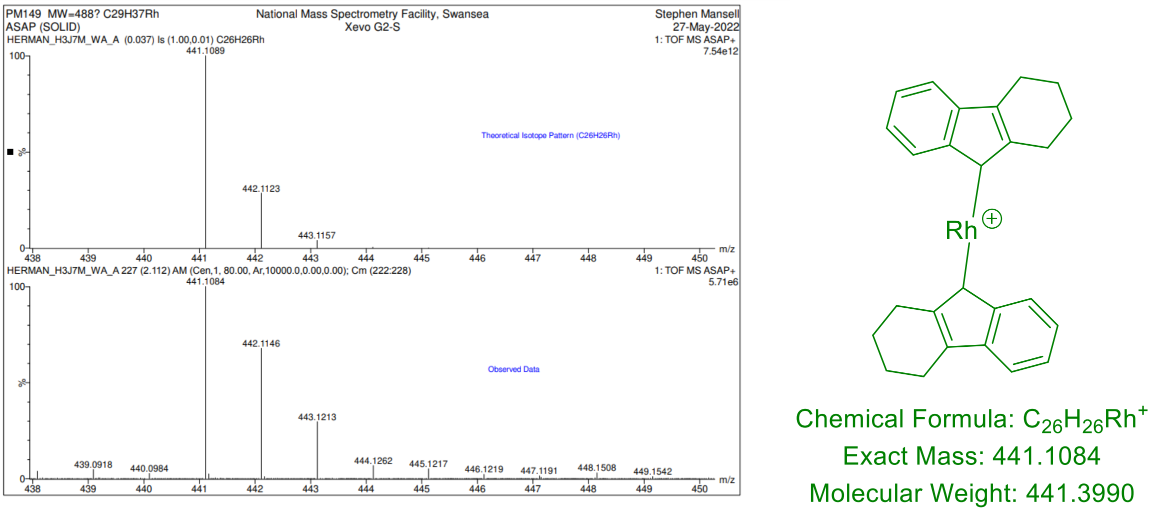

Figure S60. High resolution mass spectra of species derived from samples of **8**.

5 Computational Details.

All calculations were performed using Gaussian 16 (Revision A.03)[C1] and employed the BP86 functional.[C2, C3] Rh atoms were described with a Stuttgart RECP and the corresponding basis set[C4] while 6-31G** basis sets were used to describe all other atoms.[C5] Analytical frequency calculations were carried out to confirm minima as having no imaginary frequencies, while transition states all presented one negative frequency. The latter were verified by running IRCs and subsequent optimizations to connect them to the adjacent minima. Electronic energies were corrected for benzene solvation (PCM method[C6] using the basis set described above), dispersion (BJD3[C7]) and basis set effects by recomputing with the Def2-TZVP basis set[C8] on all atoms. These corrections were combined with the thermochemical corrections taken from the BP86-optimised frequency calculations to give the free energies quoted in the text. Cartesian coordinates and computed energies of all stationary points are provided in Section 6 and in a separate xyz file that can be viewed with (e.g.) Chemcraft[C9] or Mercury.[C10]

5.1 Computational References

C1 M. J. Frisch, G. W. Trucks, H. B. Schlegel, G. E. Scuseria, M. A. Robb, J. R. Cheeseman, G. Scalmani, V. Barone, G. A. Petersson, H. Nakatsuji, X. Li, M. Caricato, A. V. Marenich, J. Bloino, B. G. Janesko, R. Gomperts, B. Mennucci, H. P. Hratchian, J. V. Ortiz, A. F. Izmaylov, J. L. Sonnenberg, D. Williams-Young, F. Ding, F. Lipparini, F. Egidi, J. Goings, B. Peng, A. Petrone, T. Henderson, D. Ranasinghe, V. G. Zakrzewski, J. Gao, N. Rega, G. Zheng, W. Liang, M. Hada, M. Ehara, K. Toyota, R. Fukuda, J. Hasegawa, M. Ishida, T. Nakajima, Y. Honda, O. Kitao, H. Nakai, T. Vreven, K. Throssell, J. J. A. Montgomery, J. E. Peralta, F. Ogliaro, M. J. Bearpark, J. J. Heyd, E. N. Brothers, K. N. Kudin, V. N. Staroverov, T. A. Keith, R. Kobayashi, J. Normand, K. Raghavachari, A. P. Rendell, J. C. Burant, S. S. Iyengar, J. Tomasi, M. Cossi, J. M. Millam, M. Klene, C. Adamo, R. Cammi, J. W. Ochterski, R. L. Martin, K. Morokuma, O. Farkas, J. B. Foresman and D. J. Fox, Gaussian 16, Revision A.03 Gaussian Inc.: Wallingford CT, 2016.

C2 A. D. Becke, *Phys. Rev. A*, 1988, **38**, 3098-3100.

C3 J. P. Perdew, *Phys. Rev. B*, 1986, **33**, 8822-8824.

C4 D. Andrae, U. Häußermann, M. Dolg, H. Stoll and H. Preuß, *Theor. Chim. Acta*, 1990, **77**, 123-141.

C5 P. C. Hariharan and J. A. Pople, *Theor. Chim. Acta*, 1973, **28**, 213-222.

C6 J. Tomasi, B. Mennucci and R. Cammi, *Chem. Rev.*, 2005, **105**, 2999-3094.

C7 S. Grimme, S. Ehrlich and L. Goerigk, *J. Comput. Chem.*, 2011, **32**, 1456-1465.

C8 F. Weigend and R. Ahlrichs, *Phys. Chem. Chem. Phys.*, 2005, **7**, 3297-3305.

C9 Chemcraft - graphical software for visualization of quantum chemistry computations. Version 1.8, build 682. https://www.chemcraftprog.com

C10 Mercury 4.0: from visualization to analysis, design and prediction C. F. Macrae, I. Sovago, S. J. Cottrell, P. T. A. Galek, P. McCabe, E. Pidcock, M. Platings, G. P. Shields, J. S. Stevens, M. Towler and P. A. Wood, J. Appl. Cryst., **53**, 226-235, 2020 [DOI: 10.1107/S1600576719014092]

5.2 Computed Reaction Profiles.

Full free energy reaction profiles for competing methine C-H, C-C and methyl C-H activation are provided for [RhCp(SIDipp)], **A**, and [RhInd(SIDipp)], **A^{ind}**. (Figures S53 and S54 respectively). For both systems C-H activation can occur such that the hydride lies over (endo) or opposite (exo) the metallacycle formed. Figures S53 and S54 show the profiles that lead to the most stable of these products whereas details of how the alternative isomers are formed are given in Figures S55 and S56. Rotation of the iPr and indenyl ligands was also considered and the most stable rotamers are reported.

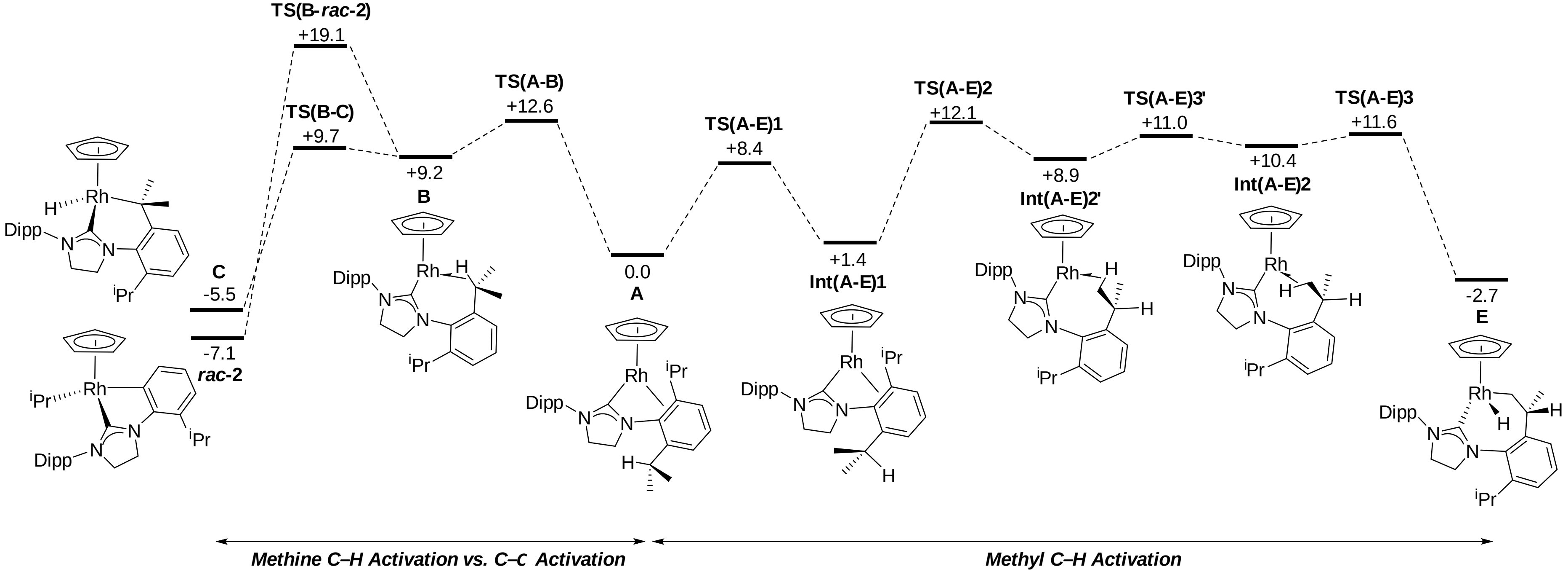

Figure S61. Computed free energy reaction profile (kcal/mol) for competing C-H and C-C bond activation in [Rh(Cp)(SIDipp)], **A**.

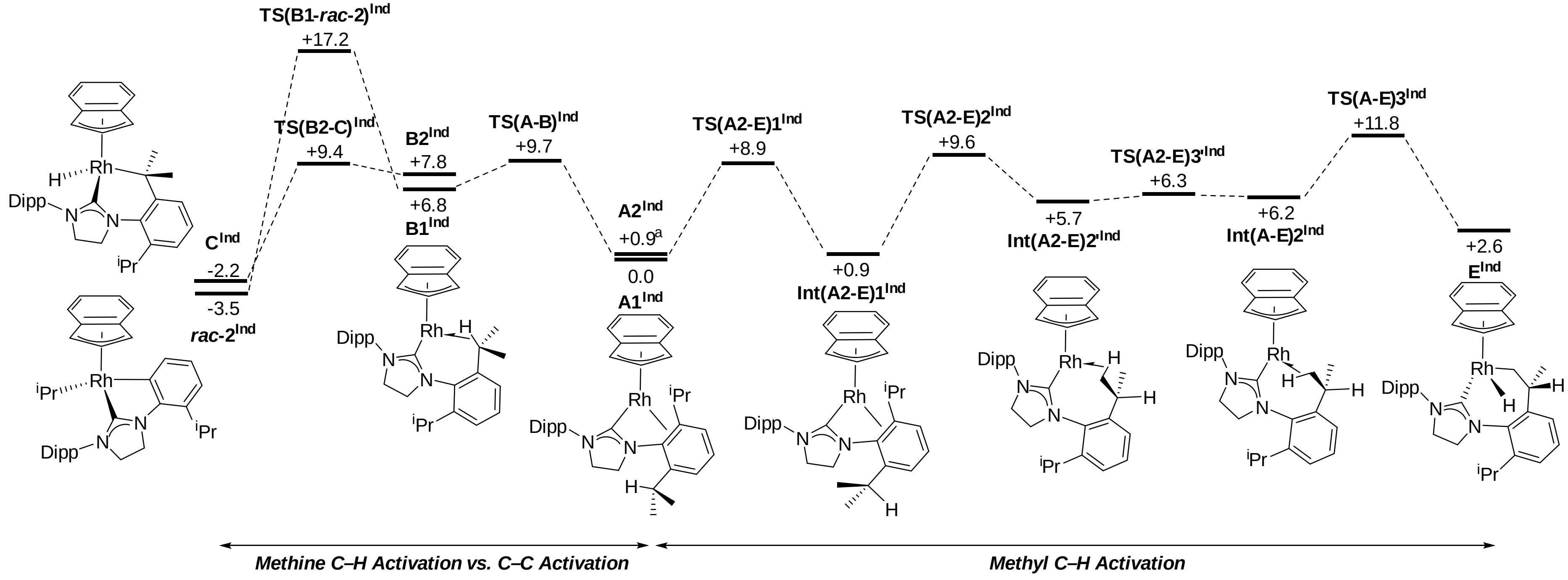

Figure S62. Computed free energy reaction profile (kcal/mol) for competing C-H and C-C bond activation in [Rh(Indenyl)(SIDipp)], **A**^{ind}. [a]**A1**^{ind} and **A2**^{Ind} and linked by **TS(A1-A2)**^{Ind} at +3.9 kcal/mol.

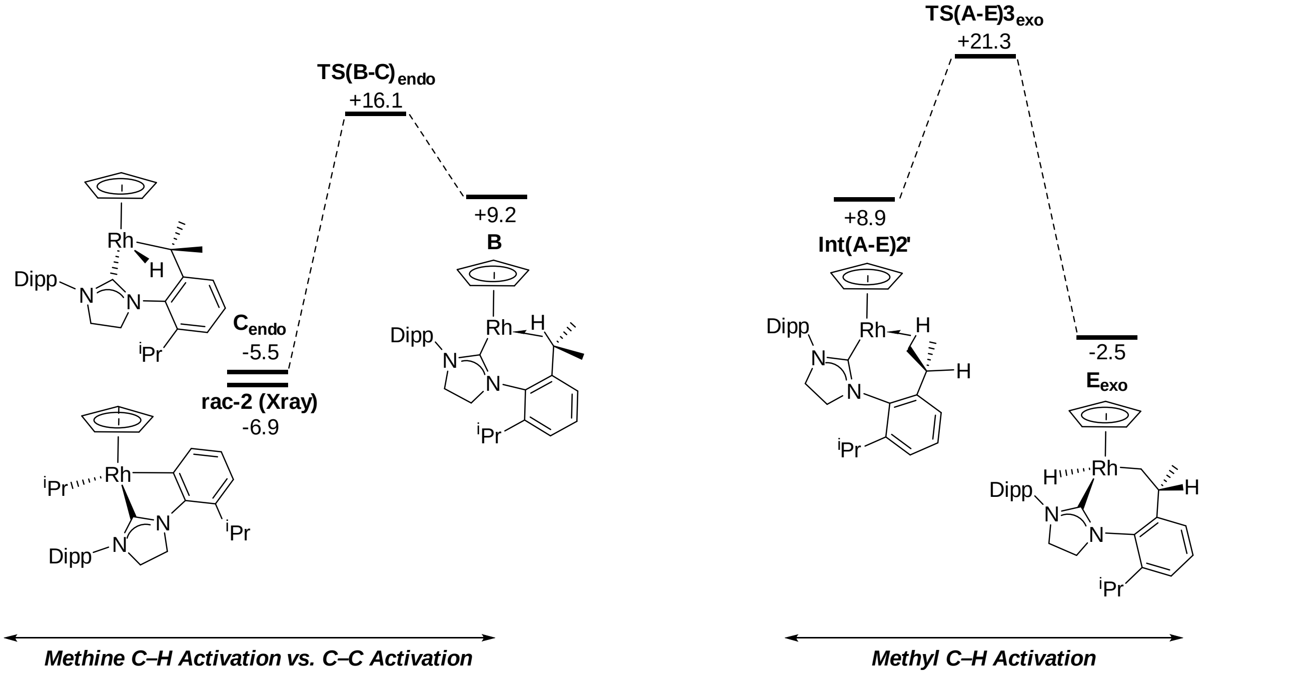

Figure S63. Computed free energy reaction profile (kcal/mol) for the formation of the alternative isomers of **C** and **E**. ***rac*-2 (X-ray)** has a slightly higher energy than the species shown in Figure S53 due to a different orientation of the iPr ligand.

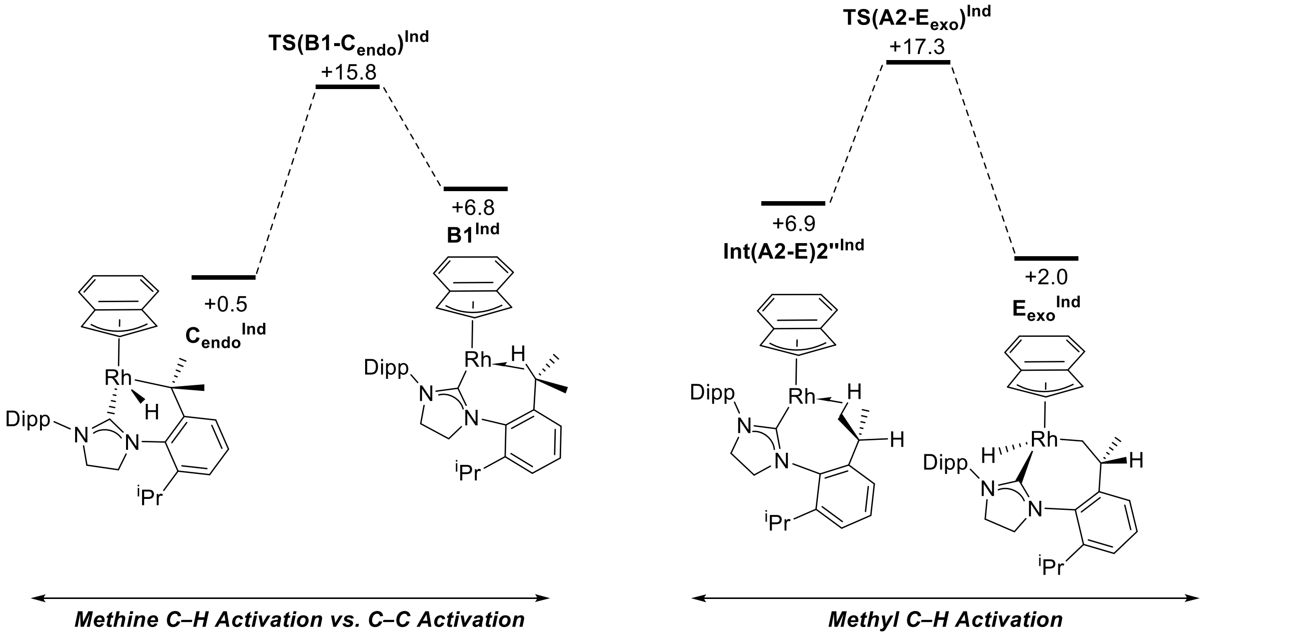

Figure S64. Computed free energy reaction profile (kcal/mol) for the formation of the alternative isomers of the alternative isomers of **C**$^{\mathbf{Ind}}$ and **E**$^{\mathbf{Ind}}$.

6 References

1. A. J. Arduengo, R. Krafczyk, R. Schmutzler, H. A. Craig, J. R. Goerlich, W. J. Marshall and M. Unverzagt, *Tetrahedron*, 1999, **55**, 14523-14534.
2. A. J. Arduengo, H. V. R. Dias, R. L. Harlow and M. Kline, *J. Am. Chem. Soc.*, 1992, **114**, 5530-5534.
3. F. Hanasaka, K.-i. Fujita and R. Yamaguchi, *Organometallics*, 2005, **24**, 3422-3433.
4. C.-I. Lee, N. A. Hirscher, J. Zhou, N. Bhuvanesh and O. V. Ozerov, *Organometallics*, 2015, **34**, 3099-3102.
5. R. Cramer, J. A. McCleverty and J. Bray, *Inorg. Synth.*, 1990, **28**, 86-88.
6. J. A. McCleverty, G. Wilkinson, L. G. Lipson, M. L. Maddox and H. D. Kaesz, *Inorg. Synth.*, 1990, **28**, 84-86.
7. C. N. Garon, D. I. McIsaac, C. M. Vogels, A. Decken, I. D. Williams, C. Kleeberg, T. B. Marder and S. A. Westcott, *Dalton Trans.*, 2009, 1624-1631.
8. M. Graf, H.-C. Böttcher, P. Mayer and M. Scheer, *Z. Anorg. Allg. Chem.*, 2017, **643**, 1323-1325.
9. K. Moseley, J. W. Kang and P. M. Maitlis, *J. Chem. Soc. A*, 1970, 2875-2883.
10. V. B. Kharitonov, S. A. Runikhina, Y. V. Nelyubina, D. V. Muratov, D. Chusov and D. A. Loginov, *Chem.-Eur. J.*, 2021, **27**, 10903-10912.
11. C. E. Tucker, J. Davidson and P. Knochel, *The Journal of Organic Chemistry*, 1992, **57**, 3482-3485.
12. S. Pereira and M. Srebnik, *Tetrahedron Lett.*, 1996, **37**, 3283-3286.
13. G. Sheldrick, *Acta Crystallogr. A*, 2015, **71**, 3-8.
14. O. V. Dolomanov, L. J. Bourhis, R. J. Gildea, J. A. K. Howard and H. Puschmann, *J. Appl. Crystallogr.*, 2009, **42**, 339-341.
15. K. J. Evans, P. A. Morton, C. Luz, C. Miller, O. Raine, J. M. Lynam and S. M. Mansell, *Chem.-Eur. J.*, 2021, **27**, 17824-17833.
